# Sarcophyolides B–E, New Cembranoids from the Soft Coral *Sarcophyton elegans*

**DOI:** 10.3390/md11093186

**Published:** 2013-08-26

**Authors:** Zhifang Xi, Wei Bie, Wei Chen, Dong Liu, Leen van Ofwegen, Peter Proksch, Wenhan Lin

**Affiliations:** 1 State Key Laboratory of Natural and Biomimetic Drugs, Peking University, Beijing 100191, China; E-Mails: xzf198007@163.com (Z.X.); winding814@163.com (W.B.); chenwei@bjmu.edu.cn (W.C.); liudong_1982@126.com (D.L.); 2 Chemical Analysis Department, Beijing Entry-Exit Inspection and Quarantine Bureau, Beijing 100026, China; 3 Naturalis Biodiversity Center, National Museum of Natural History, Leiden 2300, The Netherlands; E-Mail: ofwegen@yahoo.com; 4 Institute of Pharmaceutical Biology and Biotechnology, Heinrich-Heine University, 40225 Duesseldorf, Germany; E-Mail: proksch@uni-duesseldorf.de

**Keywords:** soft coral, *Sarcophyton elegans*, sarcophyolide, cembranoid, cytotoxicity

## Abstract

Four new cembrane-type diterpenoids, sarcophyolides B–E (**1**–**4**), along with 11 known analogues were isolated from the soft coral *Sarcophyton elegans*. The structures of new compounds **1**–**4** were established on the basis of spectroscopic analysis and chemical conversion. The new cembranoids sarcophyolides B (**1**) and lobocrasol were found to exhibit potent inhibition against A2780 human ovarian tumor cells.

## 1. Introduction

**Figure 1:**
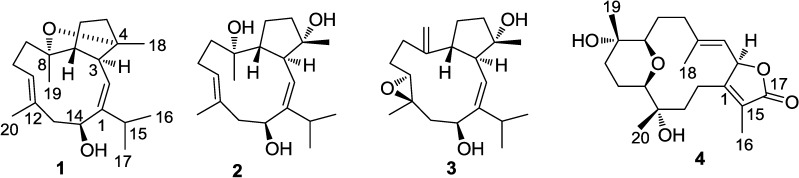
Structures of sarcophyolides B−E (**1**−**4**).

Since the first cembranoid (+)-cembrene was reported five decades ago, numerous cembranoids have been isolated from marine organisms, plants, and insects [1,2,3]. Their basic structural patterns typically featured a common 14-membered carbocyclic nucleus and unconventional cembranoids containing a 12-membered carbon skeleton or 13-membered variants. Some of the typical terpenoids are known as chemical defense tools to protect soft corals against natural predators, as feeding deterrents or act by virtue of their toxicity [4,5,6]. From a pharmaceutical point of view, cembranoids have been reported to exhibit various biological activities, such as having antitumor, ichthyotoxic, antiinflammatory, neuroprotective, antibacterial, antiangiogenic, antimetastatic, and antiosteoporotic properties [7,8,9,10,11,12,13]. Soft corals, belonging to the genus *Sarcophyton* (Alcyoniidae), are well recognized as a rich source of macrocyclic cembrane-type diterpenoids and biscembranoids. The structural patterns of cembranoids from the genus *Sarcophyton* vary notably due to geographic location and species differentiation [14]. It is a challenging work to uncover new natural products from known species of marine organisms distributed in new locations. In our continuing search for the chemical diversity from the soft corals inhabited in various locations of South China Sea, the specimen *Sarcophyton elegans* was collected. Primary HPLC-ESIMS and ^1^H NMR examinations on the EtOAc extracts revealed the spectroscopic signals representing a diverse array of terpenoids. Further chromatographic separation and purification resulted in the isolation of four new cembranoids (Figure 1) in addition to 11 known analogues.

## 2. Results and Discussion

### 2.1. Structural Elucidation of New Compounds

**Figure 2:**
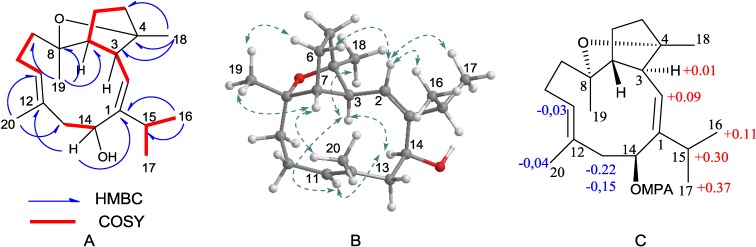
Key COSY, HMBC (**A**), NOE (**B**), and Δδ*^RS^* values (**C**) of **1**.

Sarcophyolide B (**1**) was isolated as a colorless oil with the molecular formula of C_20_H_32_O_2_ based on the high resolution electrospray ionization mass spectroscopy (HRESIMS) and nuclear magnetic resonance (NMR) data, implying five degrees of unsaturation. The IR absorptions at 3406 and 1604 cm^−1^ suggested the presence of hydroxy and olefinic groups. The ^1^H NMR displayed the signals for five methyls, two olefinic protons at δ_H_ 5.26 (1H, d, *J* = 11.5 Hz, H-2) and 5.49 (1H, dd, *J* = 3.0, 5.0 Hz, H-11), a hydroxymethine δ_H_ 5.04 (1H, brd, *J* = 9.0 Hz, H-14), and a number of alkyl protons for methylene and methine groups. The ^13^C NMR and distortionless enhancement by polarization transfer (DEPT) spectra exhibited a total of 20 carbon resonances, involving four olefinic carbons and three oxygen-bearing sp^3^ carbons. Diagnostic NMR data (Table 1 and Table 2) through COSY and heteronuclear multiple quantum coherence (HMQC) analyses indicated compound **1** to be a cembrane-based diterpenoid. The COSY relationships connected the protons to form three subunits from C-2 to C-7, C9 to C-11, and C-13 to C-14, in addition to an isopropyl group. The connectivity of the subunits was accomplished by the HMBC correlations. The observed HMBC interactions from the methyl protons of isopropyl group (δ_H_ 1.12 and 1.13, d) to an olefinic carbon at δ_C_ 150.6 (qC, C-1) and, in turn, the olefinic proton H-2 correlating to the methine carbon C-15 (δ_C_ 26.9, CH), indicated a double bond to be resided at C-1/C-2, while an isopropyl group is positioned at C-1. The HMBC relationships from H_3_-18 (δ_H_ 1.19, s) to C-3 (δ_C_ 53.2, CH), C-4 (δ_C_ 87.6, qC), and C-5 (δ_C_ 32.8, CH_2_); from H-3 (δ_H_ 3.11, brd, *J* = 11.5 Hz) to C-1, C-4, C-7, and C-8 (δ_C_ 82.8, qC); and from H-7 (δ_H_ 1.90) to C-4 and C-2 (δ_C_ 120.9, CH) revealed a capnosane-based cembranoid bearing a 3,7-cyclopentane ring [15], in which an oxygen atom and a methyl group were co-positioned at C-4. Additional HMBC relationships were conducted to assign the linkage of a methyl group H_3_-19 (δ_H_ 1.23, s) at oxygenated carbon C-8, while the second olefinic group was resided at C-11/C-12 (Figure 2A). A hydroxy group was evident to be located at C-14 (δ_C_ 72.9, CH) according to the COSY relationship between a D_2_O exchangeable proton at δ_H_ 4.60 (br) and H-14, while H-14 coupled to C-14 in HMQC. The above functional groups are accounted for four degrees of unsaturation, the remaining site is, thus, assumed to be contributed by an ether bridge across C-4 and C-8.

**Table 1:** ^1^H NMR data for sarcophyolides B−E (**1**–**4**) in CDCl_3_ (δ in ppm, *J* in Hz).

No.	1	2	3	4
2	5.26 d (11.5)	5.28 d (9.5)	5.09 d (10.4)	5.50 d (10.0)
3	3.11 brd (11.5)	2.64 dd (9.5,10.5)	2.77 dd (10.4,10.4)	5.00 d (10.0)
5	1.55 m	1.76 m	1.85 m	2.16 m
	1.74 m	1.78 m	1.80 m	2.20 m
6	1.68 m	1.35 m	1.70 m	1.43 m
	1.74 m	1.74 m	1.82 m	1.96 m
7	1.90 m	1.97 ddd (8.5,10.0,10.5)	2.50 m	3.02 brd (10.0)
9	1.90 m	1.62 ddd (3.0,5.0,14.0)	2.25 m	1.48 m
	1.92 m	1.82 brdd (10.0,14.0)	2.20 m	1.46 m
10	2.00 m	2.05 m	2.45 m	1.34 m
	2.02 m	2.41 ddd (8.0, 10.0, 12.0)	1.40 m	1.67 m
11	5.49 dd (3.0,5.0)	5.36 dd (4.5, 8.0)	2.88 dd (4.2, 9.8)	3.14 d (8.5)
13	2.00 dd (9.0,11.5)	2.09 dd (11.5, 13.0)	2.13 d (13.3)	1.48 m
	2.62 brd (11.5)	2.50 dd (3.0, 13.0)	1.50 dd (10.7, 13.3)	1.73 m
14	5.04 brd (9.0)	4.86 dd (3.0, 11.5)	3.83 d (10.7)	1.93 m
				2.45 m
15	2.68 qq (7.0, 7.0)	2.56 qq (7.0, 7.0)	2.49 qq (6.8, 6.8)	
16	1.12 d (7.0)	1.09 d (7.0)	1.03 d (6.9)	
17	1.13 d (7.0)	1.15 d (7.0)	1.14 d (6.9)	1.73 s
18	1.19 s	1.11 s	1.17 s	1.78 s
19	1.23 s	1.15 s	4.96 brs	0.98 s
			4.91 brs	
20	1.60 s	1.73 s	1.49 s	0.98 s

**Table 2:** ^13^C NMR data for sarcophyolides B−E (**1**–**4**) in CDCl_3_ (δ in ppm, *J* in Hz).

No.	1	2	3	4
1	150.6 qC	149.8 qC	150.9 qC	165.8 qC
2	120.9 CH	126.2 CH	124.9 CH	80.1 CH
3	53.2 CH	49.7 CH	50.8 CH	120.0 CH
4	87.6 qC	81.5 qC	81.3 qC	144.2 qC
5	32.8 CH_2_	39.7 CH_2_	40.1 CH_2_	36.2 CH_2_
6	21.1 CH_2_	24.4 CH_2_	25.4 CH_2_	24.7 CH_2_
7	49.6 CH	56.3 CH	54.6 CH	83.9 CH
8	82.8 qC	74.7 qC	147.4 qC	68.8 qC
9	43.5 CH_2_	34.2 CH_2_	24.1 CH_2_	40.8 CH_2_
10	24.0 CH_2_	23.4 CH_2_	27.5 CH_2_	23.4 CH_2_
11	133.0 CH	129.9 CH	59.5 CH	80.5 CH
12	128.0 qC	131.0 qC	58.1 qC	71.8 qC
13	43.5 CH_2_	43.0 CH_2_	45.6 CH_2_	37.3 CH_2_
14	72.9 CH	70.8 CH	67.8 CH	20.6 CH_2_
15	26.9 CH	26.4 CH	26.7 CH	121.7 qC
16	25.3 CH_3_	24.7 CH_3_	24.4 CH_3_	9.0 CH_3_
17	25.7 CH_3_	26.4 CH_3_	26.4 CH_3_	174.9 qC
18	19.1 CH_3_	23.0 CH_3_	24.1 CH_3_	16.7 CH_3_
19	25.1 CH_3_	31.7 CH_3_	111.2 CH_2_	20.5 CH_3_
20	20.0 CH_2_	18.8 CH_3_	17.1 CH_3_	23.9 CH_3_

The relative configuration of **1** was established on the basis of NOE relationships and *J* values. The NOE interactions between H-2 and H_3_-16 and H_3_-17 were assignable to 1*Z* geometry, whereas 11*E* was inferred from the NOE interactions between H-11 and H-13a (δ_H_ 2.62), and between H_3_-20 and H_2_-10. The NOE interactions between H-3 and H-14 and H_3_-18 indicated that H-3 is spatially approximated to H-14 and H_3_-18. Additional NOE relationships between H-2 and H-6a (δ_H_ 1.68), and between H_3_-19 and H-6b (δ_H_ 1.74) and H-7 (Figure 2B), in association with the absence of NOE interaction between H-2 and H-3, allowed to establish the relative configurations of the stereogenic centers in cyclopentane ring and the orientation of ether bridge. The absolute configuration of C-14 in **1** was determined by Mosher’s method. Esterification of **1** with (*R*)- and (*S*)-MPA yielded 14-(*R*)-MPA and 14-(*S*)-MPA esters, respectively. Based on MPA rules [16], the chemical shift difference (Δδ^RS^ = δ^R^ − δ^S^) reflected the absolute configuration of C-14. Analyses of the Δδ^RS^ values (Figure 2C) resulted in 14*S* configuration. Based on the established relative configurations of **1** and the NOE interaction of the protons related to H-14, the absolute configurations of the remaining chiral carbons were supposed to be 3*S*, 4*S*, 7*R*, and 8*R*.

**Figure 3:**
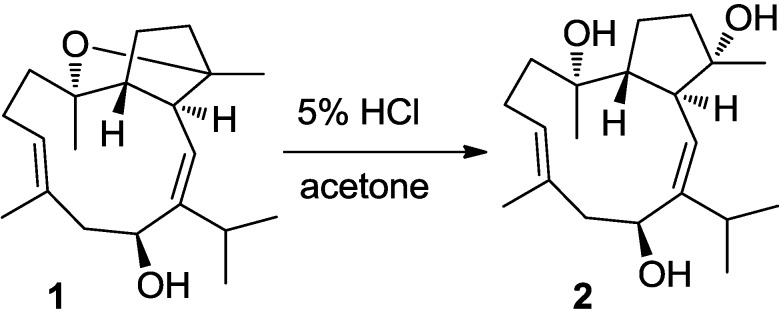
Conversion of **1** to **2** under acidic condition.

The NMR data of sarcophyolide C (**2**) closely resembled those of compound **1**, while 2D NMR data analysis established the structure of **2** to be a homolog of **1**. The major difference was found concerning the upfield-shifted C-4 (δ_C_ 81.5) and C-8 (δ_C_ 74.7) and the molecular weight of **2** having 18 amu more than that of **1**, while the degrees of molecular unsaturation in **2** are four instead of five, based on the HRESIMS data (*m/z* 345.2406 [M + Na]^+^). These findings disclosed the structure of **2** to be a 4,8-dihydroxylated derivative of **1**. The closely similar NOE interactions of **2** and **1** in association with the chemical conversion from **1** to **2** under acidic solution (Figure 3) indicated the configurations of **2** to be the same as those of **1**.

**Figure 4:**
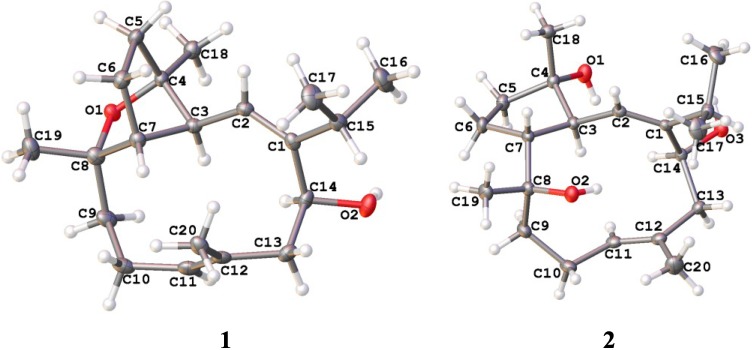
ORTEP depiction for X-ray crystal structures of **1** and **2**.

The absolute configurations of the stereogenic centers in **1** and **2** were further proved by their single-crystal X-ray diffraction analysis using Flack’s method (Figure 4).

**Figure 5:**
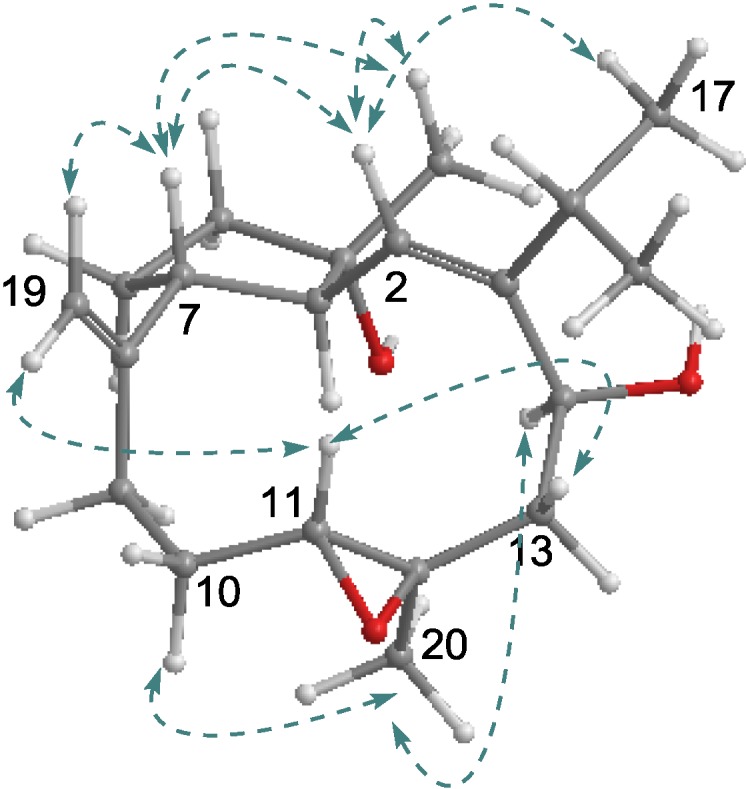
Key NOE correlations of **3**.

The HRESIMS data (*m/z* 343.2239 [M + Na]^+^) of sarcophyolide D (**3**) was in accordance with a molecular formula of C_20_H_32_O_3_ with five degrees of unsaturation. The NMR data of compound **3** were compatible to those of sarcophytol L [15], except for the presence of two olefinic bonds instead of three bonds in the known analog. Analysis of ^13^C NMR in association with 2D NMR data revealed **3** presenting two epoxy carbons (δ_C_ 58.1 and 59.5), residing at C-11 and C-12 according to the HMBC correlations of H_3_-20 (δ_H_ 1.49, s) to C-11 (δ_C_ 59.5, CH), C-12 (δ_C_ 58.1, qC), and C-13 (δ_C_ 45.6, CH_2_). Thus, the structure of **3** was determined as an 11,12-epoxidated sarcophytol L. The relative configurations of the stereogenic centers in **3** from C-1 to C-7 were in agreement with those of sarcophytol L due to the similar NOE and NMR data. Additional NOE relationships from H-14 to H_3_-20 and H-3, and from H-11 to H_3_-16 and H-13a (Figure 5) assigned a *trans* geometry of the epoxy group, while H_3_-20 is oriented in the same face as H-14.

**Figure 6:**
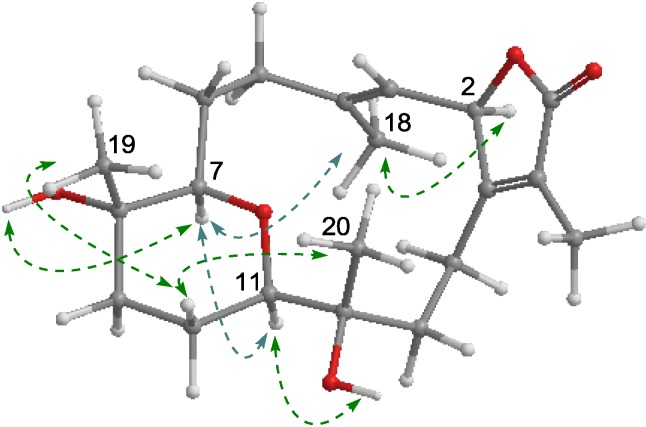
Key NOE correlation of **4**.

**Figure 7:**
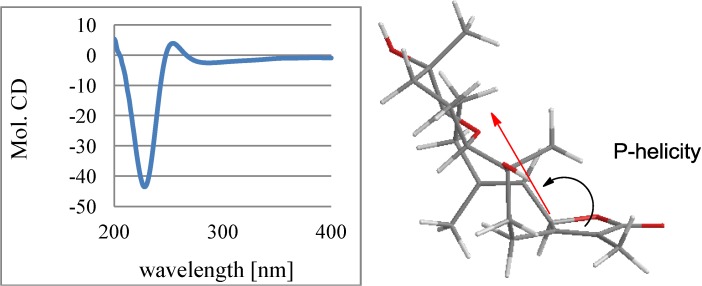
CD effects and P-helicity of **4**.

The 2D NMR (COSY, HMQC, and HMBC) data analysis revealed the gross structure of sarcophyolide E (**4**) closely related to a known cembranoid derived from sarcophtolide through oxymercuration [17]. The only difference was due to C-12 in which a quaternary carbon (δ_C_ 71.8) of **4** was replaced by a methine carbon of the known analog. The hydroxylated C-12 was supported by its HMBC correlations with H_3_-20 (δ_H_ 0.98, s) and a hydroxyl proton OH-12 (δ_H_ 4.46, s). The relative configurations of **4** were determined on the basis of NOE interactions. The NOE correlations between H-7 and H-11 and H_3_-18, and between H_3_-18 and H-2 informed a *cis*-geometry of the epoxy bond and 3*E* of the olefinic bond. In addition, the NOE interactions between H-7 and OH-8 (δ_H_ 4.40, s) and between H-11 and OH-12 in association with the absence of the interactions of H-11/H_3_-20 and H-7/H_3_-19 revealed the opposite orientation of H-7 and H-11 toward their vicinal methyl groups (Figure 6). Based on the CD rule for α,β-unsaturated-γ-lactone [18], the Cotton effects due to the *n*→π* and π*→π* transitions of the α,β-unsaturated lactone chromophore correlated directly to the absolute configuration of the stereogenic center at C(γ). Thus, the positive Cotton effects for *n*→π* (252 nm) and negative π*→π* (226 nm) of **4** (Figure 7) indicated that it follows *p*-helicity rule, demonstrating 2*R* configuration.

Based on the spectroscopic analyses and comparison of the NMR data with those reported in literature, 11 known cembranoids were identical to: sarcophytol L [19], 13α-hydroxysarcophytol L [19], sarcophyolide A [20], sarcophine [21], sarcophinone [22,23], 7α-hydroxy-Δ^8(^^19)^-deepoxysarcophine [24], 4β-hydroxy-Δ^2(3)^-sarcophine [24], 7α,8β-dihydroxydeepoxysarcophine [25], 1,15β-epoxy-2-*epi*-16-deoxysarcophine [3], sarcophytol Q [26], and lobocrasol [27]. Lobocrasol presented as a unique skeleton that was isolated from soft coral for the second time.

### 2.2. Cytotoxic Results

A bioassay guiding fractionation revealed the EtOAc extract showing selective inhibition against human ovarian carcinoma cell line A2780 (IC_50_ = 4.9 μg/mL), but it exhibited weak inhibition against human lung adenocarcinoma epithelial cell line A549 (IC_50_ = 23.7 μg/mL), human gastric carcinoma cell line BGC823 (IC_50_ = 20.4 μg/mL), human hepatoma cell line Bel7402 (IC_50_ = 19.1 μg/mL), and human colonic carcinoma cell line HCT-8 (IC_50_ = 19.5 μg/mL). In the additional tests of the pure compounds, **1** and lobocrasol showed significant inhibition against A2780 with IC_50_ values of 2.92 and 3.37 μM, respectively, whereas the other cembranoids exhibited weak activity (IC_50_ > 10 μg/mL). Taxol was used as a positive control, which displayed the inhibition against A2780 with IC_50_ of 14.45 μM.

## 3. Experimental Section

### 3.1. General

Optical rotations were measured on a Perkin-Elmer 243B polarimeter. IR spectra were recorded on a Thermo Nicolet Nexus 470 FTIR spectrometer. ^1^H and ^13^C NMR and 2D NMR spectra were measured on an Avance-500 FT 500 MHz NMR spectrometer using TMS as an internal standard, while δ values are expressed in parts per million (ppm), and *J* values are reported in Hertz (Hz). HRESIMS data were obtained from Bruker APEX IV instrument. Low pressure column chromatography was carried using silica gel (160–200 and 200–300 mesh). The GF_254_ silica gel for TLC was provided by Qingdao Marine Chemistry Co., Ltd. (Qingdao, China).

### 3.2. Animal Material

The soft coral *Sarcophyton elegans* was collected from Xidao Island, Hainan, China, in 2002, and kept frozen until extraction. The specimen was identified by Dr. Leen van Ofwegen (National Museum of National History, Naturalis). The soft coral (HSE-17) was deposited at State Key Laboratory of Natural and Biomimetic Drugs, Peking University, China.

### 3.3. Extraction and Isolation

The frozen soft coral *Sarcophyton elegans* (3.5 kg, wet weight) was homogenized and extracted with EtOH. The concentrated extract was desalted through dissolving in MeOH to yield a residue (100 g) after evaporation. This residue was defatted by partitioning between H_2_O and petroleum ether, and then the H_2_O fraction was extracted with EtOAc. The EtOAc fraction (7.4 g) was subjected to Si gel column chromatography eluting with a gradient of petroleum ether (PE)-acetone to obtain eight subfractions (SF1–SF8). SF2 (1.2 g) was subsequently subjected to Si gel column chromatography eluting with PE–EtOAC (5:1) to yield **1** (9.0 mg), **3** (4.2 mg), and **2** (4.8 mg). SF3 (1.0 g) was treated by the same process as SF2 to yield **4** (2.3 mg). From SF7 (890 mg) and SF8 (320 mg) fractions, **5**(5.6), **15** (3.2 mg), **22** (8.6 mg), **6** (5.5 mg), **8** (7.2 mg), **7** (8.8 mg), **9** (2.3 mg), **10** (2.5 mg), **12** (4.5 mg), **11** (6.8 mg), **13** (3.2 mg), and **14** (5.4 mg) were separated upon semipreparative HPLC (C_18_, 5 μm) using a mobile phase of MeOH–H_2_O (65:35).

Sarcophyolide **B** (**1**). Colorless oil. [α]_D_^25^ +16.7 (*c* 6.0, CHCl_3_). IR ν_max_ (KBr) 3406, 2954, 2925, 1604, 1459, 1189, 1071 cm^−1^; for ^1^H and ^13^C NMR data, see Table 1 and Table 2; HRESIMS *m/z* 305.2473 [M + H]^+^, 327.2292 [M + Na]^+^ (calcd for C_20_H_32_O_2_Na, 327.2294).

Sarcophyolide C (**2**). Colorless oil. [α]_D_^25^ −62.0 (*c* 4.9, CHCl_3_). IR ν_max_ (KBr) 3386, 2958, 2927, 1604, 1189, 1079 cm^−1^; ^1^H and ^13^C NMR data, see Table 1 and Table 2; HRESIMS *m/z* 345.2406 [M + Na]^+^ (calcd for C_20_H_34_O_3_Na, 345.2400).

Sarcophyolide D (**3**). Colorless oil. [α]_D_^25^ −2.5 (*c* 2.9, CHCl_3_). IR ν_max_ (KBr) 3370, 2959, 2961, 2860, 1610, 1389, 1298, 1113, 1050 cm^–1^; ^1^H and ^13^C NMR data, see Table 1 and Table 2; HRESIMS *m/z* 343.2239 [M + Na]^+^ (calcd for C_20_H_32_O_3_Na, 343.2239).

Sarcophyolide E (**4**). Colorless oil. [α]_D_^25^ +4.4 (*c* 3.1, CHCl_3_). IR ν_max_ (KBr) 3464, 2964, 2937, 2253, 1725, 1673, 1443, 1378, 1103 cm^–1^; ^1^H and ^13^C NMR data, see Table 1 and Table 2; HRESIMS *m/z* 373.1980 [M + Na]^+^ (calcd for C_20_H_30_O_5_Na, 373.1980).

### 3.4. Cytotoxic Bioassays

The tetrazolium-based colorimetric assay (MTT assay) was used for *in vitro* assay of cytotoxicity from HCT-8, Bel-7402, BGC-823, A549, and A2780 tumor cell lines.

### 3.5. Mosher Reaction

Compound **1** (0.01 mmol), together with DMAP (4-dimethylaminopyridine, 0.01 mmol) and DCC (dicyclohexylcarbodiimide, 0.01 mmol), were dissolved in methylene dichloride (2 mL) at 0 °C, and then (*R*)- or (*S*)-MPA (0.01 mmol) was added to the solution. After stirring at room temperature for 24 h, the mixture was evaporated under reduced pressure to obtain a residue, which was separated using a reversed phase semipreparative HPLC with 95% CH_3_CN–H_2_O as a mobile phase to yield (*R*)-MPA ester or (*S*)-MPA ester.

### 3.6. Chemical Conversion

To a solution of **1** (1 mg/mL) in acetone 5% HCl (0.2 mL) was added. After stirring for 2 h at room temp. the reaction mixture was extracted with EtOAc (0.5 mL). The organic layer was concentrated to yield a product **1a**. Specific rotations, ESIMS, Rf-values of TLC, and ^1^H NMR data indicated the structure of **1a** to be identical to **2**.

**1a**. Colorless oil. [α]_D_^25^ −60.0 (*c* 0.32, CHCl_3_). ^1^H NMR δ (CDCl_3_) 5.29 (1H, d, *J* = 9.5 Hz, H-2), 2.63 (1H, dd, *J* = 9.5,10.0 Hz, H-3), 1.74, 1.76 (m, H_2_-5), 1.33, 1.76 (m, H_2_-6), 1.97 (1H, m, H-7), 1.62, 1.83 (m, H_2_-9), 2.04, 2.40 (m, H_2_-10), 5.35 (1H, dd, *J* = 4.5, 8.0 Hz, H-11), 2.08, 2.50 (m, H_2_-13), 4.85 (1H, dd, *J* = 2.0, 10.0 Hz, H-14), 2.55 (1H, m, H-15), 1.08 (3H, d, *J* = 7.5 Hz, H_3_-16), 1.15 (3H, d, *J* = 7.5 Hz, H_3_-17), 1.10 (3H, s, H_3_-18), 1.15 (3H, s, H_3_-19), 1.72 (3H, s, H_3_-20), ESIMS *m/z* 345.2 [M + Na]^+^.

## 4. Conclusions

Present work provided a number of new cembranoids, which enriched the cembranoid family. Capnosane-type cembranoids with 3,7-fused carbobicyclic skeleton are a group of uncommon derivatives, derived from soft corals, while the unique ether bridge across C-4/C-8 in **1** is reported for the first time. These findings implied that the species of genus *Sarcophyton* are potential sources, waiting for the discovery of structurally unique chemical diversity.

## Supplementary Files

Supplementary File 1 Supplementary Information (PDF, 4301 KB)
