# Supplementary material for: Sarcophyolides B–E, New Cembranoids from the Soft Coral Sarcophyton elegans
Source: Mar Drugs. 2013 Aug 26;11(9):3186–96. doi: 10.3390/md11093186 (PMC3801120; doi:10.3390/md11093186)
Supplement: Supplementary File 1 — Supplementary Information (PDF, 4301 KB) [file marinedrugs-11-03186-s001.pdf]

Supplementary Information

Table of contents

The structures and spectroscopic data of known analogues.
Figure S1. ^{1}H NMR data of sarcophyolide B (**1**).
Figure S2. ^{13}C NMR data of sarcophyolide B (**1**).
Figure S3. COSY spectrum of sarcophyolide B (**1**).
Figure S4. HMQC spectrum of sarcophyolide B (**1**).
Figure S5. HMBC spectrum of sarcophyolide B (**1**).
Figure S6. NOESY spectrum of sarcophyolide B (**1**).
Figure S7. IR spectrum of sarcophyolide B (**1**).
Figure S8. HRMS spectrum of sarcophyolide B (**1**).
Figure S9. ^{1}H NMR data of sarcophyolide C (**2**).
Figure S10. ^{13}C NMR data of sarcophyolide C (**2**).
Figure S11. COSY spectrum of sarcophyolide C (**2**).
Figure S12. HMQC spectrum of sarcophyolide C (**2**).
Figure S13. HMBC spectrum of sarcophyolide C (**2**).
Figure S14. NOESY spectrum of sarcophyolide C (**2**).
Figure S15. IR spectrum of sarcophyolide C (**2**).
Figure S16. HRMS spectrum of sarcophyolide C (**2**).
Figure S17. ^{1}H NMR data of sarcophyolide D (**3**).
Figure S18. ^{13}C NMR data of sarcophyolide D (**3**).
Figure S19. COSY spectrum of sarcophyolide D (**3**).
Figure S20. HMQC spectrum of sarcophyolide D (**3**).
Figure S21. HMBC spectrum of sarcophyolide D (**3**).
Figure S22. NOESY spectrum of sarcophyolide D (**3**).
Figure S23. IR spectrum of sarcophyolide D (**3**).
Figure S24. HRMS spectrum of sarcophyolide D (**3**).
Figure S25. ^{1}H NMR data of sarcophyolide E (**4**).
Figure S26. ^{13}C NMR data of sarcophyolide E (**4**).
Figure S27. COSY spectrum of sarcophyolide E (**4**).
Figure S28. HMQC spectrum of sarcophyolide E (**4**).
Figure S29. HMBC spectrum of sarcophyolide E (**4**).
Figure S30. NOESY spectrum of sarcophyolide E (**4**).
Figure S31. IR spectrum of sarcophyolide E (**4**).
Figure S32. HRMS spectrum of sarcophyolide E (**4**).
Figure S33. ORTEP depiction for X-ray crystal structure of **1**.
- **Table 1.** Crystal data and structure refinement for compound **1**.
- **Table 2.** fractional atomic coordinates and equivalent isotropic displacement parameters for compound **1**.
- **Table 3.** Anisotropic displacement parameters for compound **1**.
- **Table 4.** Bond lengths for compound **1**.
- **Table 5.** Bond angles for compound **1**.
- **Table 6.** Torsion angles for compound **1**.
- **Table 7**. Hydrogen atom coordinates and isotropic displacement parameters for compound **1**.

Figure S34. ORTEP depiction for X-ray crystal structure of **2**.
- **Table 1.** Crystal data and structure refinement for compound **2**.
- **Table 2.** fractional atomic coordinates and equivalent isotropic displacement parameters for compound **2**.
- **Table 3.** Anisotropic displacement parameters for compound **2**.
- **Table 4.** Bond lengths for compound **2**.
- **Table 5.** Bond angles for compound **2**.
- **Table 6.** Torsion angles for compound **2**.
- **Table 7.** Hydrogen atom coordinates and isotropic displacement parameters for compound **2**.

The structures and spectroscopic data of known analogues

1. Sarcophytol L (5):

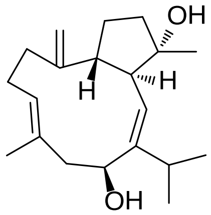

Colorless oil, $[\alpha]_D^{32.5}$ -109° (*c* 4.6, $CHCl_3$); ^{1}H-NMR ($CDCl_3$, 500 MHz), δ 5.17 (1H, dd), 5.02 (1H, d), 4.87 (1H, brs), 4.86 (1H, brs), 4.54 (1H, dd), 2.54 (1H, d), 2.50 (1H, dd), 2.48 (1H, dd), 2.40 (1H, ddd), 2.38 (1H, dd), 2.27 (1H, ddd), 2.20 (1H, dd), 2.11 (1H, dd), 1.77 (1H, dd), 1.76 (1H, dd), 1.71 (3H, s), 1.65 (1H, ddd), 1.53 (1H, ddd), 1.27 (3H, d), 1.12 (3H, s), 1.09 (3H, d);^{13}C-NMR ($CDCl_3$, 125 MHz) δ 149.2 (s), 148.7 (s), 131.0 (s), 126.5 (d), 125.2 (d), 111.5 (t), 81.3 (s), 71.2 (d), 54.9 (d), 50.1 (d), 44.3 (t), 40.2 (t), 27.9 (t), 27.3 (t), 26.5 (q), 26.0 (d), 25.4 (t), 24.4 (q), 23.4 (q), 14.1 (q); EI-MS *m/z* 304 $[M]^+$.

2 13α-Hydroxysarcophytol L (6)

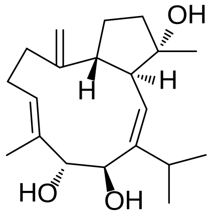

White powder. $[\alpha]_D^{20}$ -54° (c 2.3, $CHCl_3$); IR(KBr)v_{max} 3360, 2961, 2918, 2862, 1650, 1603, 1460, 1398, 1213, 1030 cm^{-1}; ^{1}H NMR ($CDCl_3$, 500 MHz) δ: 5.04 (1H, d, *J* = 10.0 Hz, H-2), 2.56 (1H, m, H-3), 1.80 (1H, m, H-5a), 1.72 (1H, m, H-5b), 1.88 (1H, m, H-6a), 1.79 (1H, m, H-6b), 2.41 (1H, m. H-7), 2.40 (1H, m, H-9a), 2.33 (1H, m, H-9b), 1.72 (1H, m, H-10a), 1.56 (1H, m, H-10b), 5.49 (1H, brt, *J* = 7.5 Hz, H-11), 4.53 (1H, d, *J* = 9.5 Hz, H-13), 3.83 (1H, d, *J* = 9.5 Hz, H-14), 2.56 (1H, qq, *J* = 6.8 Hz, H-15), 1.06 (3H, d, *J* = 6.8 Hz, H-16), 1.16 (3H, d, *J* = 6.8 Hz, H-17), 1.08 (3H, s, H-18), 4.80 (1H, brs, H-19a), 4.84 (1H, brs, H-19b), 1.68 (1H, s, H-20); ^{13}C NMR ($CDCl_3$, 125 MHz) δ: 146.8 (C-1), 120.7 (C-2), 50.6 (C-3), 81.5 (C-4), 40.5 (C-5), 27.7 (C-6), 54.4 (C-7), 149.5 (C-8), 27.5 (C-9), 25.5 (C-10), 127.3 (C-11), 134.5 (C-12), 71.4 (C-13), 76.7 (C-14), 27.7 (C-15), 24.1 (C-16), 26.5 (C-17), 23.2 (C-18), 111.7 (C-19), 15.0 (C-20); ESI-MS^- *m/z:* 319 $[M-H]^+$.

3 Sarcophyolide A (7)

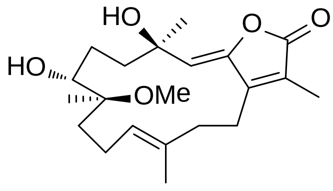

Colorless oil. $[\alpha]_D^{20}$ +5° (*c* 2.2, $CHCl_3$); IR(KBr)ν_{max} 3401, 2932, 2255, 1759, 1634, 1262, 1184, 1083 cm^{-1}; ^{1}H NMR (DMSO-d_6, 500 MHz) δ: 5.27 (1H, s, H-3), 1.88 (1H, dt, *J* = 6.8, 14.1 Hz, H-5a), 1.62 (1H, m, H-5b), 1.27-1.30 (2H, m, H_2-6), 3.16 (1H, brd, *J* = 7.9 Hz, H-7), 1.58 (1H, m, H-9a), 1.38 (1H,

m, H-9b), 2.10 (1H, m, H-10a), 1.80 (1H, m, H-10b), 4.97 (1H, dd , *J* = 6.4, 6.5 Hz, H-11), 2.25 (1H, m, H-13a), 2.12 (1H, m, H-13b), 2.59 (1H, m, H-14a), 2.48 (1H, m, H-14b), 1.84 (3H, s, H-16), 1.36 (3H, s, H-18), 0.94 (3H, s, H-19), 1.56 (3H, s, H-20), 3.04 (3H, s, OMe); ^{13}C NMR (DMSO-d_6, 125 MHz) δ: 153.2 (s, C-1), 147.2 (s, C-2), 119.3 (d, C-3), 72.2 (s, C-4), 40.0 (t, C-5), 25.9 (t, C-6), 74.1 (d, C-7), 79.0 (s, C-8), 33.1 (t, C-9), 21.9 (t, C-10), 129.2 (d, C-11), 130.7 (s, C-12), 38.4 (t, C-13), 21.9 (t, C-14), 122.7 (s, C-15), 170.8 (s, C-17), 8.9 (q, C-16), 31.0 (q, C-18), 19.5 (q, C-19), 16.0 (q, C-20), 49.2 (q, MeO); HR-ESI-MS *m/z* 387.2140 (calc. for $C_{21}H_{32}O_5Na$, 387.2140).

4 Sarcophine (8)

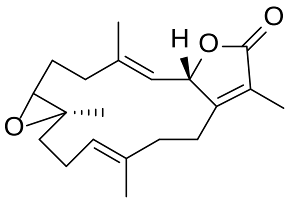

Colorless oil, $[\alpha]_D^{26}$ +97° (c 0.01, $CHCl_3$); ^{1}H-NMR ($CDCl_3$, 500 MHz), δ 5.56 (1H, d), 5.13 (1H, m), 5.03 (1H, d), 0.8 (1H, m), 2.67 (1H, m), 2.36 (1H, brs), 2.28 (1H, m), 2.07 (1H, m), 2.02 (1H, m), 1.96 (1H, m), 1.92 (1H, d), 1.89 (3H, s), 1.85 (3H, s), 1.66 (1H, d), 1.62 (3H, s), 1.28 (3H, s), 1.09 (1H, m);^{13}C-NMR ($CDCl_3$, 125 MHz) δ 174.6 (s), 162.3 (s), 144.0 (s), 135.5 (s), 124.9 (d), 122.8 (s), 120.6 (d), 78.8 (d), 61.4 (d), 59.9 (s), 39.0 (t), 37.4 (t), 36.4 (t), 27.5 (t), 25.2 (t), 23.0 (t), 22.9 (q), 17.2 (q), 16.1 (q), 15.4 (q); EI-MS *m/z* 316 $[M]^+$.

5 Sarcophinone (9)

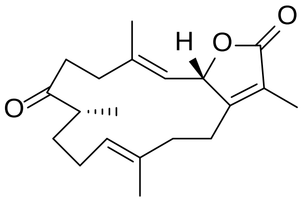

Colorless oil, $[\alpha]_D^{20}$ +5° (*c* 2.2, $CHCl_3$); ^{1}H-NMR ($CDCl_3$, 500 MHz) δ 5.43 (1H, d, *J*=9.5 Hz), 4.95 (1H, d, *J*=9.5 Hz), 4.83 (1H, m), 2.74 (1H, m), 2.35(2H, m), 2.35 (2H, m), 2.18 (1H, m), 2.14 (1H, m), 2.08 (1H, m), 2.04 (1H, m), 1.92 (1H, m), 1.86 (1H, m), 1.82 (3H, s), 1.80 (3H, s), 1.67 (1H, m), 1.56 (3H, s), 1.56 (1H, m), 1.17 (3H, d, *J*=6.5 Hz), 1.08 (1H, m); ^{13}C-NMR ($CDCl_3$, 125 MHz) δ 213.3 (s),175.0 (s), 162.9 (s), 142.0 (s), 134.9 (s), 124.1 (d), 122.2 (d), 122.1 (s), 78.9 (d), 46.7 (d), 37.9 (t), 36.2 (t), 32.8 (t), 32.1 (t), 26.5 (t), 26.1 (t), 18.8 (q), 16.2 (q), 16.0 (q), 9.0 (q); EI-MS *m/z* 316 $[M]^+$.

6 7α-hydroxy-$\Delta^{8(19)}$-deepoxysarcophine (10)

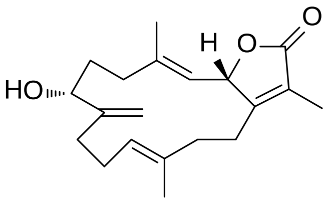

Colorless oil, $[\alpha]^{25.6}_{D}$ +5° (*c* 2.2, $CHCl_3$); ^{1}H-NMR ($CDCl_3$, 500 MHz), δ 5.1 (1H, m), 5.09 (1H, brs), 4.95 (1H, brs), 4.11 (1H, s), 2.71 (1H, m), 2.44 (1H, ddd, *J*=13.8 Hz, 12.8 Hz, 3.8 Hz), 2.34 (1H, m), 2.23 (1H, m), 2.19 (1H, m), 2.18(1H,m), 2.16(1H, m), 2.12 (1H, m), 2.06 (1H, m), 2.02 (1H, m), 1.91 (3H , s), 1.91 (1H, m), 1.86 (3H, s), 1.64 (3H, s), 1.41 (1H, m);^{13}C-NMR ($CDCl_3$, 125 MHz) δ 174.9(s), 162.6 (s), 154.5 (s), 143.6 (s), 134.9 (s), 125.8 (d), 123.0 (s), 121.4 (d), 109.9 (t), 78.7 (d), 70.5 (d), 36.8 (t), 36.0 (t), 33.8 (t), 32.1 (t), 29.9 (t), 26.3 (t), 16.0 (q), 15.4 (q), 8.9 (q); EI-MS m/z 316 $[M]^+$.

7 4β-hydroxy-$\Delta^{2(3)}$-sarcophine (11)

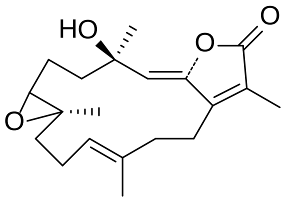

Colorless oil, $[\alpha]^{25}_{D}$ -2° (c 0.7, $CHCl_3$); ^{1}H-NMR ($CDCl_3$, 500 MHz), δ 5.45 (1H, s), 5.01 (1H, t), 2.70 (1H, m), 2.56 (2H, m), 2.45 (1H, m), 2.30 (1H, m), 2.10 (1H, m), 2.09 (1H, m), 2.00 (1H, m), 1.97 (3H, s), 1.95 (1H, m), 1.85 (1H, m), 1.70 (1H, m), 1.65 (3H, s), 1.65 (1H, m), 1.53 (3H, s), 1.26 (3H, s); ^{13}C-NMR ($CDCl_3$, 125 MHz) δ 170.2 (s), 151.9 (s), 147.8 (s), 133.3 (s), 128.1 (d), 123.5 (s), 117.6 (d), 72.6 (s), 61.6 (s), 60.5 (d), 39.6 (t), 37.6 (t), 35.8 (t), 29.6 (q), 23.5 (t), 22.9 (t), 22.7 (t), 18.8 (q), 16.4 (q), 9.0 (q); EI-MS *m/z* 332 $[M]^+$.

8 7α,8β-dihydroxydeepoxysarcophine (12)

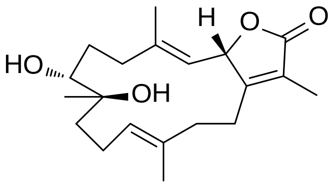

Colorless oil, $[\alpha]^{26}_{D}$ +5° (c 0.01, $CHCl_3$); ^{1}H-NMR ($CDCl_3$, 500 MHz), δ 5.77 (1H, d, *J*=10 Hz), 4.83 (1H, d, *J*=10 Hz), 4.13 (2H, m), 4.01 (1H, m), 3.87 (1H,m), 2.64 (1H, m), 2.20 (1H, m), 2.20 (1H, m), 2.01 (1H, m), 2.00 (1H, m), 1.96 (1H, m), 1.82 (1H, s), 1.80 (3H, s), 1.75 (3H, s), 1.58 (3H, s), 1.35 (1H, m), 0.98 (3H, s); ^{13}C-NMR ($CDCl_3$, 125 MHz) δ 174.7 (s), 164.8 (s), 144.3 (s), 133.5 (s), 125.2(d), 121.2 (d), 121.5 (d), 79.3 (d), 74.1 (s), 71.6 (s), 70.9 (d), 37.4 (t), 36.2 (t), 29.5 (t), 27.7 (d), 26.6 (t), 26.2 (t), 25.0 (q), 19.3 (q), 16.0 (q), 9.0 (q); EI-MS *m/z* 334 $[M]^+$.

9 1,15*β*-epoxy-2-*epi*-16-deoxysarcophine (13)

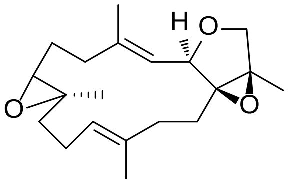

Colorless oil, $[\alpha]^{25.9}_{D}$-25° (*c* 2.2, $CHCl_3$); ^{1}H-NMR ($CDCl_3$, 500 MHz), δ 5.28 (1H, d, *J*=11 Hz), 5.13 (1H, m), 4.88 (1H, d, *J*=11 Hz), 3.92 (1H, d, *J*=10 Hz), 3.38 (1H, d, *J*=10 Hz), 2.67 (1H, dd), 2.35 (2H,

m), 2.34 (1H, m), 2.15 (2H, m),1.95 (1H, m), 1.70 (2H, m), 1.62 (2H, m), 1.45 (3H, s), 1.29 (2H, d); ^{13}C-NMR ($CDCl_3$, 125 MHz) δ 140.9 (s), 136.2 (s), 123.9 (d), 122.4 (d), 76.4 (d), 72.0 (s), 68.0 (s), 67.9 (t), 61.9 (d), 59.8 (s), 38.9 (t), 36.3 (t), 34.8 (t), 29.0 (t), 25.8 (t), 24.3 (t), 12.1 (q); EI-MS *m/z* 322 $[M]^+$.

10 Sarcophytol Q (14)

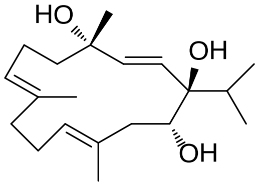

Colorless oil, $[\alpha]_D^{32.5}$ -87° (*c* 2.2, $CDCl_3$); ^{1}H-NMR ($CDCl_3$, 500 MHz), δ 6.09 (1H, d, *J*=16.0Hz), 5.81 (1H, d, *J*=16.0, 5.0Hz), 5.36 (1H, t), 5.21 (1H, d), 4.08 (1H, dd), 2.38 (1H, m), 2.25 (1H, m), 2.01 (1H, m), 2.00 (2H, m), 1.99 (1H, m), 1.90 (1H, m), 1.88 (3H, s), 1.62 (3H, s), 1.55 (1H, m), 1.43 (3H, s), 1.06 (3H, d), 0.87 (3H, d); ^{13}C-NMR ($CDCl_3$, 125 MHz) δ 136.8 (d), 132.9 (s), 128.8 (d), 128.5 (d), 128.1 (d), 79.8 (s), 72.4 (s), 70.2 (d), 46.0 (t), 43.7 (t), 38.9 (t), 32.4 (d), 32.4 (d), 22.4 (t), 17.9 (q), 16.8 (q), 15.8 (q), 14.8 (q); EI-MS *m/z* 322 $[M]^+$.

11 Lobocrasol (15)

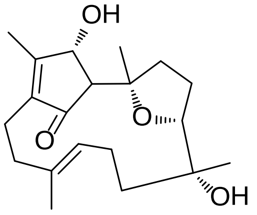

Colorless oil, $[\alpha]_D^{20}$ -180° (c 4.2, $CHCl_3$); IR(KBr)υ_{max}: 3493, 2961, 2926, 2868, 1686, 1642, 1444, 1379, 1051, 1026, 910 cm^{-1}; ^{1}H NMR ($CDCl_3$, 500 MHz) δ 4.70 (1H, s, H-2), 2.00 (1H, s, H-3), 2.85-2.80 (2H, m, H_2-5), 1.94-1.90 (2H, m, H_2-6), 3.65 (1H, dd, *J* = 5.4, 9.2 Hz), 2.40 (1H, m, H-9a), 2.33 (1H, m, H-9b), 1.95 (1H, m, H-10a), 1.79 (1H, m, H-10b), 5.00 (1H, dd, *J* = 5.4, 9.2 Hz, H-11), 2.13 (1H, m, H-13a), 1.84 (1H, m, H-13b), 2.36 (1H, dt, *J* = 11.1, 12.7, H-14a), 1.79 (1H, m, H-14b), 1.99 (3H, s, H-17), 1.25 (3H, s, H-18), 0.87 (3H, s, H-19), 1.54 (3H, s, H-20); ^{13}C NMR ($CDCl_3$, 125 MHz) δ 168.9 (C-1), 73.4 (C-2), 52.9 (C-3), 83.4 (C-4), 32.4 (C-5), 25.8 (C-6), 83.9 (C-7), 72.7 (C-8), 34.9 (C-9), 21.9 (C-10), 129.3 (C-11), 129.3 (C-12), 38.7 (C-13), 19.9 (C-14), 140.4 (C-15), 205.1 (C-16), 13.6 (C-17), 25.5 (C-18), 25.8 (C-19), 15.7 (C-20); HR-ESI-MS *m/z* 357.2036 (calc. for $C_{20}H_{30}O_4Na$, 357.2036).

Figure S1. ^{1}H NMR data of sarcophyolide B (**1**).

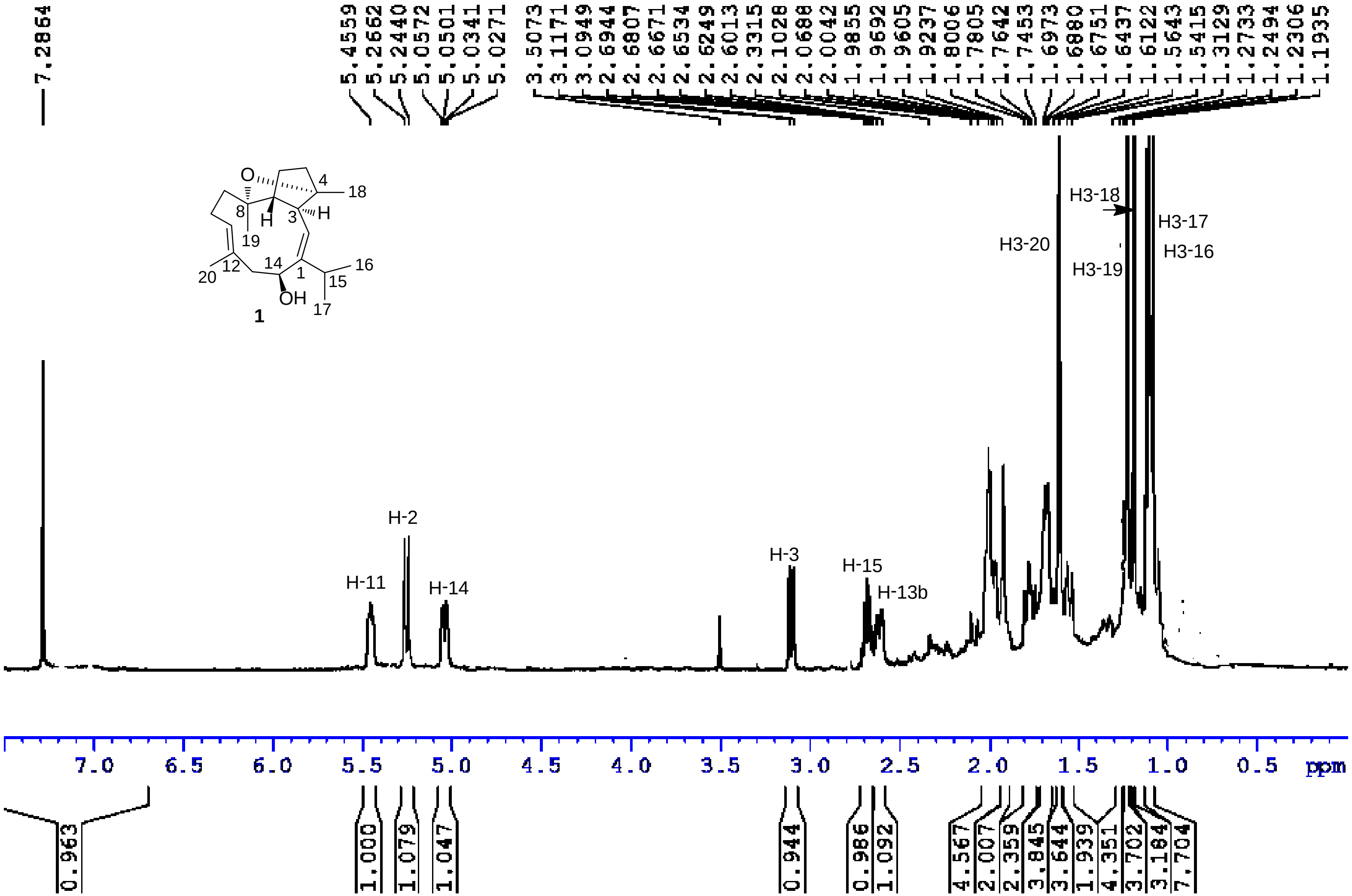

Figure S2. ^{13}C NMR data of sarcophyolide B (**1**).

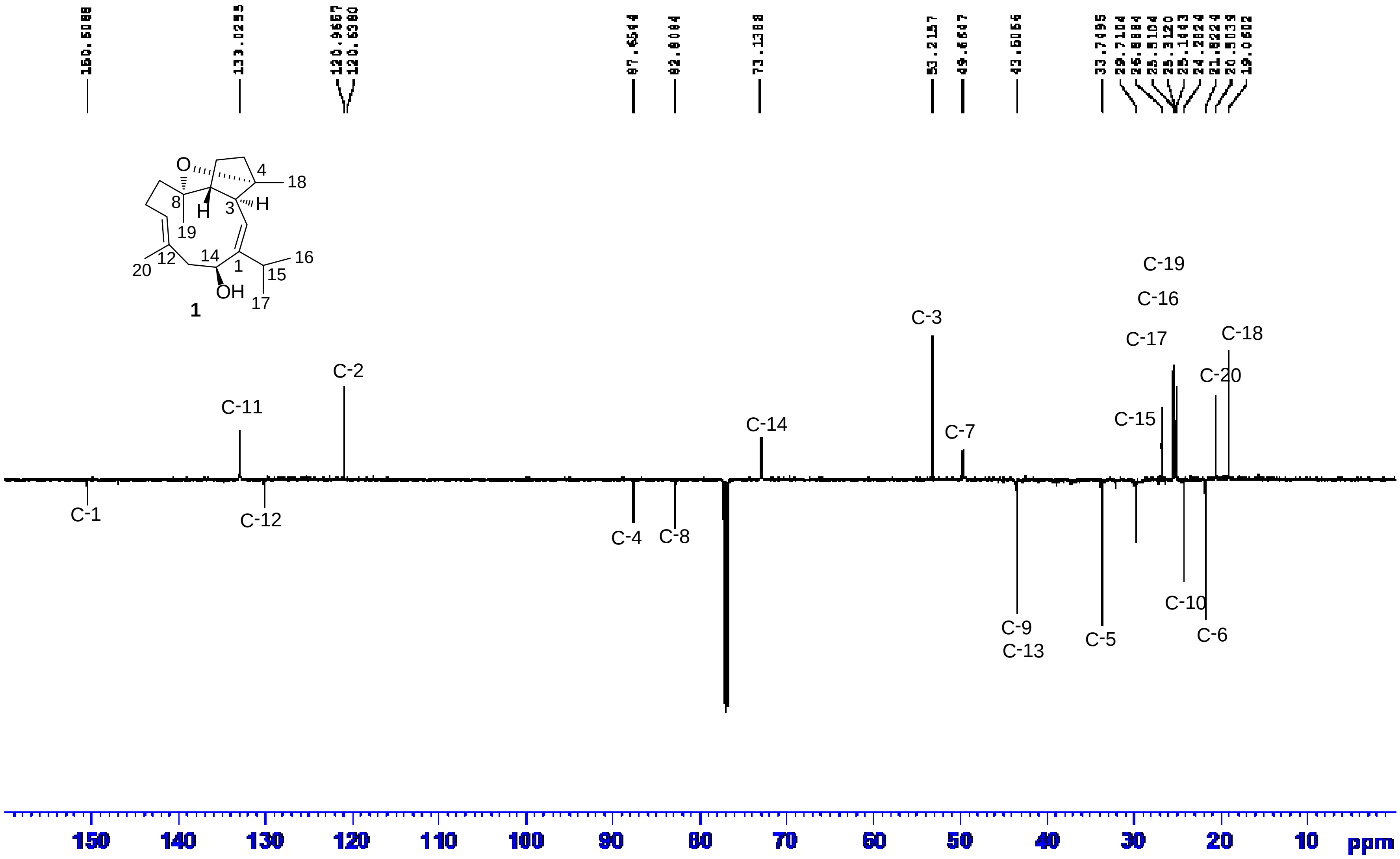

Figure S3. COSY spectrum of sarcophyolide B (**1**).

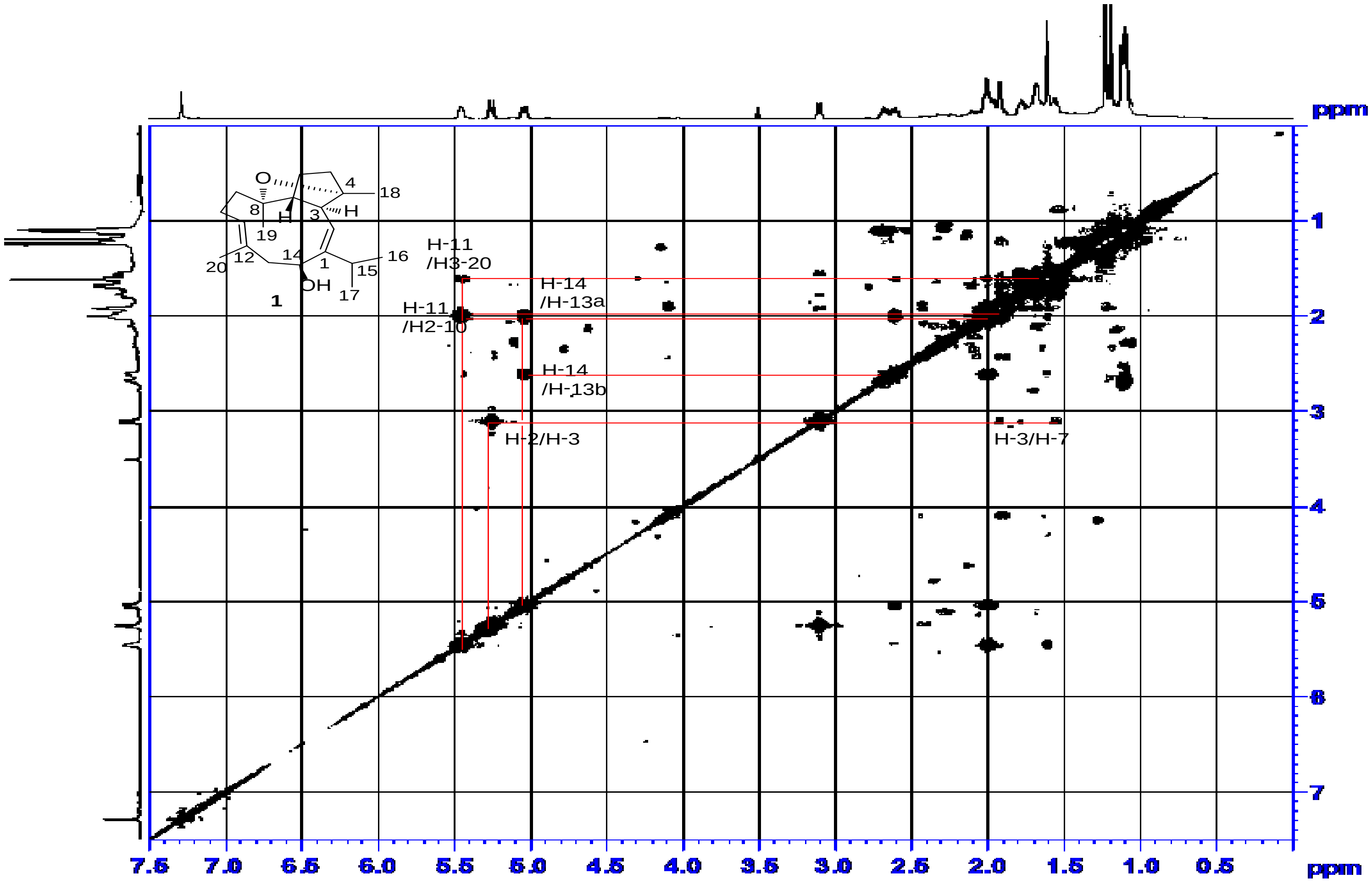

Figure S4. HMQC spectrum of sarcophyolide B (**1**).

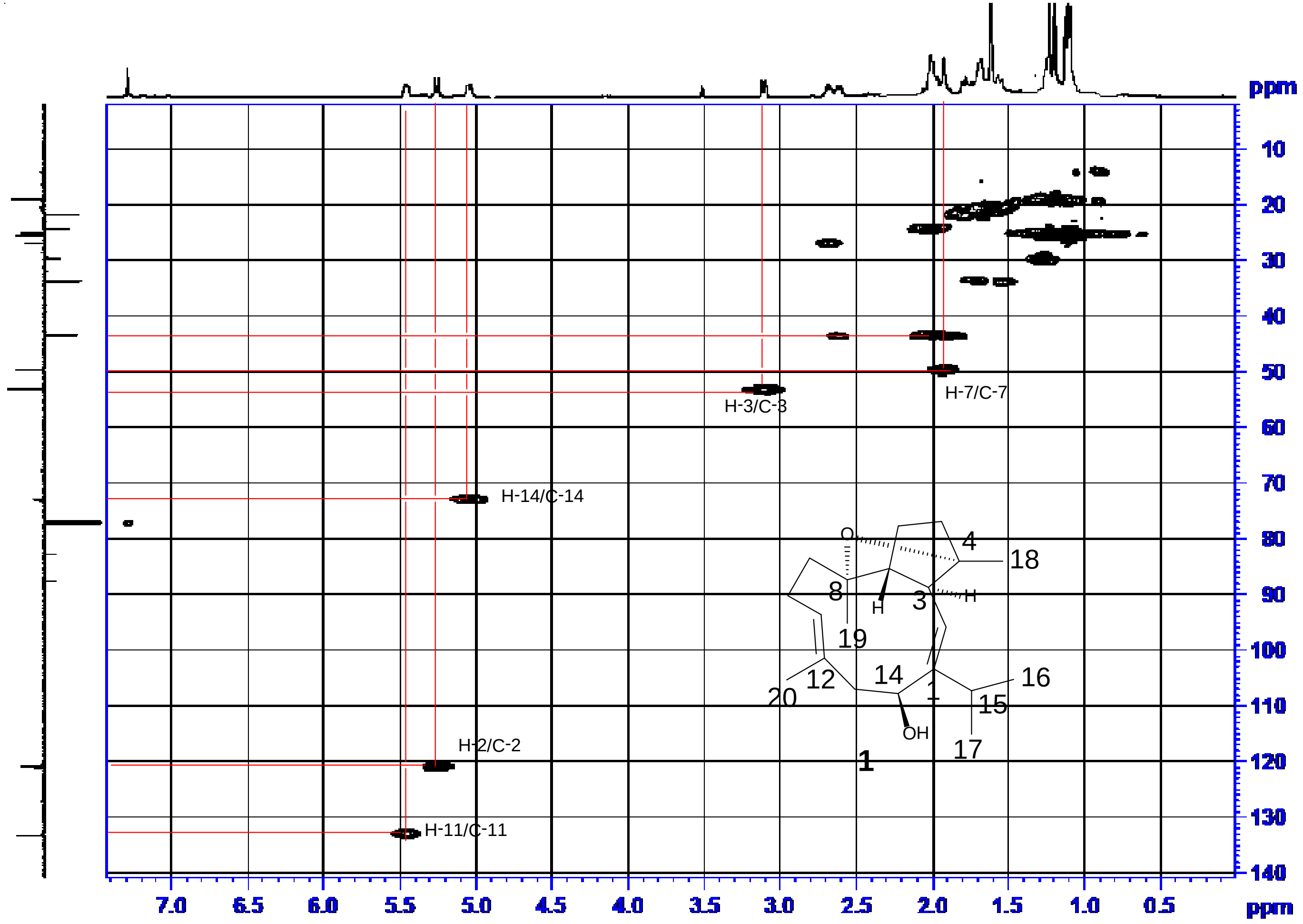

Figure S5. HMBC spectrum of sarcophyolide B (**1**).

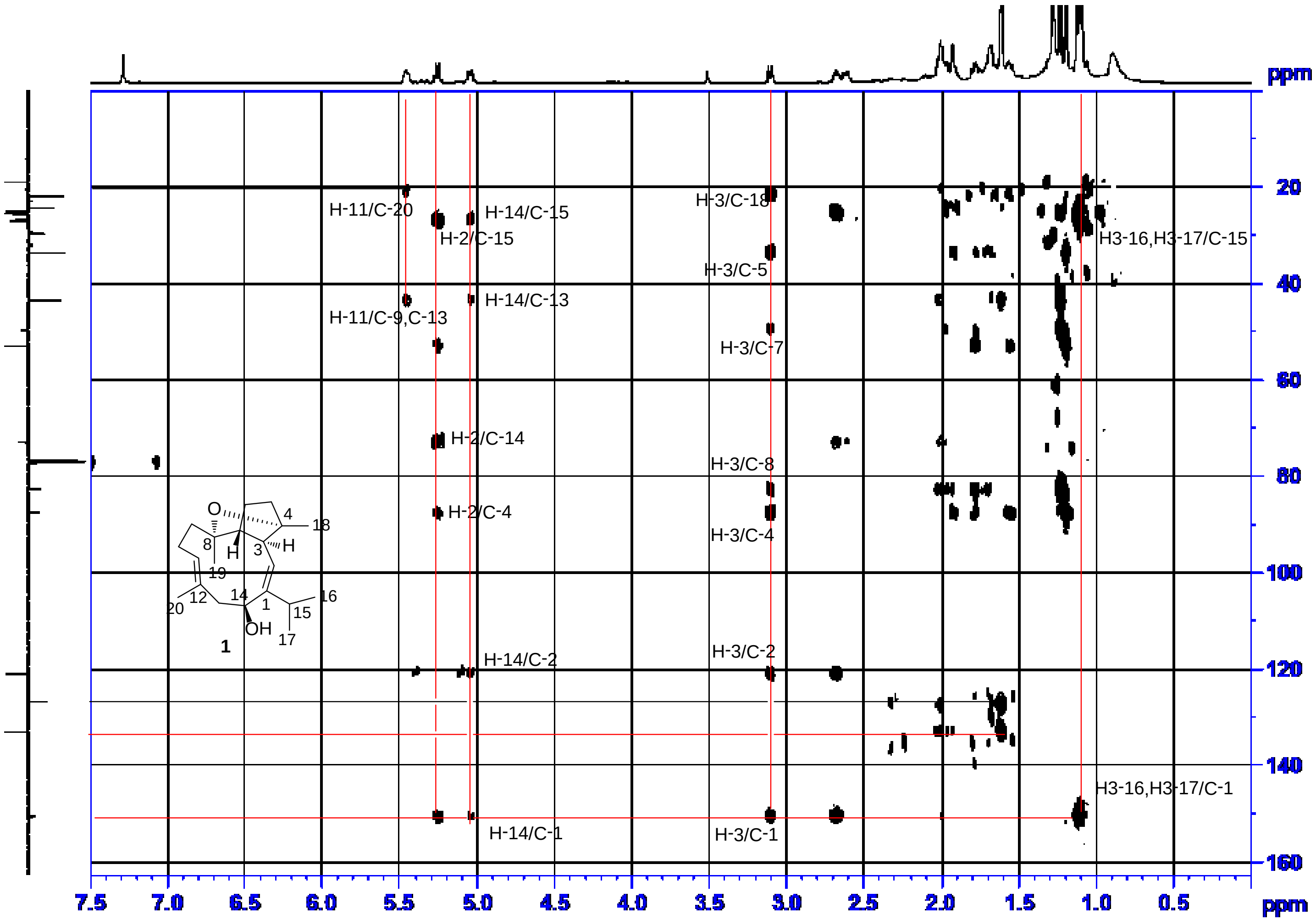

Figure S6. NOESY spectrum of sarcophyolide B (**1**).

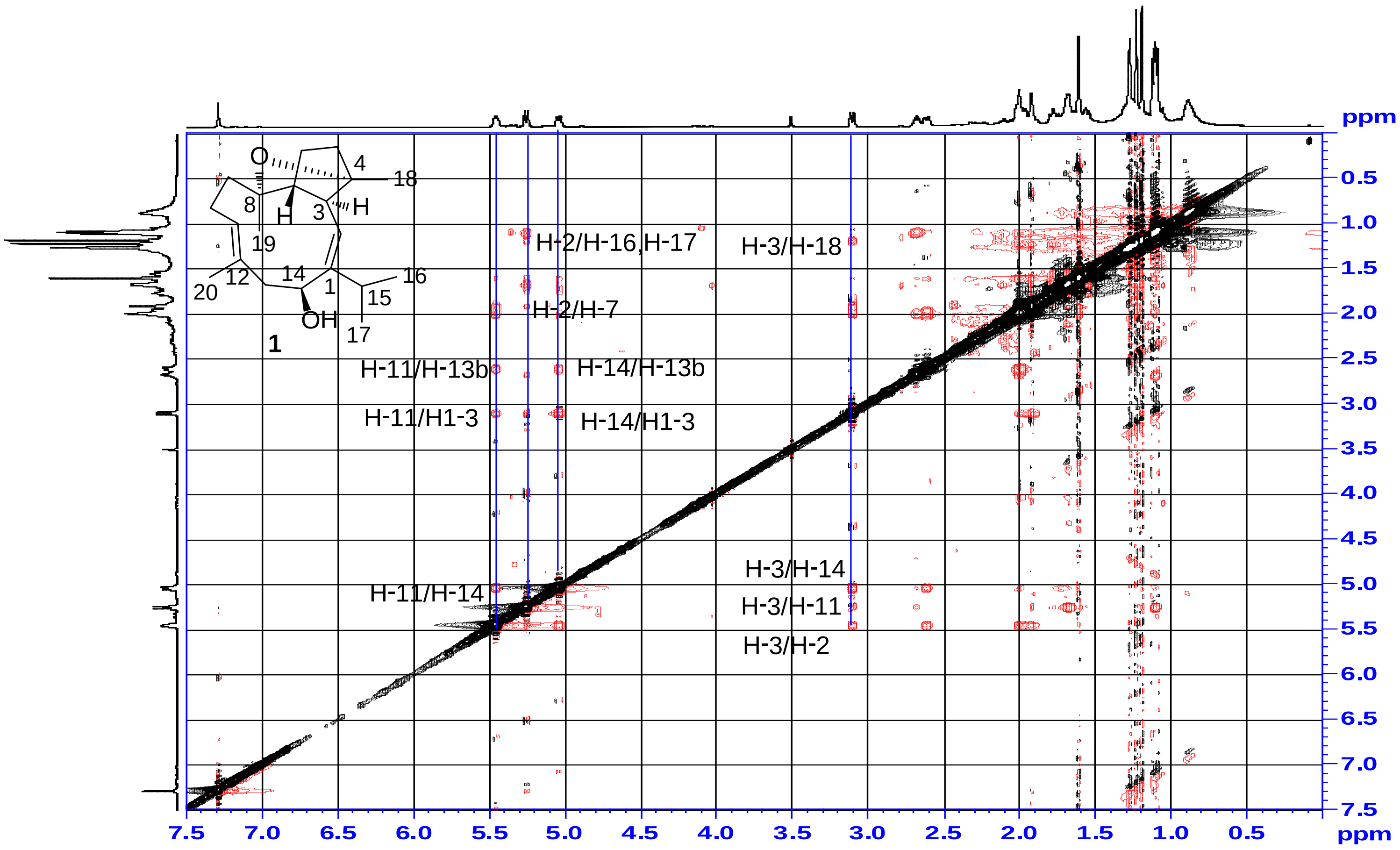

Figure S7. IR spectrum of sarcophyolide B (**1**).

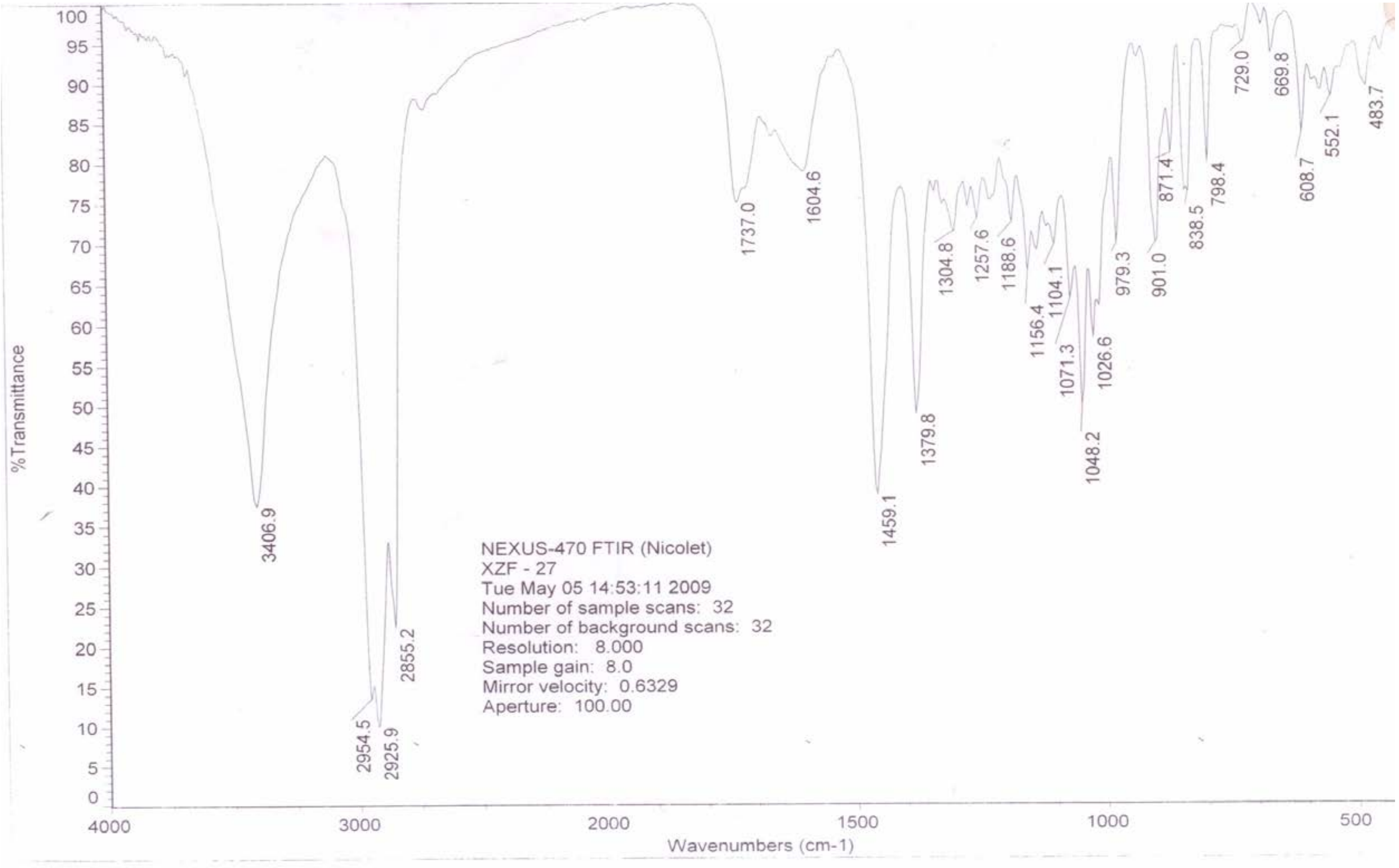

Figure S8. HRMS spectrum of sarcophyolide B (**1**).

BJMU - BRUKER APEX IV FT-MS (7.0T) Spectrum Report

Analysis Info 北京大学医药卫生分析中心
Analysis Name C:\data_sample\ESI_090110_20\ESI_XZF_27_20090115\6

Acquisition Date 1/15/2009 3:34:41 PM
Operator bpfxsh@bjmu.edu.cn; Tel:010-82801437
Instrument FT_MS _Bruker APEX IV (7.0 T)

Comment ESI POS C20H32O2 MW 304.2402
CAL588; 226.16718;249.15695;340.25887;301.14158;
362.24081;391.28483;413.26647;453.34353;
475.32548;509.25407;509.25407;566.42760;
588.40954;679.51166;701.49361;826.47121;

Acquisition Result:	Exact Mass	Measured Mass	Error (mDa)	Error (ppm)	Description
	305.24751 □□□□□	305.2473□□□□□	0.21□□□□□	0.68□□□□□	M+H;-e
	327.22945 □□□□□	327.2292□□□□□	0.25□□□□□	0.77□□□□□	M +Na ;-e

Table 'GenFormulaResults' could not be found in this analysis

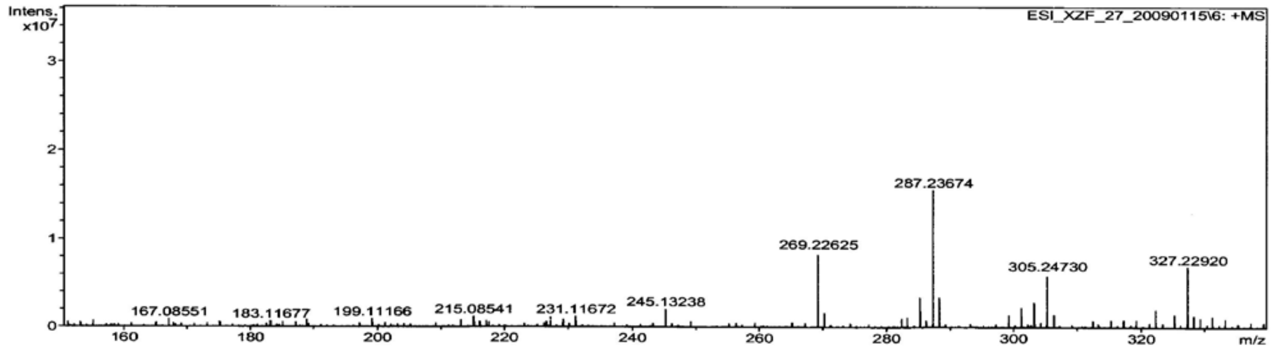

Figure S9. ^{1}H NMR data of sarcophyolide C (**2**).

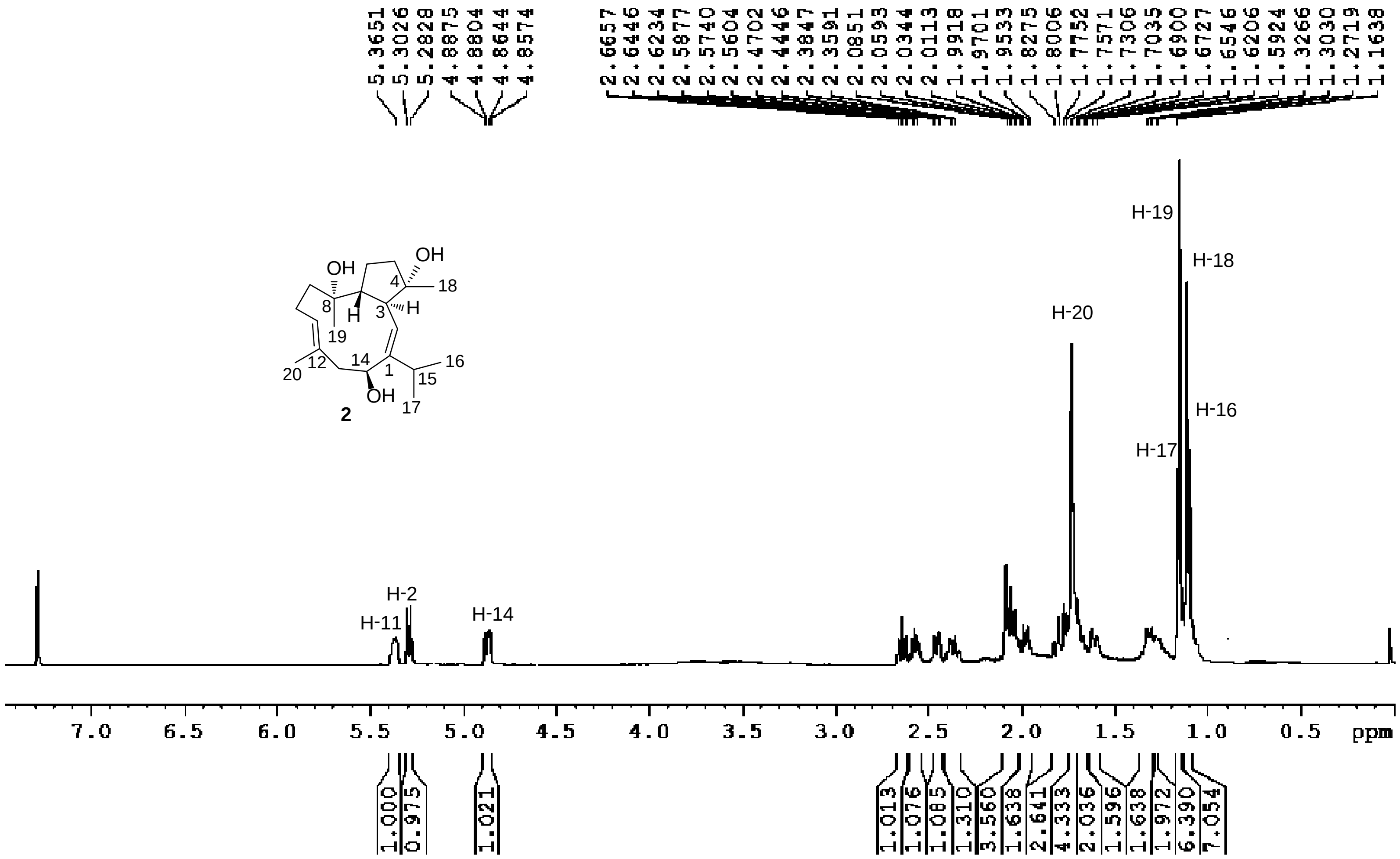

Figure S10. ^{13}C NMR data of sarcophyolide C (**2**).

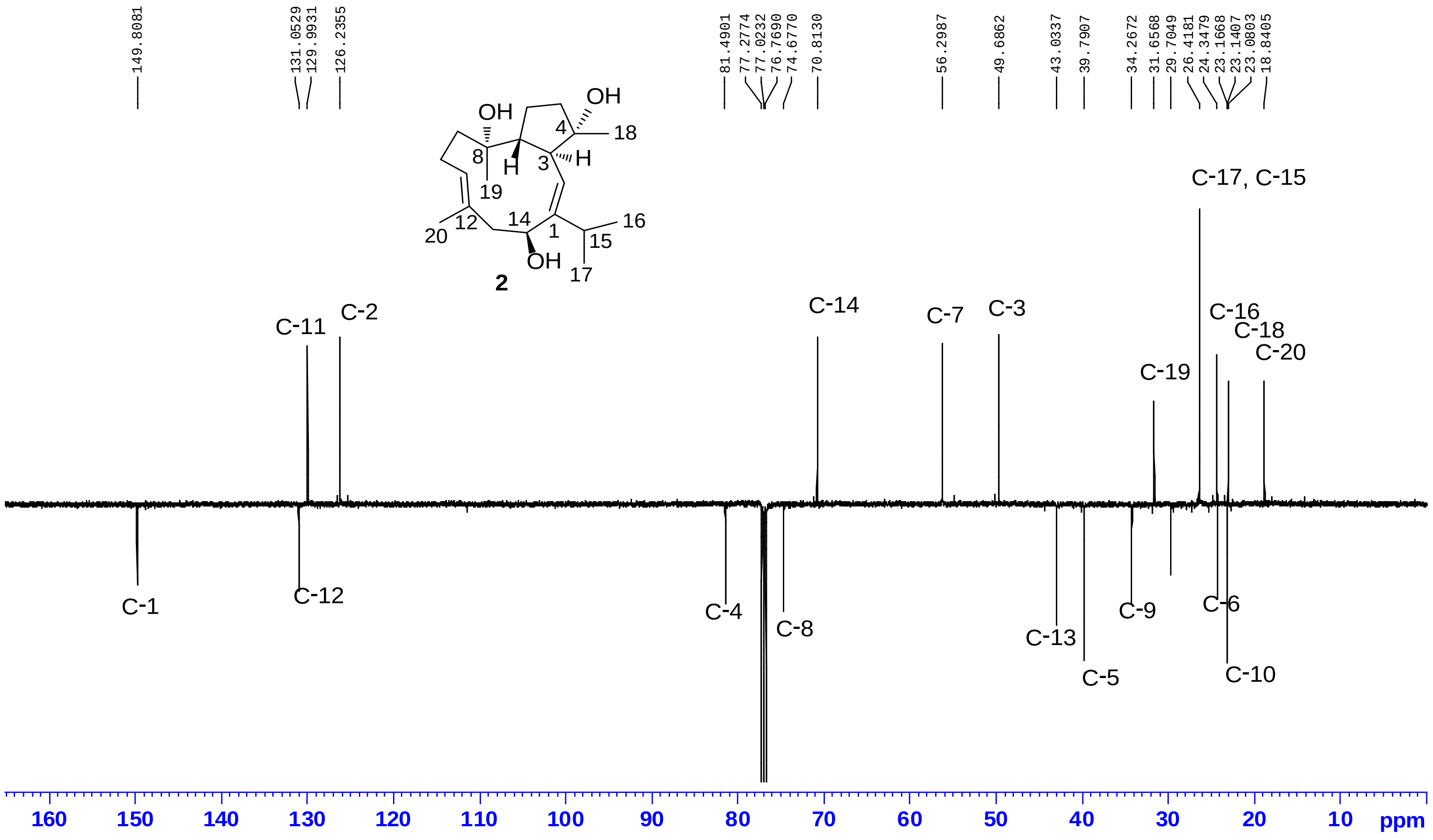

Figure S11. COSY spectrum of sarcophyolide C (**2**).

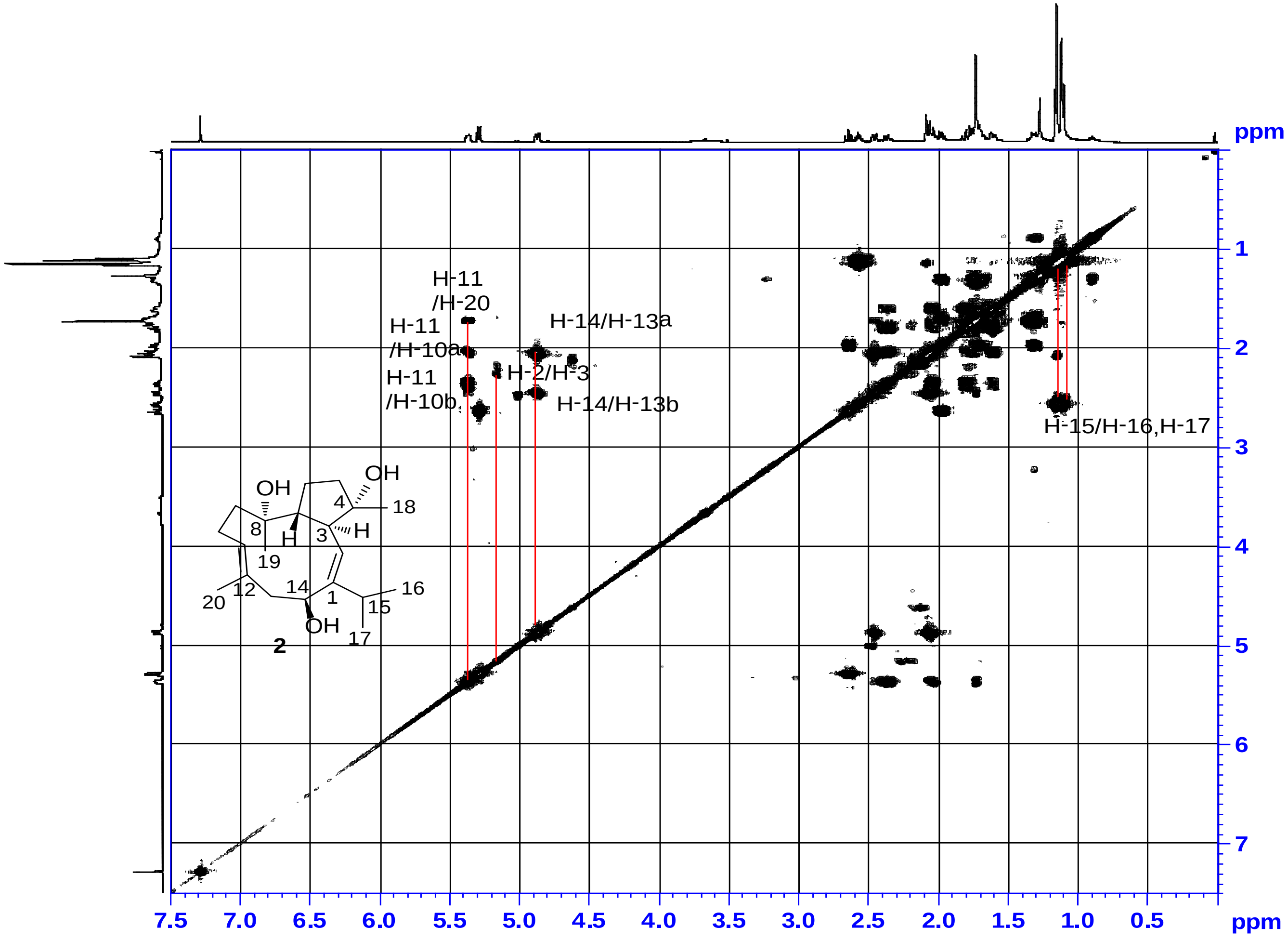

Figure S12. HMQC spectrum of sarcophyolide C (**2**).

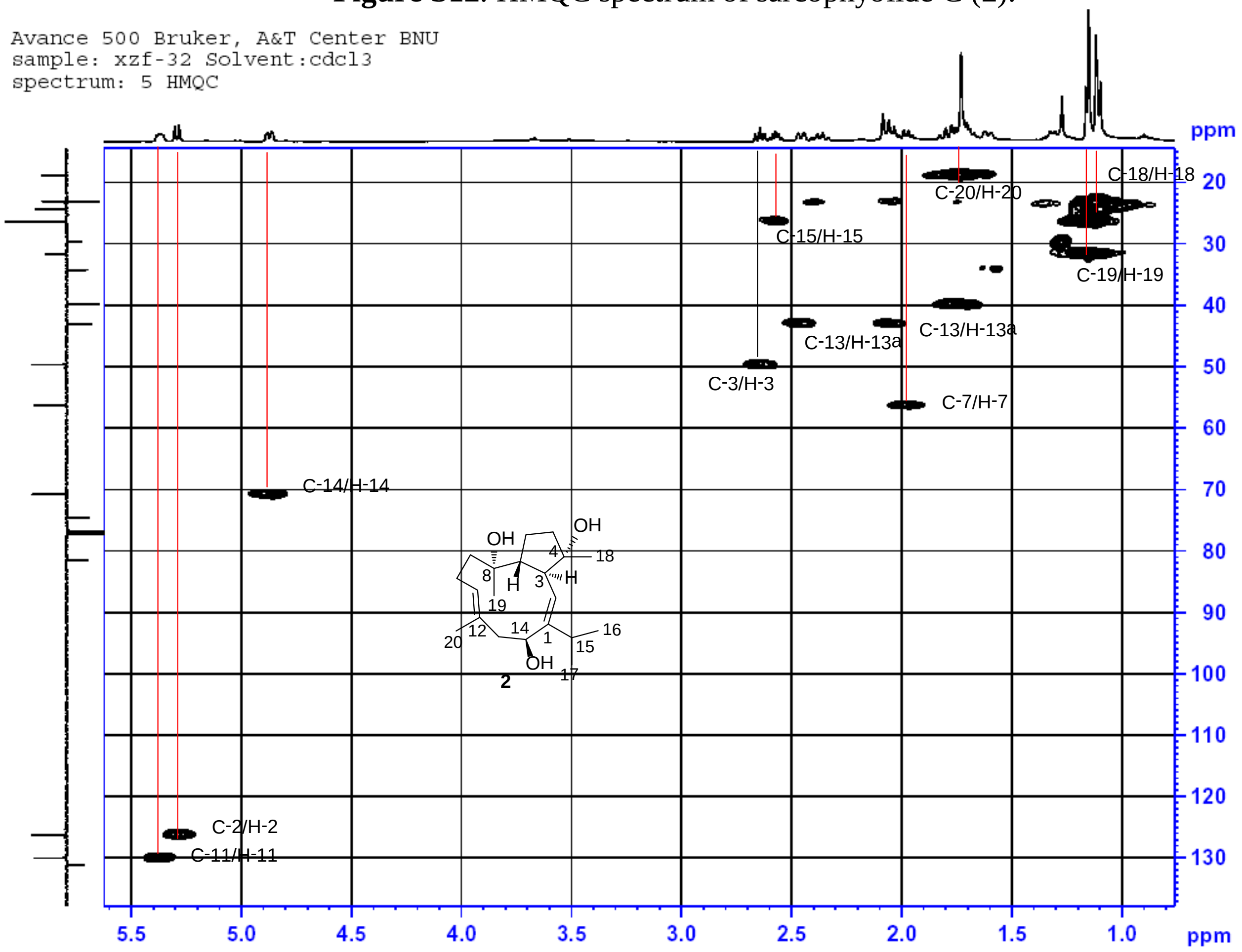

Figure S13. HMBC spectrum of sarcophyolide C (**2**).

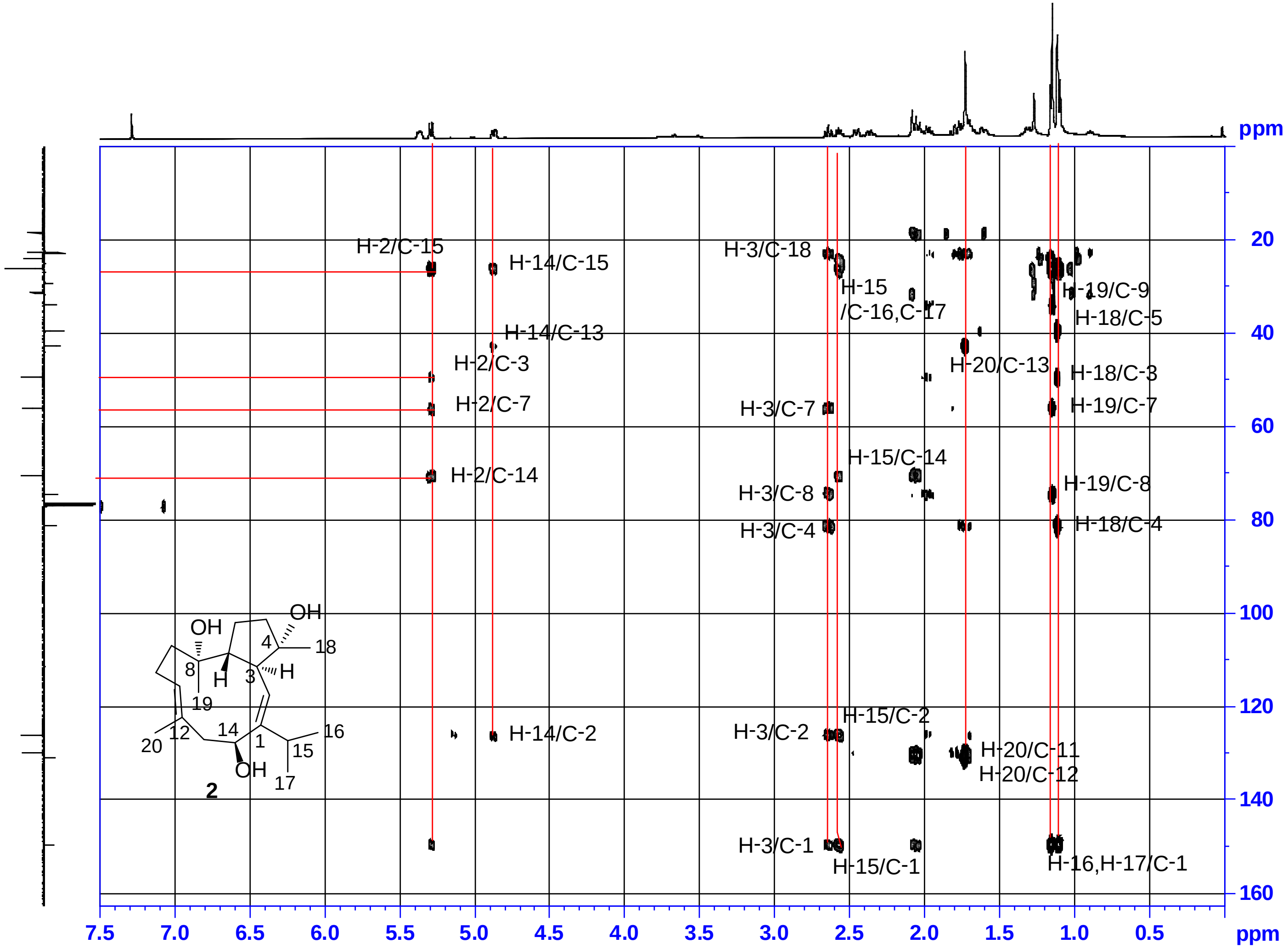

Figure S14. NOESY spectrum of sarcophyolide C (**2**).

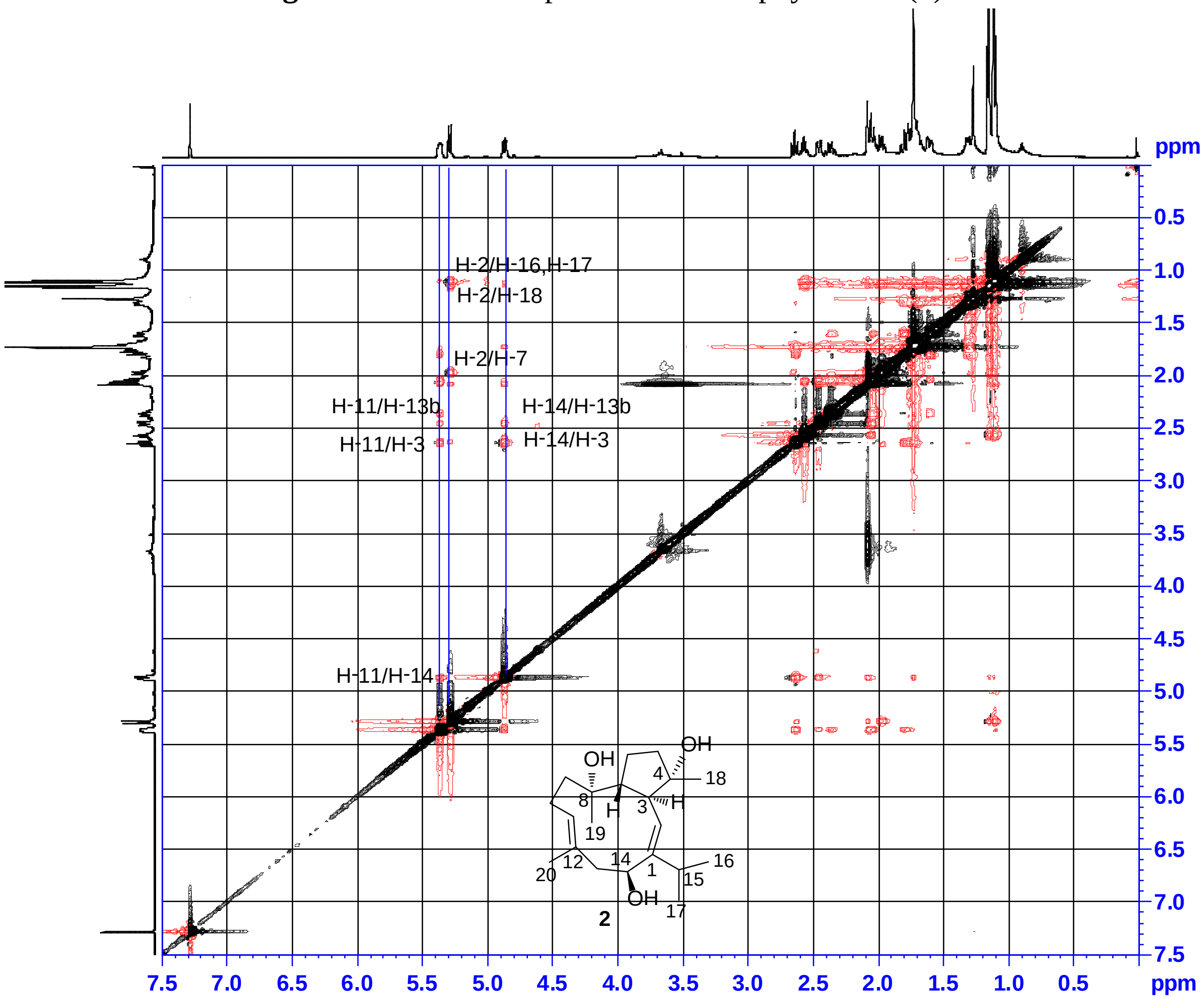

Figure S15. IR spectrum of sarcophyolide C (**2**).

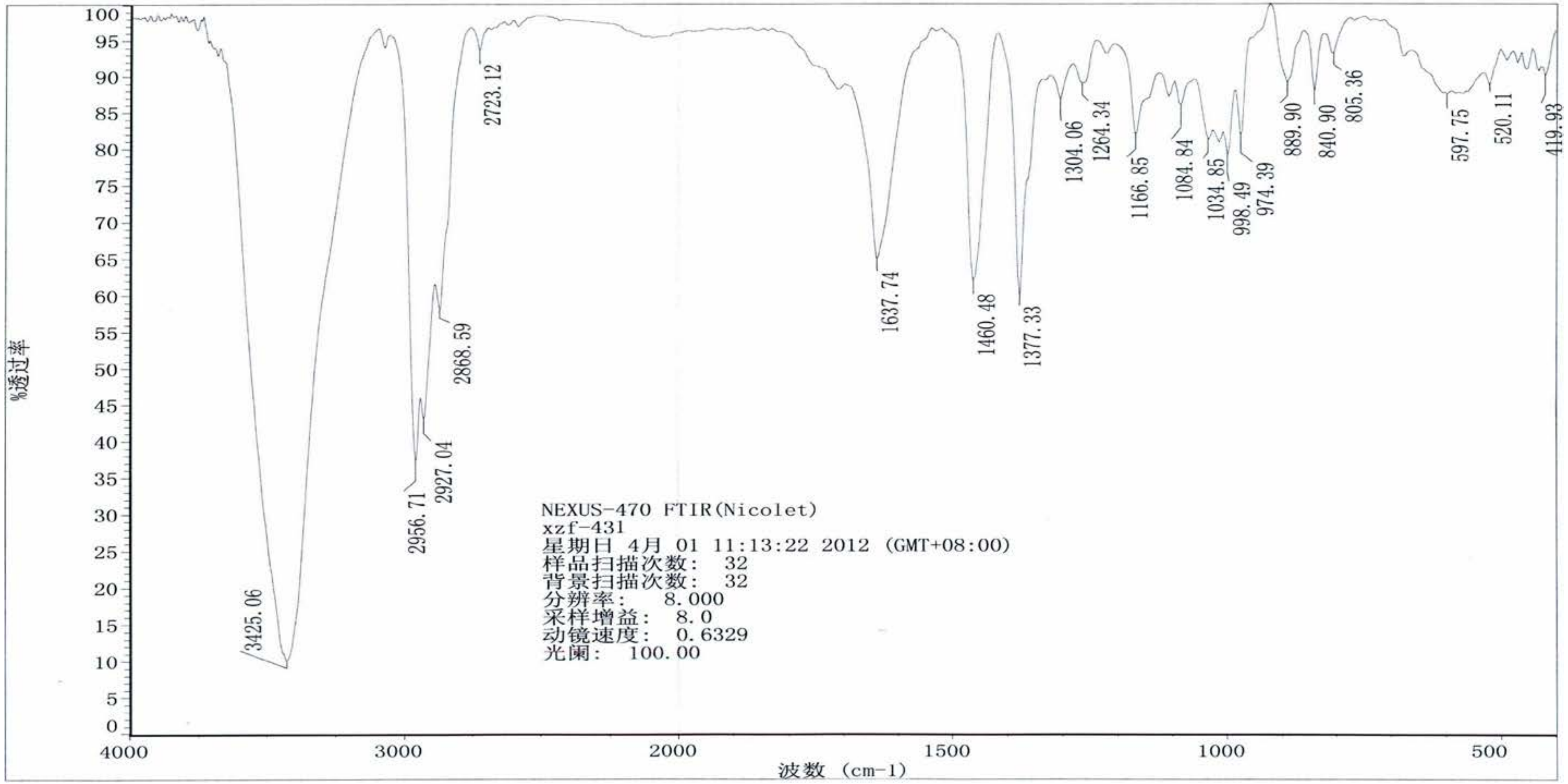

Figure S16. HRMS spectrum of sarcophyolide C (**2**).

BJMU - BRUKER APEX IV FT-MS (7.0T) Spectrum Report

**Analysis Info**	北京大学医药卫生分析中心	**Acquisition Date**	1/15/2009 3:26:47 PM
**Analysis Name**	C:\data_sample\ESI_090110_20\ESI_XZF_32_20090115\5	**Operator**	bpfxsh@bjmu.edu.cn; Tel:010-82801437
		**Instrument**	FT_MS _Bruker APEX IV (7.0 T)

Comment ESI POS C20H34O3 MW 322.2508
CAL588; 226.16718;249.15695;340.25887;301.14158;
362.24081;391.28483;413.26647;453.34353;
475.32548;509.25407;509.25407;566.42760;
588.40954;679.51166;701.49361;826.47121;

**Acquisition Result:**	Exact Mass	Measured Mass	Error (mDa)	Error (ppm)	Description
	**345.24002** □□□□□	**345.24066**□□□□□	**-0.64**□□□□□	**-1.87**□□□□□	**M +Na ;-e**

Table 'GenFormulaResults' could not be found in this analysis

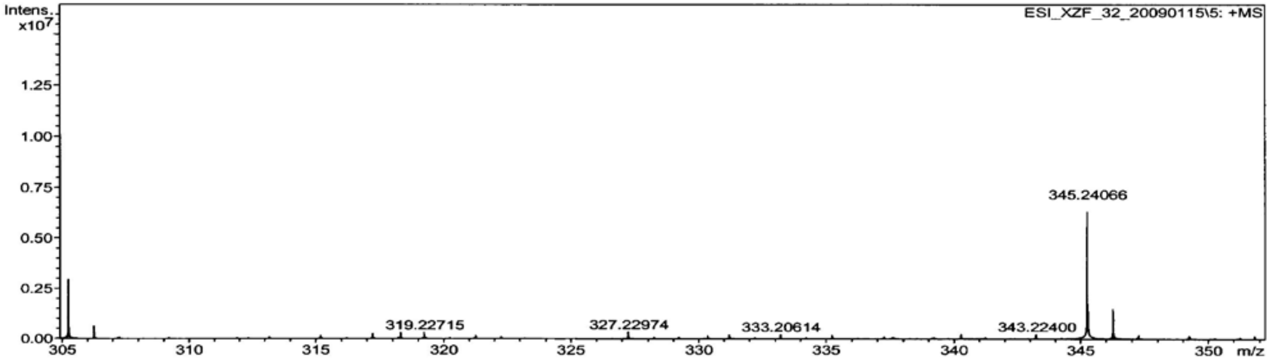

Figure S17. ^{1}H NMR data of sarcophyolide D (**3**).

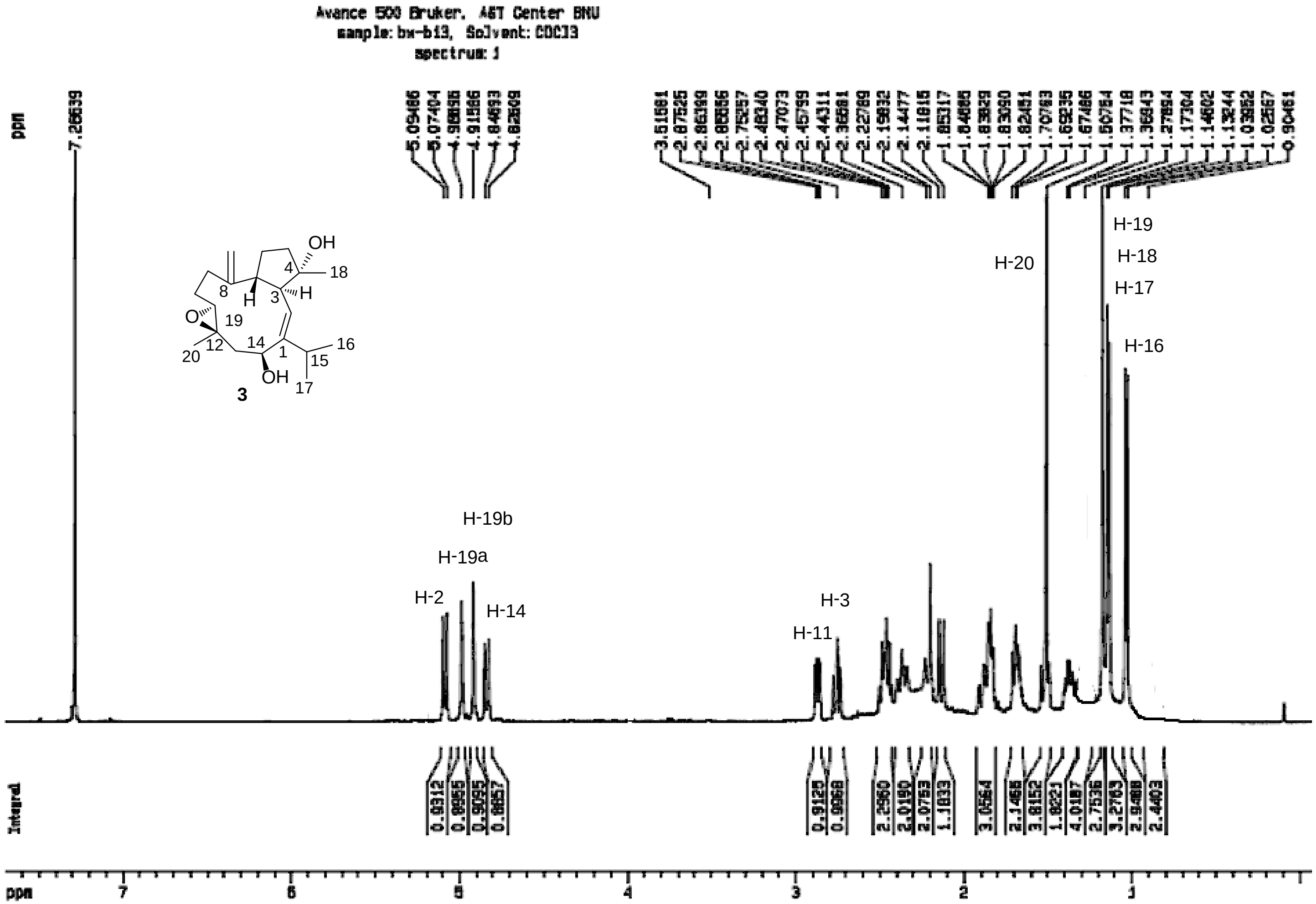

Figure S18. ^{13}C NMR data of sarcophyolide D (**3**).

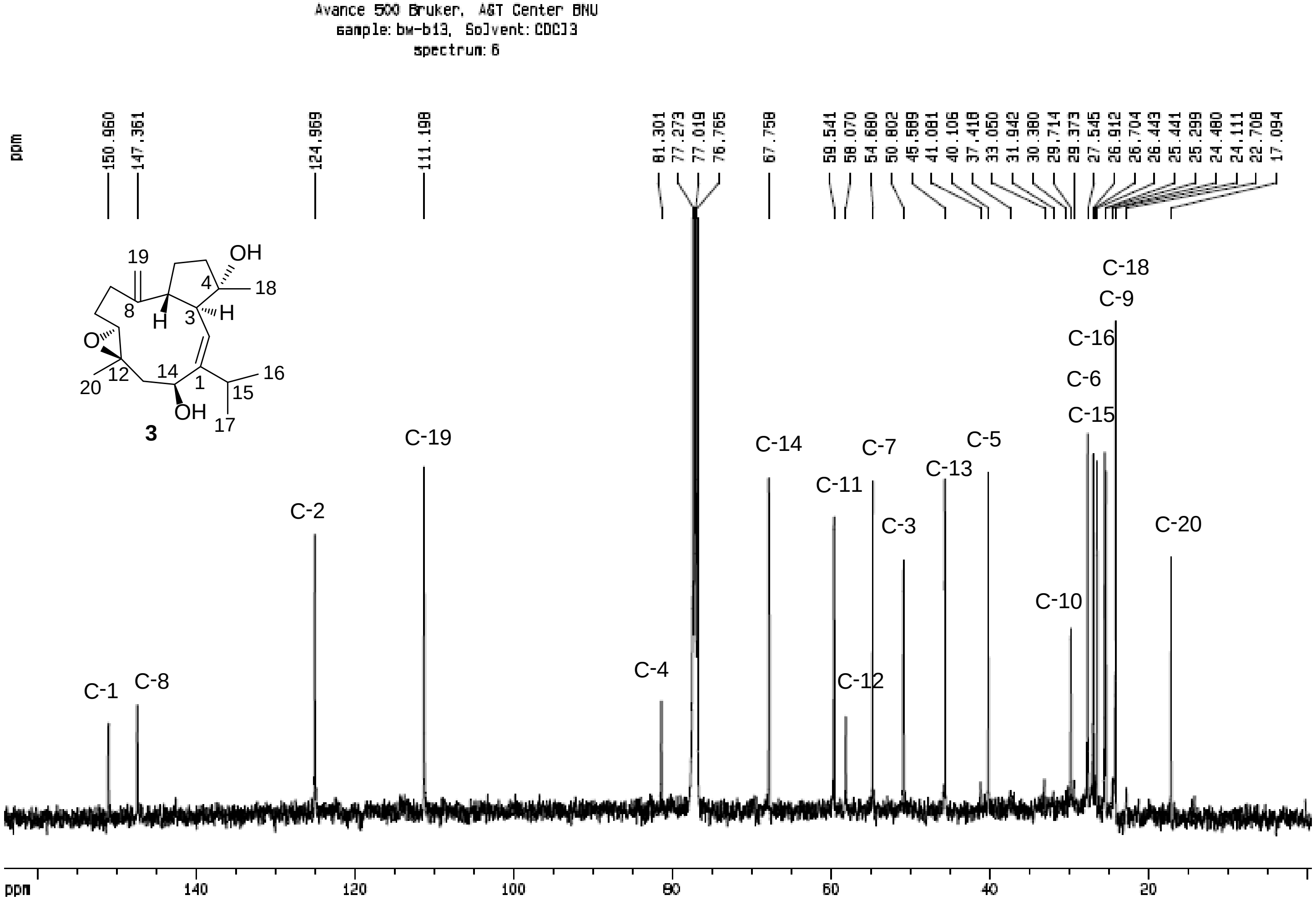

Figure S19. COSY spectrum of sarcophyolide D (**3**).

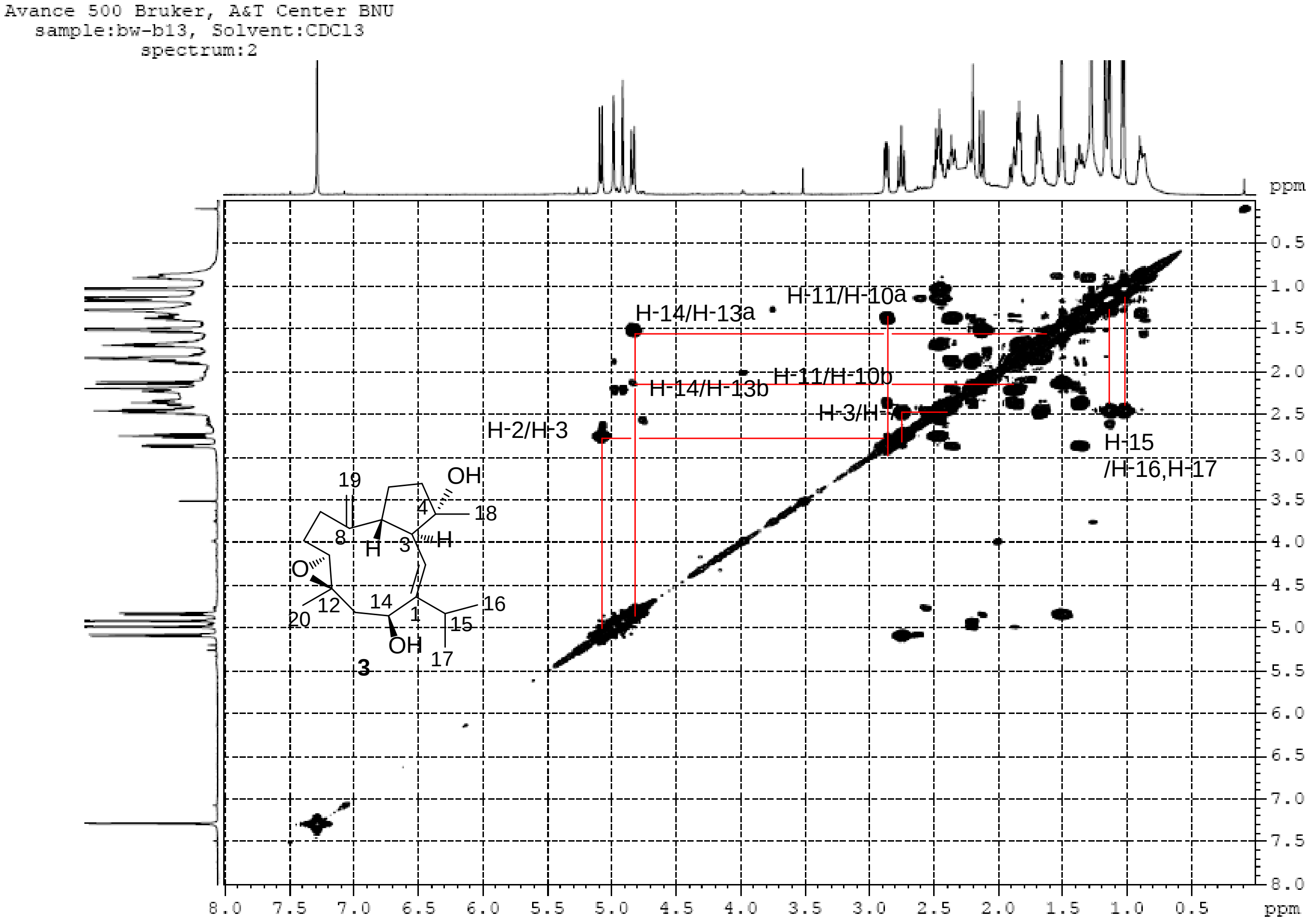

Figure S20. HMQC spectrum of sarcophyolide D (**3**).

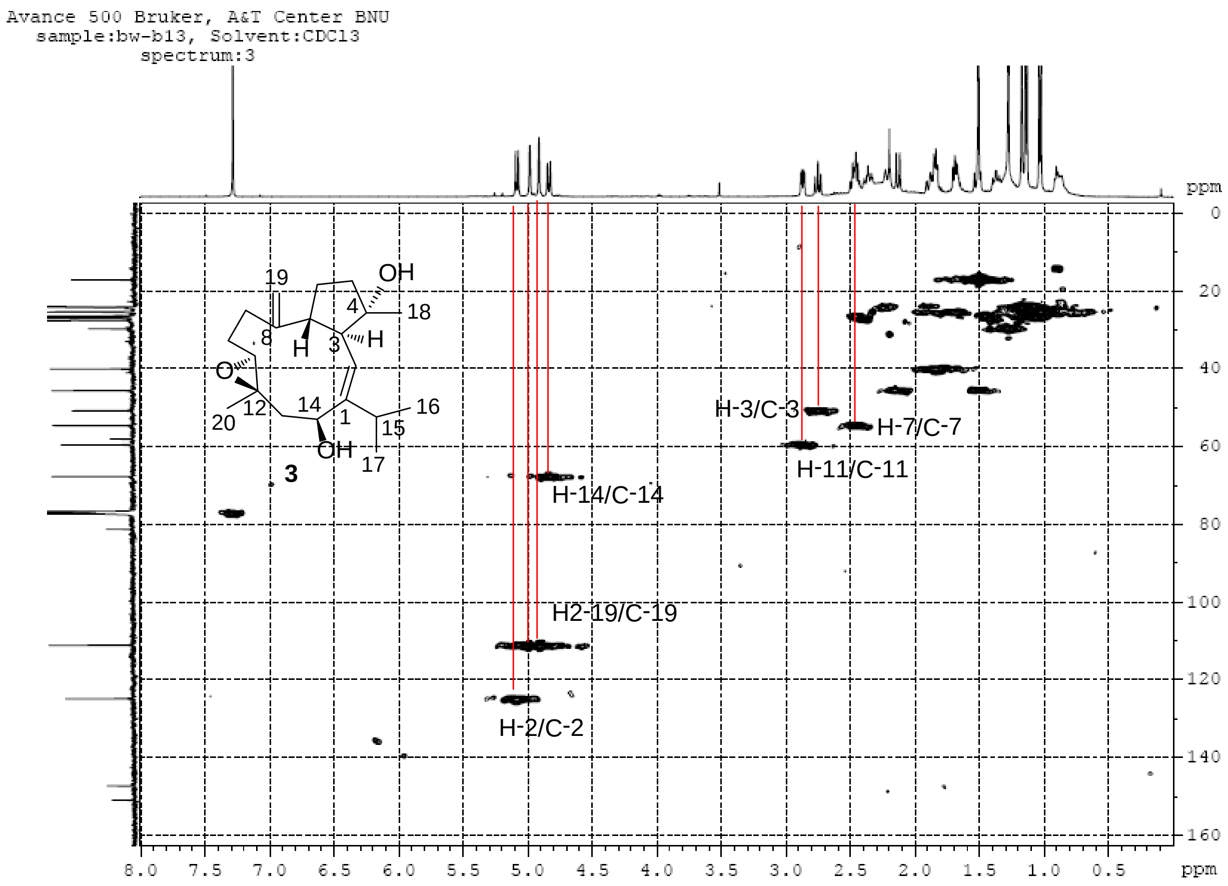

Figure S21. HMBC spectrum of sarcophyolide D (**3**).

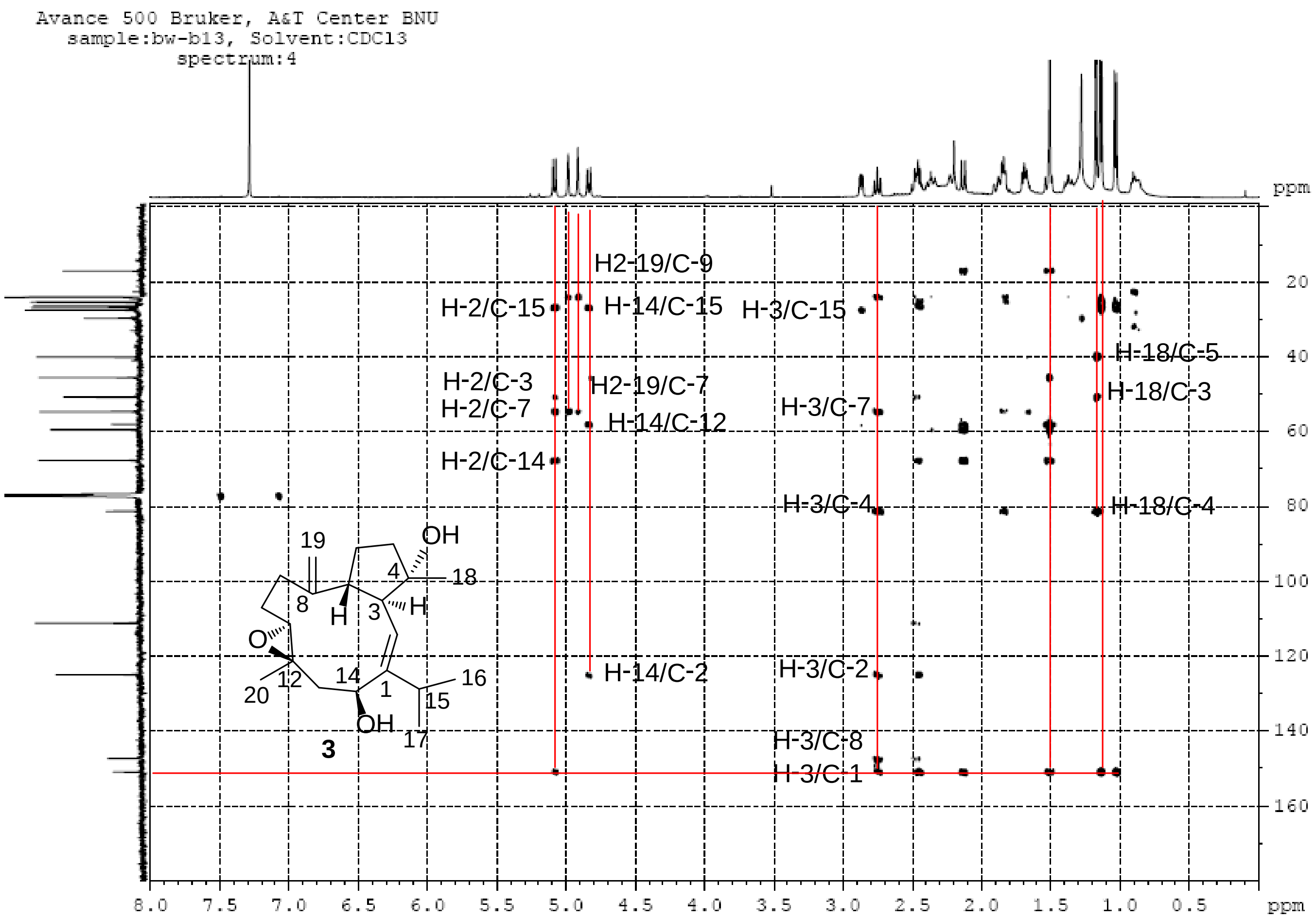

Figure S22. NOESY spectrum of sarcophyolide D (**3**).

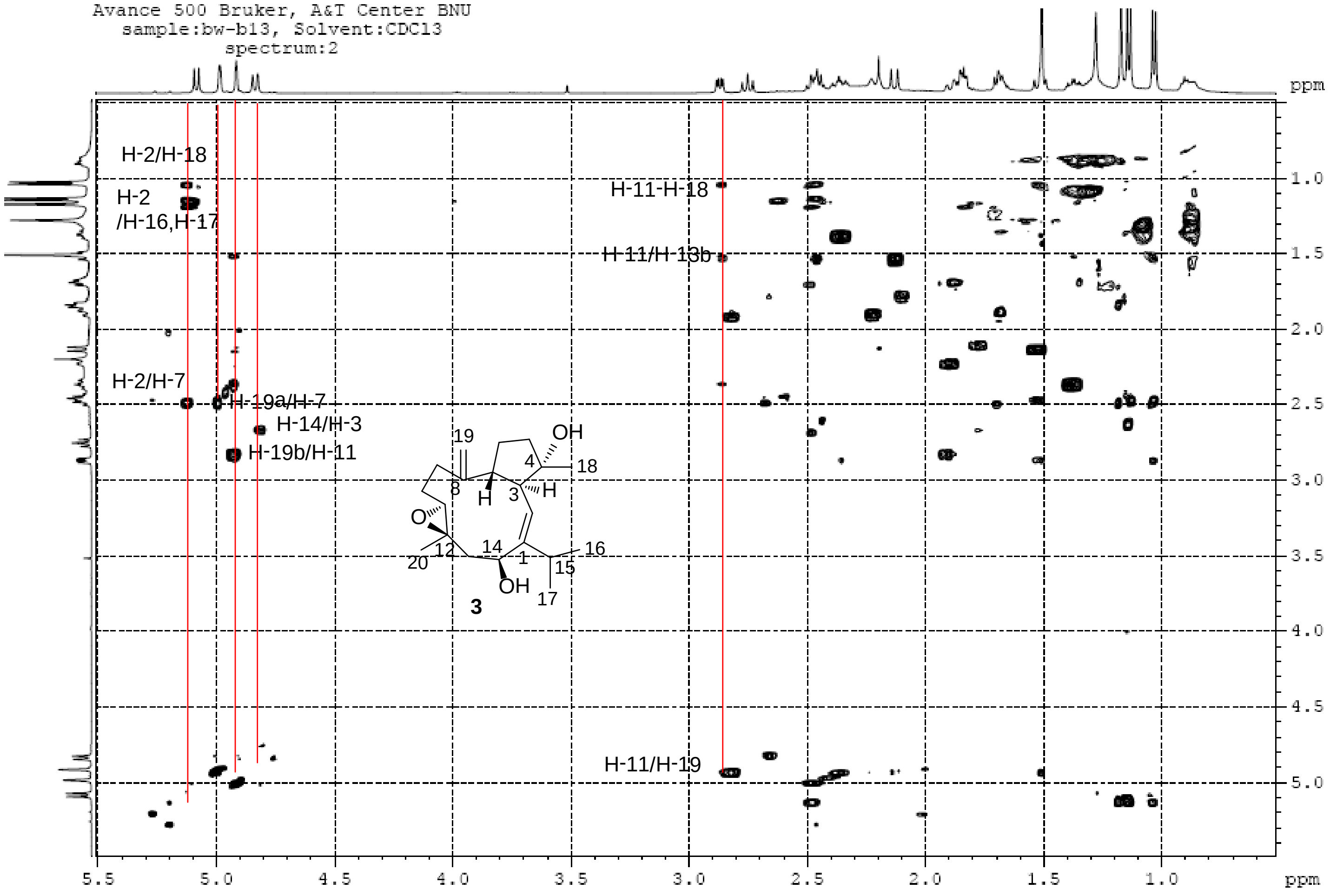

Figure S23. IR spectrum of sarcophyolide D (**3**).

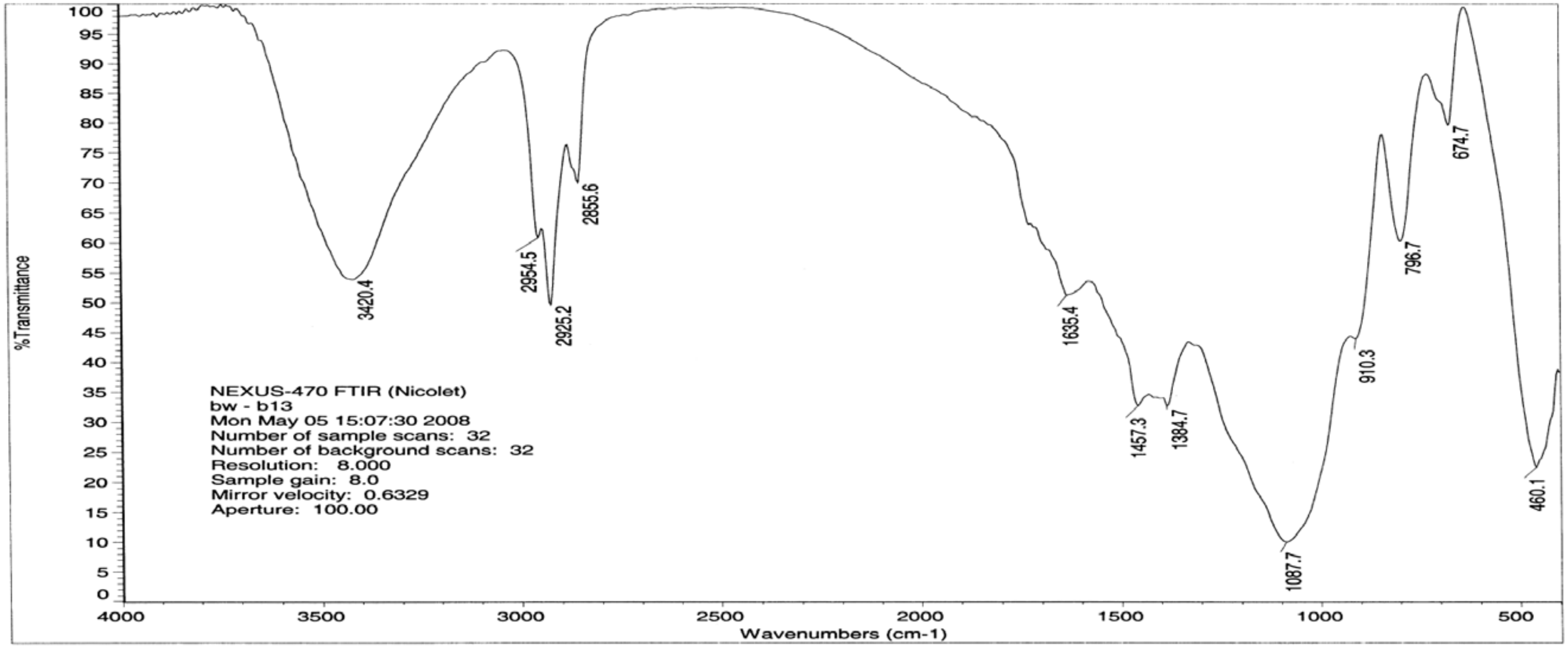

Figure S24. HRMS spectrum of sarcophyolide D (**3**).

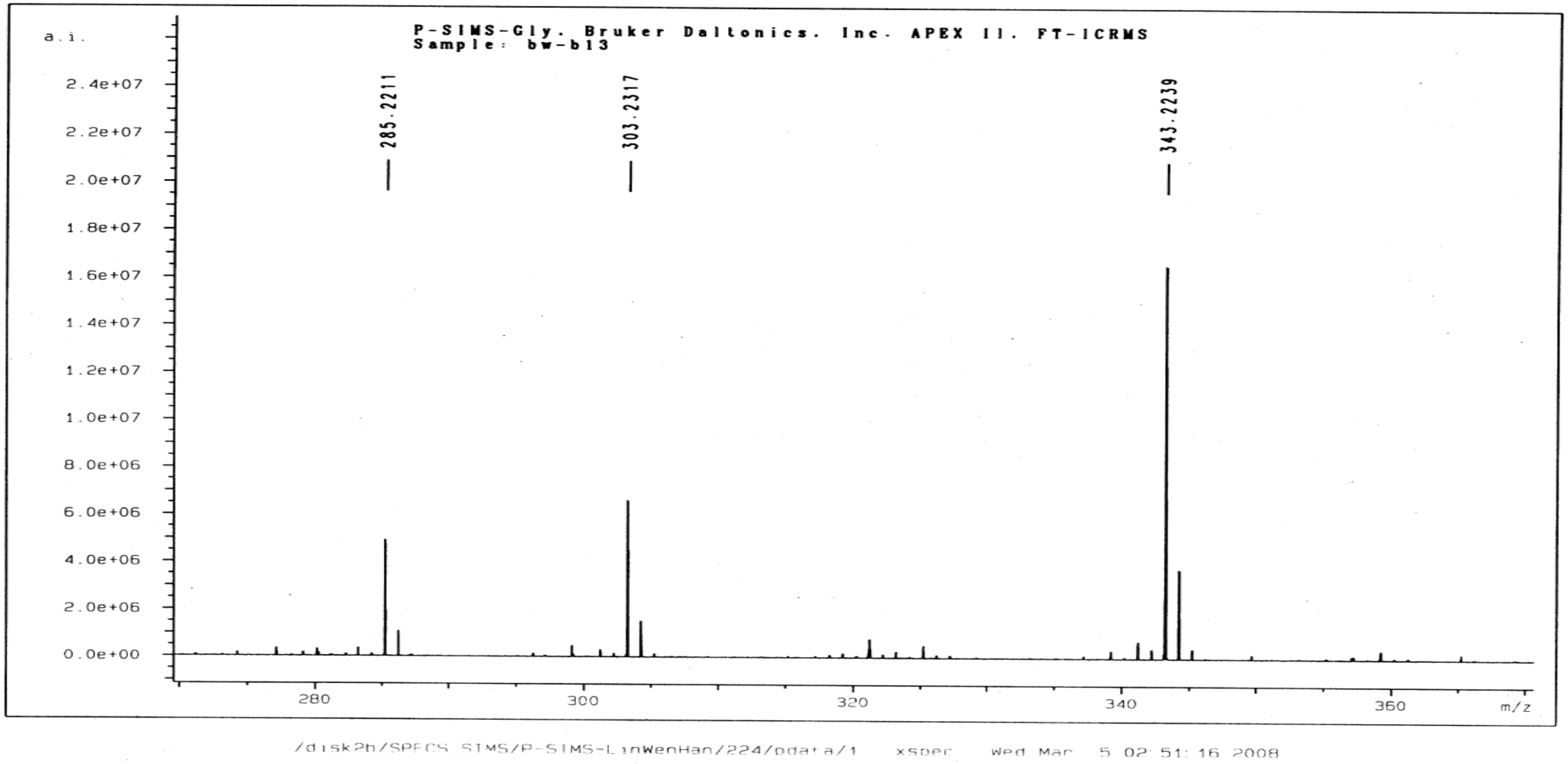

Figure S25. ^{1}H NMR data of sarcophyolide E (**4**).

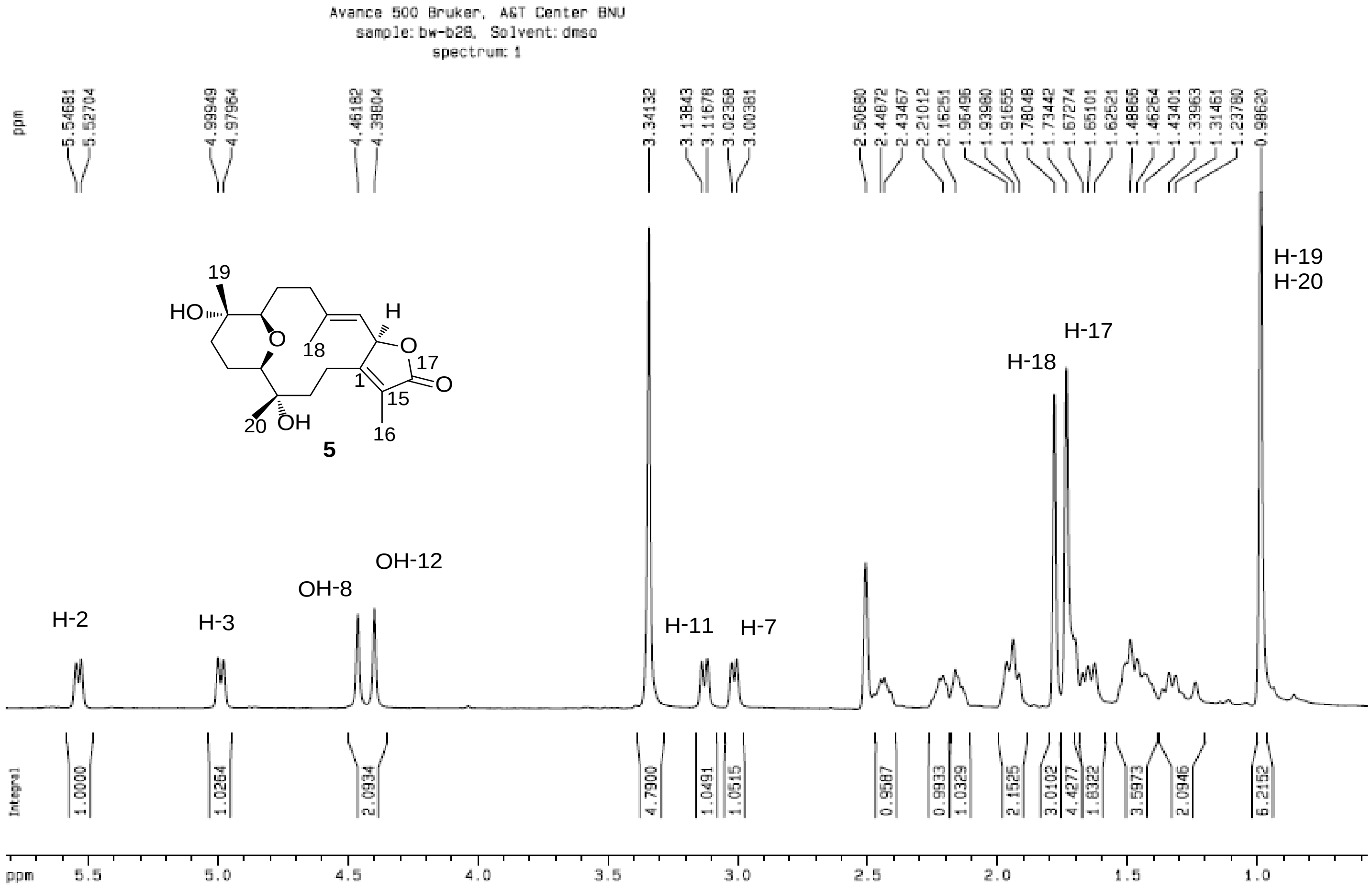

Figure S26. ^{13}C NMR data of sarcophyolide E (**4**).

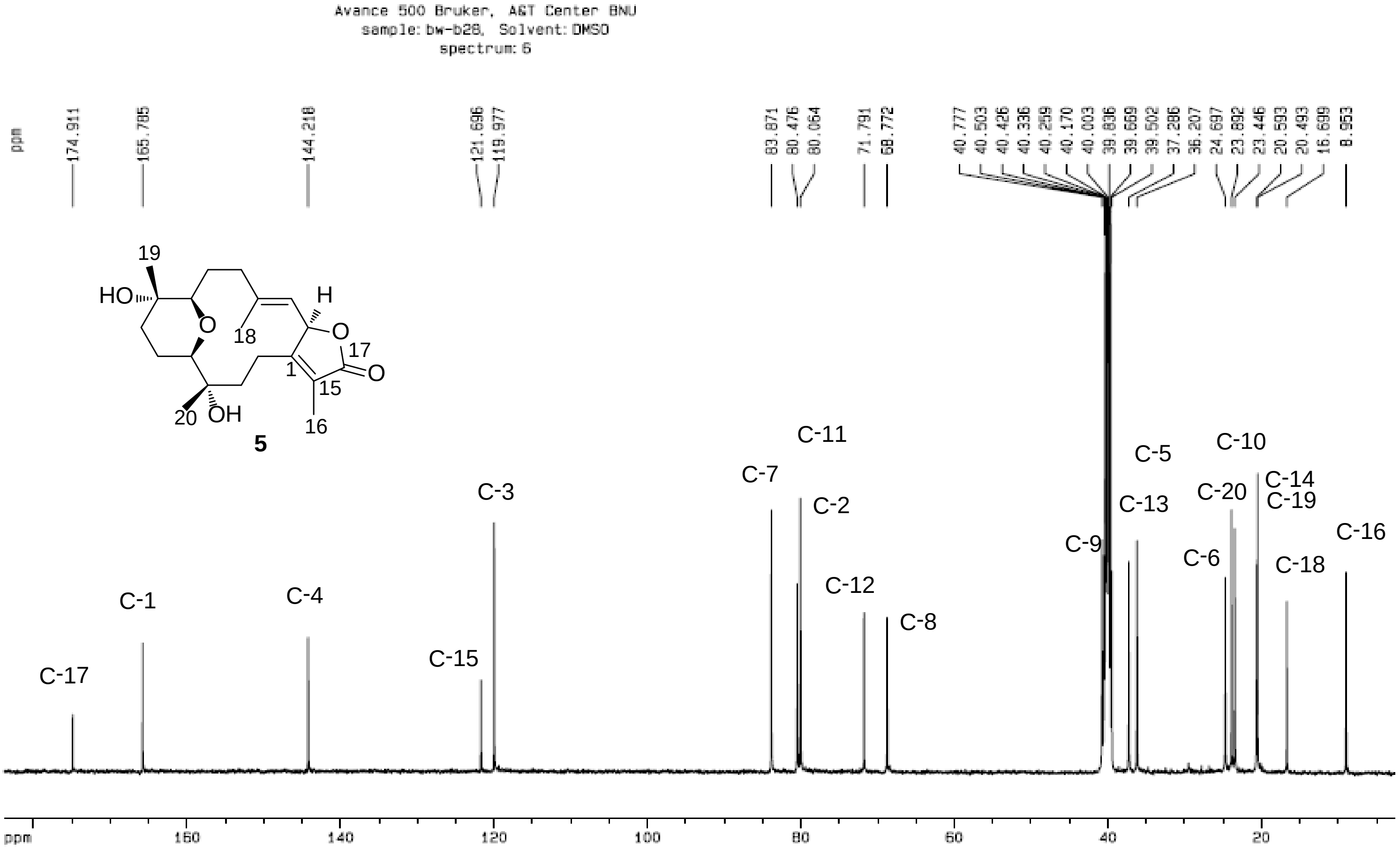

Figure S27. COSY spectrum of sarcophyolide E (**4**).

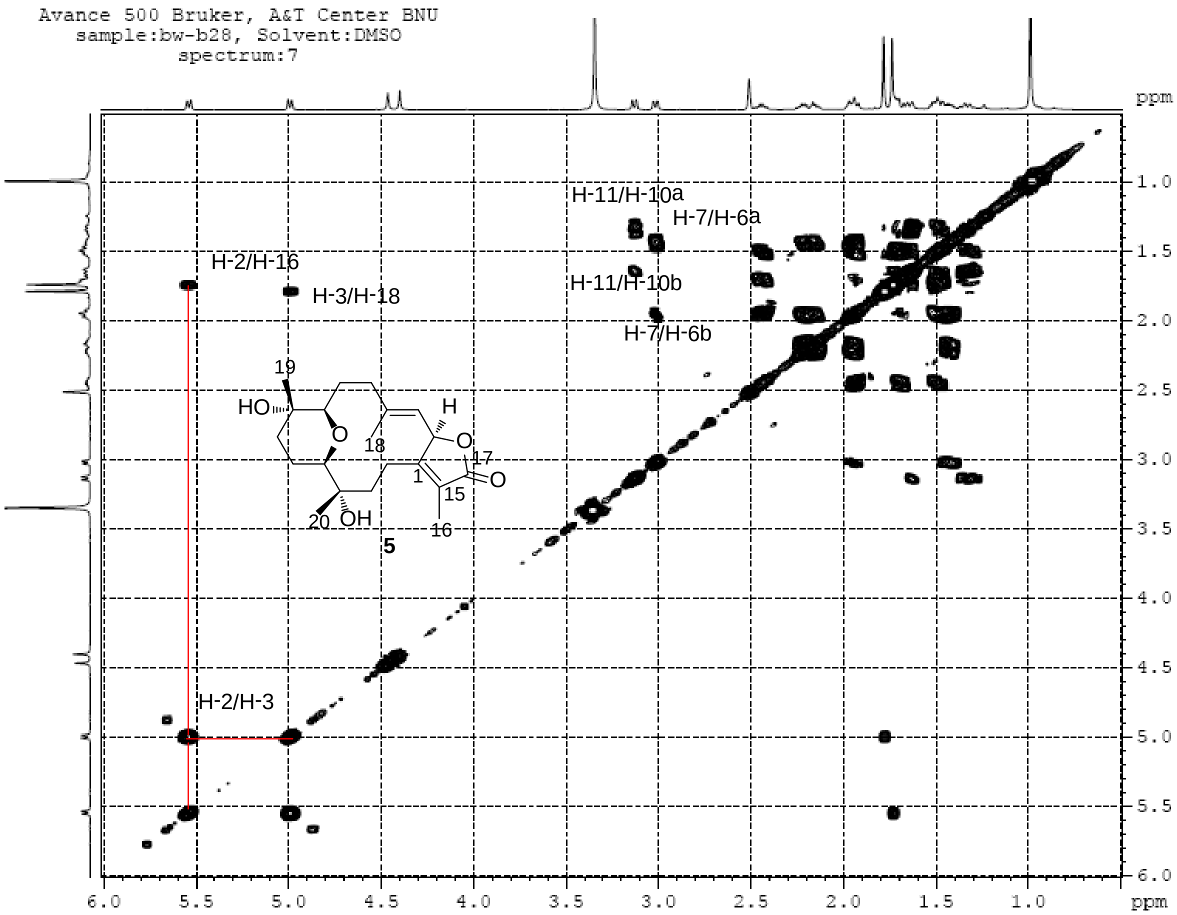

Figure S28. HMQC spectrum of sarcophyolide E (**4**).

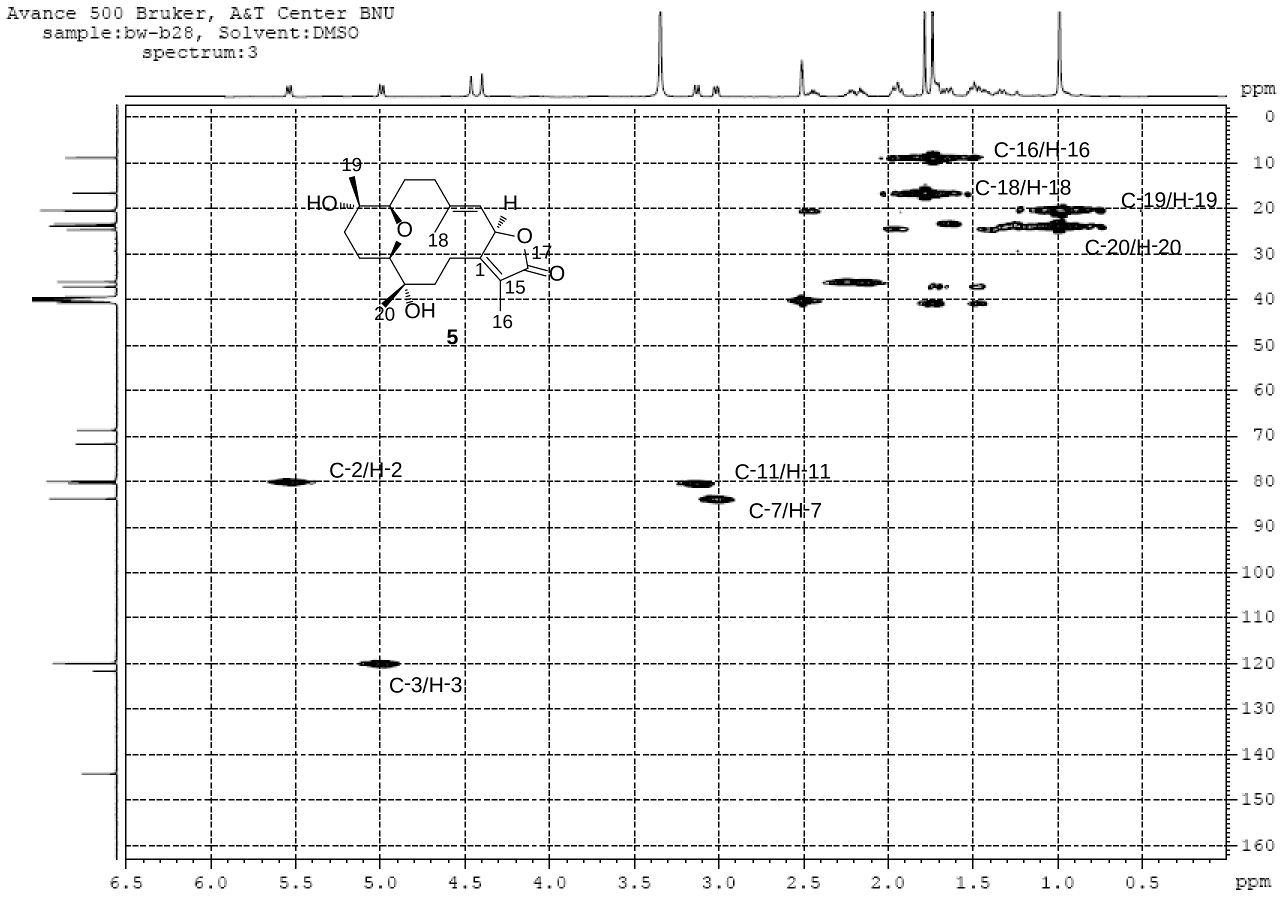

Figure S29. HMBC spectrum of sarcophyolide E (**4**).

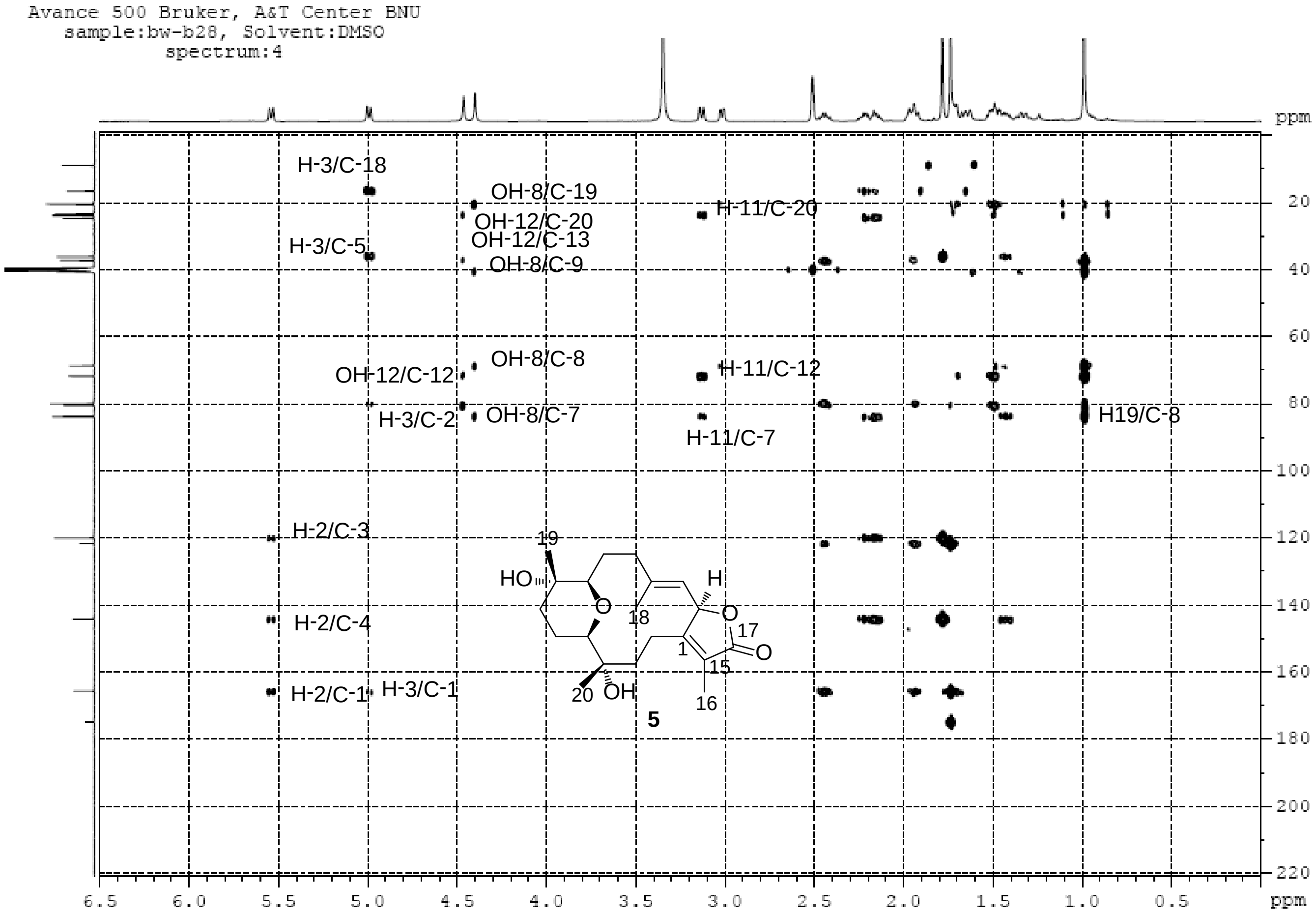

Figure S30. NOESY spectrum of sarcophyolide E (**4**).

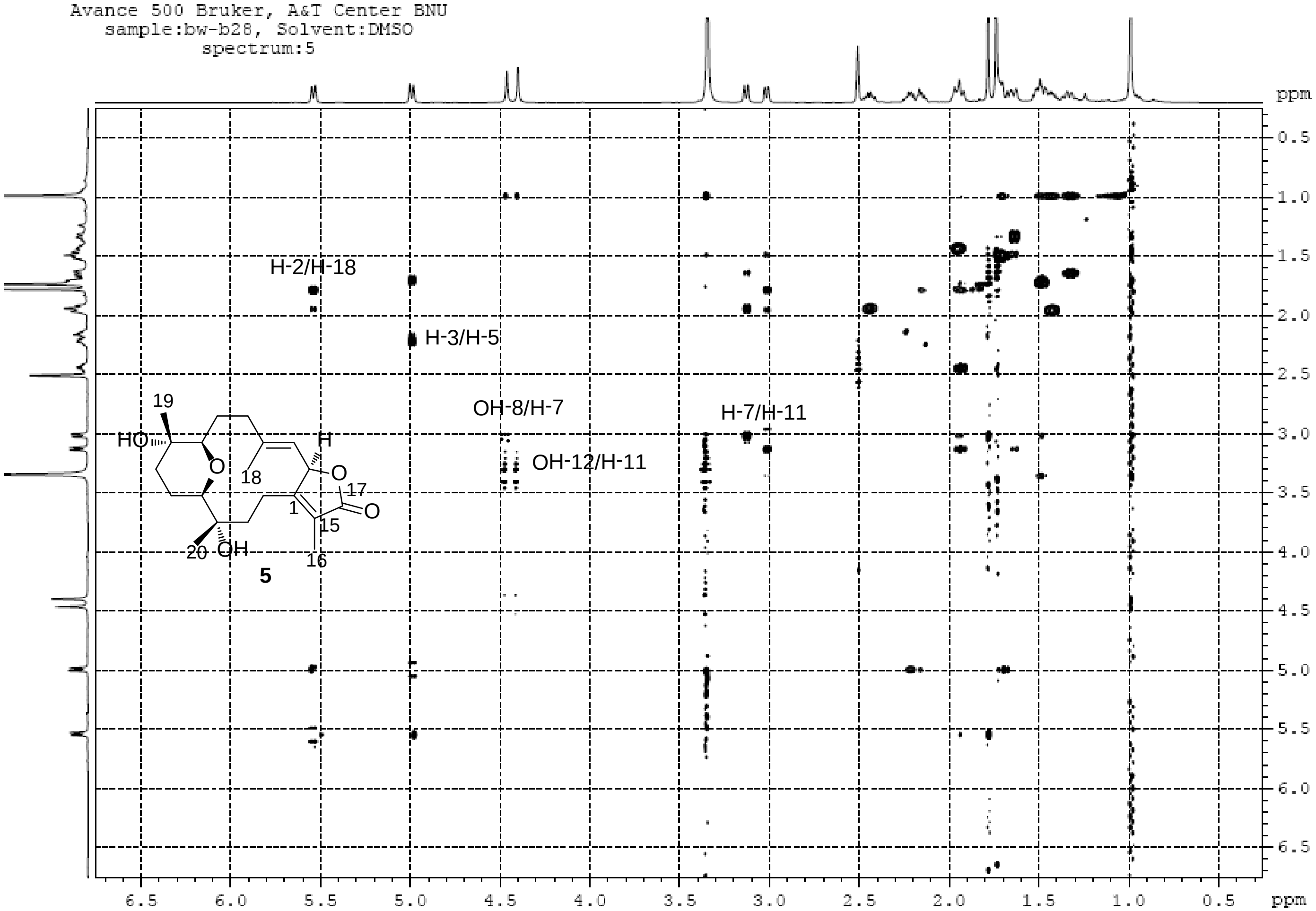

Figure S31. IR spectrum of sarcophyolide E (**4**).

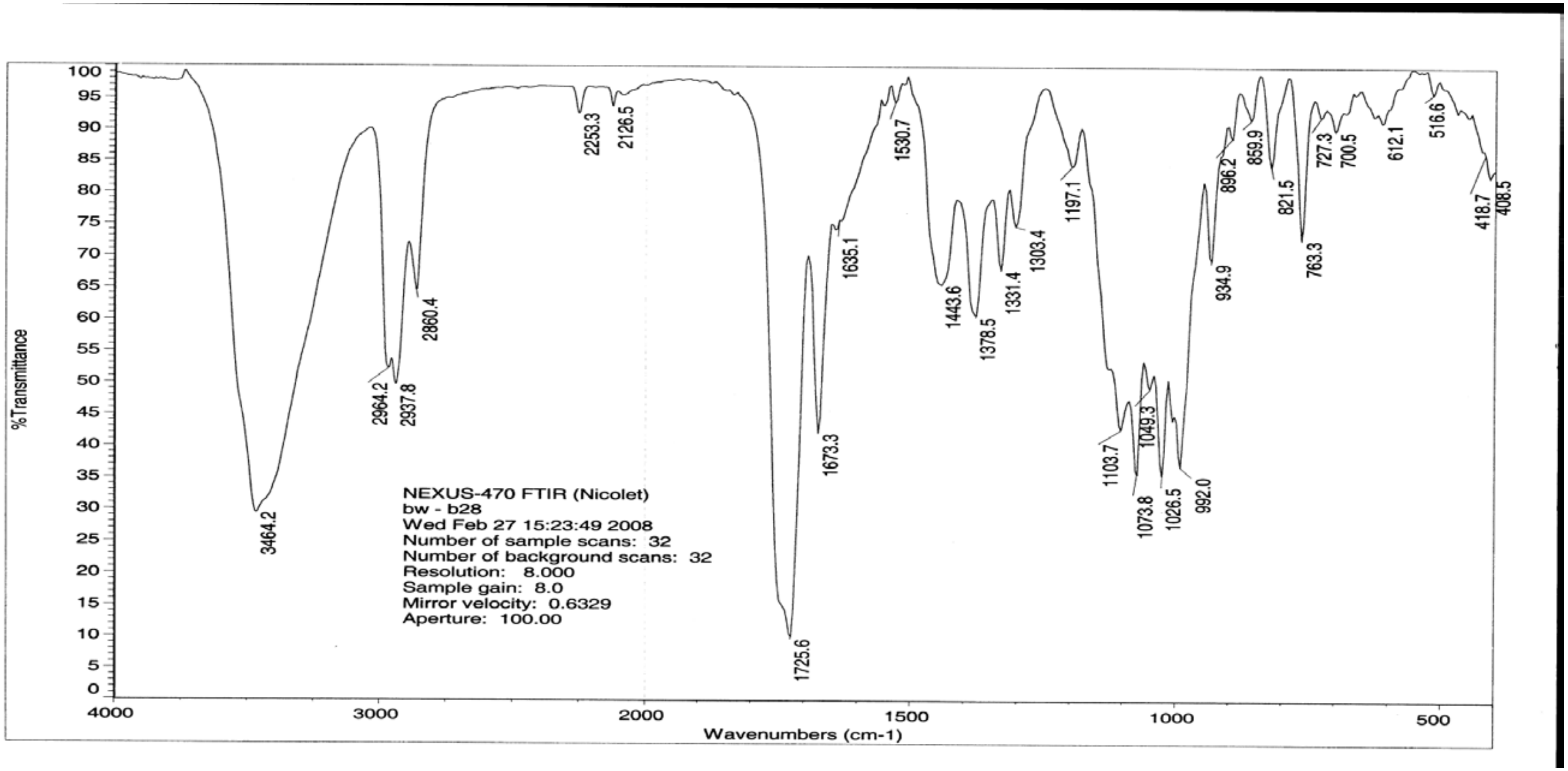

Figure S32. HRMS spectrum of sarcophyolide E (**4**).

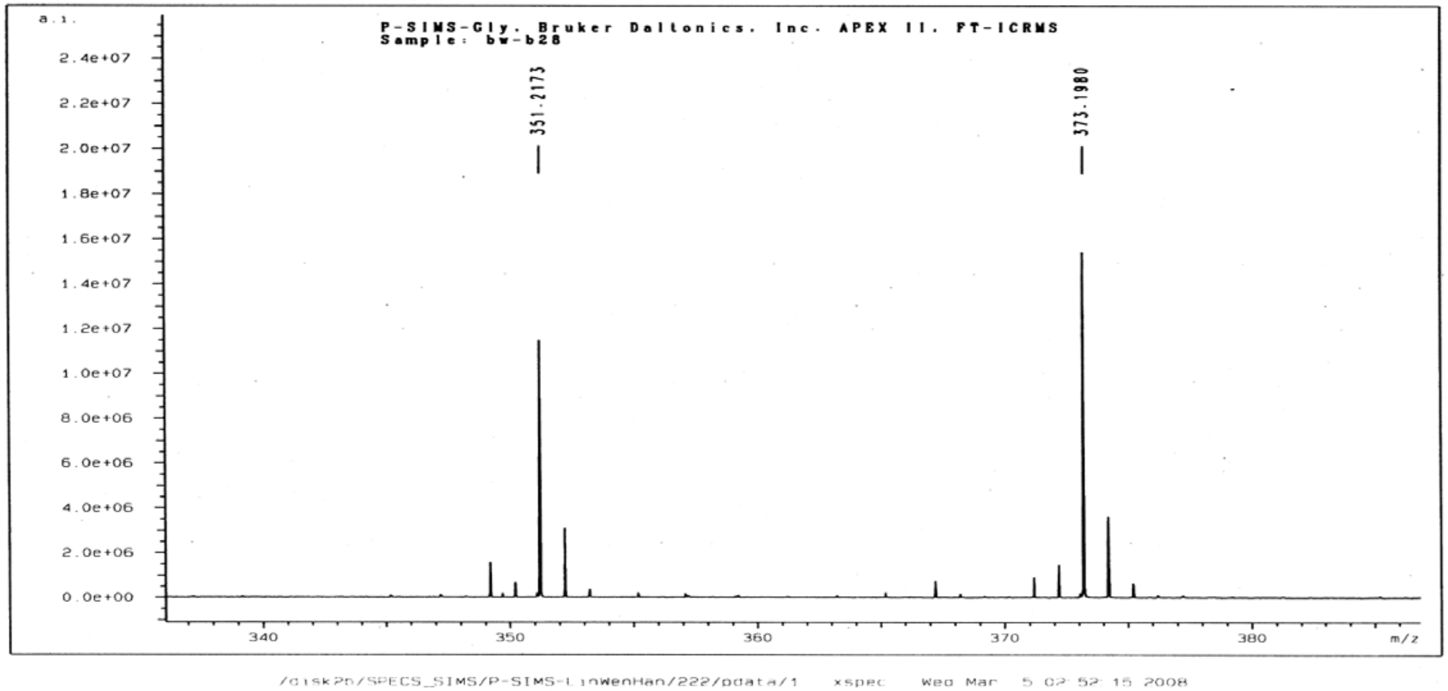

Figure S33. ORTEP depiction for X-ray crystal structure of **1**.

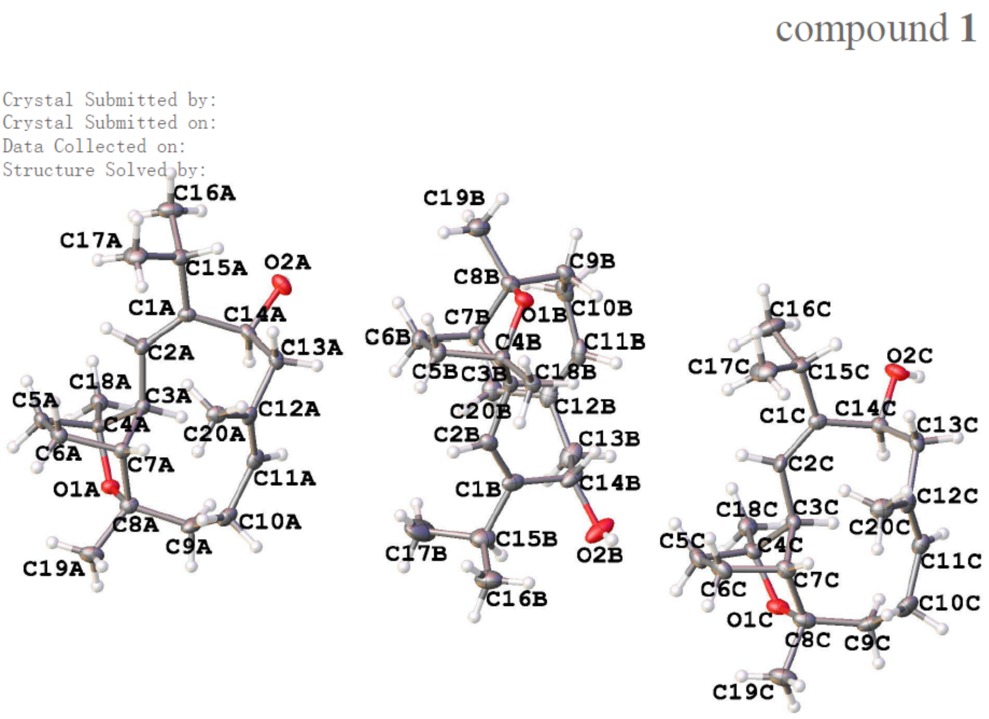

Table 1: Crystal data and structure refinement for compound **1**

Identification code	exp_1415
Empirical formula	$C_{20}H_{32}O_2$
Formula weight	304.46
Temperature/K	97.3
Crystal system	orthorhombic
Space group	$P2_12_12_1$
a/Å, b/Å, c/Å	9.9686(2), 11.4498(3), 47.9528(18)
α/°, β/°, γ/°	90.00, 90.00, 90.00
Volume/Å^3	5473.2(3)
Z	12
ρ_{calc}/mg mm^{-3}	1.108
μ/mm^{-1}	0.532
F(000)	2016
Crystal size/mm^3	0.40 × 0.30 × 0.15
2Θ range for data collection	7.38 to 141°
Index ranges	$-10 \leqslant h \leqslant 12$, $-13 \leqslant k \leqslant 10$, $-58 \leqslant l \leqslant 57$
Reflections collected	20784
Independent reflections	10157[R(int) = 0.0455 (inf-0.9Å)]
Data/restraints/parameters	10157/0/613
Goodness-of-fit on F^2	1.087
Final R indexes [I>2σ (I) i.e. F_o>4σ (F_o)]	R_1 = 0.0526, wR_2 = 0.1243
Final R indexes [all data]	R_1 = 0.0592, wR_2 = 0.1279
Largest diff. peak/hole/e Å^{-3}	0.216/-0.296
Flack Parameters	0.08(19)
Completeness	0.978

Table 2 Fractional Atomic Coordinates (×10^4) and Equivalent Isotropic Displacement Parameters (Å^2×10^3) for compound **1**. U_{eq} is defined as 1/3 of of the trace of the orthogonalised U_{IJ} tensor.

Atom	*x*	*y*	*z*	U(eq)
O1A	-15614.6(15)	-7067.6(13)	-391.5(3)	16.5(3)
O2A	-12377.5(18)	-2848.5(14)	-952.1(3)	22.3(4)
C1A	-12595(2)	-3787.4(18)	-500.1(5)	15.1(4)
C2A	-13343(2)	-4464.3(19)	-334.0(4)	15.4(5)
C3A	-14043(2)	-5576(2)	-414.4(4)	14.6(4)
C4A	-15397(2)	-5894.8(19)	-278.4(4)	15.1(5)
C5A	-15057(2)	-6048(2)	32.1(5)	17.5(5)
C6A	-13613(2)	-6559(2)	20.4(5)	18.9(5)
C7A	-13369(2)	-6692.0(19)	-296.0(5)	16.0(5)
C8A	-14292(2)	-7634(2)	-419.9(5)	18.4(5)
C9A	-14011(2)	-7874(2)	-734.1(5)	21.2(5)
C10A	-12514(3)	-7836(2)	-823.2(5)	22.3(5)
C11A	-12107(2)	-6622(2)	-907.8(5)	19.9(5)
C12A	-11213(2)	-5891(2)	-799.5(5)	17.4(5)
C13A	-11181(2)	-4632(2)	-898.3(5)	18.8(5)
C14A	-12457(2)	-3955(2)	-814.9(5)	17.1(5)
C15A	-11787(2)	-2759(2)	-384.0(5)	19.2(5)
C16A	-12654(3)	-1660(2)	-354.8(6)	28.9(6)
C17A	-11071(3)	-3032(2)	-109.0(5)	29.0(6)
C18A	-16622(2)	-5168(2)	-341.4(5)	18.4(5)
C19A	-14345(3)	-8786(2)	-263.1(6)	25.8(6)
C20A	-10234(2)	-6173(2)	-570.0(5)	19.9(5)
O1C	-15154.0(17)	-7162.9(14)	-3850.3(4)	22.0(4)
O2C	-12620.7(18)	-2299.2(14)	-4159.1(4)	23.0(4)
C1C	-12511(2)	-3647.0(19)	-3780.8(5)	16.5(5)
C2C	-13107(2)	-4546(2)	-3652.0(4)	17.3(5)
C3C	-13705(2)	-5604(2)	-3790.6(5)	16.5(5)
C4C	-15030(3)	-6112(2)	-3678.5(5)	19.9(5)
C5C	-14677(3)	-6536(2)	-3385.8(5)	25.6(6)
C6C	-13205(3)	-6949(2)	-3421.1(5)	26.7(6)
C7C	-12941(2)	-6760(2)	-3735.4(5)	20.6(5)
C8C	-13794(3)	-7589(2)	-3913.3(6)	23.9(5)
C9C	-13564(3)	-7469(2)	-4231.8(6)	25.8(6)
C10C	-12103(3)	-7242(2)	-4327.3(6)	28.5(6)
C11C	-11827(2)	-5945(2)	-4344.7(5)	21.9(5)
C12C	-10987(2)	-5282(2)	-4202.1(5)	20.7(5)
C13C	-11128(2)	-3961(2)	-4220.5(5)	20.7(5)
C14C	-12453(2)	-3509.5(19)	-4095.5(5)	16.8(5)
C15C	-11841(3)	-2659(2)	-3618.0(5)	21.5(5)
C16C	-12894(3)	-1816(2)	-3506.4(7)	37.8(7)
C17C	-10934(3)	-3092(2)	-3382.1(6)	36.1(7)
C18C	-16287(2)	-5405(2)	-3713.8(5)	23.3(5)
C19C	-13712(3)	-8879(2)	-3832.9(7)	34.4(7)
C20C	-9902(3)	-5711(2)	-4009.0(5)	26.0(6)
O1B	-9612.1(16)	-2767.7(14)	-2036.5(3)	18.5(3)
O2B	-12659.4(19)	-6705.9(19)	-2743.4(4)	37.2(5)
C1B	-12421(2)	-6070(2)	-2257.6(5)	22.6(5)
C2B	-11750(2)	-5455(2)	-2067.9(5)	19.1(5)
C3B	-11126(2)	-4270(2)	-2107.2(4)	15.7(5)
C4B	-9771(2)	-4009(2)	-1967.6(5)	16.5(5)
C5B	-10074(2)	-4069(2)	-1653.2(5)	20.3(5)
C6B	-11545(2)	-3613(2)	-1638.6(5)	20.0(5)
C7B	-11829(2)	-3273(2)	-1944.3(5)	17.6(5)
C8B	-10952(2)	-2236(2)	-2035.0(5)	19.8(5)
C9B	-11277(3)	-1776(2)	-2332.0(5)	23.0(5)
C10B	-12766(3)	-1851(2)	-2422.9(5)	25.4(6)
C11B	-13076(2)	-3008(2)	-2553.8(5)	24.5(6)

C12B	-13922(2)	-3846(2)	-2475.3(5)	22.7(5)
C13B	-13859(3)	-5017(3)	-2622.2(6)	29.9(6)
C14B	-12571(3)	-5717(2)	-2562.7(5)	25.9(6)
C15B	-13121(3)	-7213(2)	-2178.5(6)	33.1(6)
C16B	-12142(3)	-8235(2)	-2177.1(7)	37.5(7)
C17B	-13848(4)	-7148(3)	-1897.3(8)	62.3(12)
C18B	-8547(2)	-4658(2)	-2065.2(5)	18.2(5)
C19B	-10909(3)	-1200(2)	-1834.1(6)	28.7(6)
C20B	-14931(2)	-3763(2)	-2242.7(5)	23.0(5)

Table 3 Anisotropic Displacement Parameters ($Å^2 \times 10^3$) for compound **1**. The Anisotropic displacement factor exponent takes the form: $-2\pi^2[h^2a^{*2}U_{11}+...+2hka \times b \times U_{12}]$

Atom	U_{11}	U_{22}	U_{33}	U_{23}	U_{13}	U_{12}
O1A	13.3(8)	14.6(8)	21.5(8)	-1.7(7)	0.2(6)	-0.5(7)
O2A	18.5(9)	22.8(8)	25.6(9)	10.8(7)	-2.3(7)	-0.6(8)
C1A	11.8(11)	13.2(10)	20.2(11)	-0.7(8)	-2.2(9)	1.5(9)
C2A	14.4(11)	16.7(11)	15.1(10)	-2.4(9)	0.6(8)	1.5(9)
C3A	13.4(11)	16.1(11)	14.4(10)	-1.1(9)	0.1(8)	0.9(9)
C4A	18.2(12)	12.0(11)	15.1(10)	0.1(9)	0.7(9)	-1.2(9)
C5A	17.3(12)	17.9(11)	17.3(11)	2.9(9)	3.0(9)	-1.8(10)
C6A	18.7(12)	19.5(11)	18.6(11)	1.5(9)	-0.8(9)	-1.3(10)
C7A	13.3(11)	15.7(11)	18.9(11)	0.0(9)	-0.5(9)	0.9(9)
C8A	14.4(11)	19.0(12)	21.8(11)	-2.3(10)	-1.1(9)	-0.4(10)
C9A	17.8(13)	19.9(12)	25.8(12)	-8.8(10)	0.6(10)	-3.8(10)
C10A	18.6(13)	24.3(12)	24.1(12)	-9.4(10)	1.7(10)	2.7(11)
C11A	13.2(12)	27.2(12)	19.3(11)	-4.7(10)	3.0(9)	5.1(10)
C12A	13.9(11)	21.5(12)	16.6(10)	-3.6(9)	4.8(9)	3.4(10)
C13A	14.9(12)	24.3(12)	17.3(10)	3.0(9)	2.6(9)	-1.2(10)
C14A	13.6(11)	18.1(11)	19.5(11)	5.2(9)	-0.5(9)	-4(1)
C15A	16.3(12)	18.1(11)	23.3(11)	-3.3(10)	2.2(10)	-3(1)
C16A	24.4(14)	17.4(12)	45.0(16)	-4.1(11)	-0.1(12)	-1.7(11)
C17A	28.8(15)	25.0(14)	33.2(14)	-3.6(11)	-9.0(11)	-8.5(12)
C18A	16.2(12)	19.2(11)	20.0(11)	1.5(9)	0.0(9)	-0.5(10)
C19A	23.1(14)	16.4(12)	38.1(14)	0.1(11)	-1.4(11)	0.1(10)
C20A	14.1(12)	18.4(12)	27.4(12)	-2.1(10)	-0.2(10)	2.5(10)
O1C	17.5(9)	17.0(8)	31.4(9)	0.4(7)	-3.8(7)	-3.8(7)
O2C	23.9(10)	15.9(8)	29.3(9)	6.9(7)	-7.7(7)	-3.6(7)
C1C	11.4(11)	16.8(11)	21.4(11)	-2.3(9)	-1.4(9)	1.4(10)
C2C	17.3(12)	22.2(12)	12.4(10)	-2.7(9)	0.0(9)	-1(1)
C3C	16.8(12)	17.6(11)	15(1)	2.0(9)	-0.6(9)	-2.5(10)
C4C	20.6(13)	17.5(11)	21.6(11)	2.7(10)	-0.1(10)	-5.1(10)
C5C	25.5(14)	27.9(13)	23.4(12)	7.2(11)	0.6(10)	-9.5(11)
C6C	28.8(15)	24.4(13)	26.9(13)	8.3(10)	-6.4(11)	-4.1(11)
C7C	17.2(12)	20.1(11)	24.6(12)	2.9(10)	-2.8(10)	-0.4(10)
C8C	18.4(13)	17.8(12)	35.6(14)	0.1(10)	-1.3(11)	-1.1(10)
C9C	26.9(14)	18.1(12)	32.5(13)	-9.8(10)	-5.2(11)	1.9(11)
C10C	27.8(15)	28.9(14)	28.8(13)	-11.7(11)	1.0(11)	7.4(12)
C11C	18.7(13)	26.8(13)	20.1(11)	-3.4(10)	1.1(10)	3.7(11)
C12C	16.1(12)	26.4(13)	19.7(11)	-1.9(10)	5.8(9)	3.6(11)
C13C	17.1(12)	27.7(13)	17.2(11)	3.4(10)	2.4(9)	-4.0(11)
C14C	15.0(11)	16.2(11)	19.3(11)	1.2(9)	-2.7(9)	-1(1)
C15C	20.8(13)	19.7(12)	24.0(12)	-3.3(10)	-2.2(10)	-5.4(10)
C16C	38.0(18)	26.7(14)	48.7(17)	-17.9(13)	-3.7(14)	1.1(13)
C17C	40.9(18)	30.8(15)	36.7(15)	-4.5(12)	-16.4(13)	-6.5(13)
C18C	19.0(13)	24.8(13)	26.3(12)	-0.5(10)	3.1(10)	-3.6(11)
C19C	32.3(16)	18.9(13)	52.0(18)	2.9(12)	-5.9(14)	-1.8(12)
C20C	17.4(13)	25.6(13)	35.1(14)	-2.8(11)	-1.4(10)	2.1(11)
O1B	15.8(8)	15.9(8)	23.7(8)	1.5(7)	0.4(7)	-1.2(7)
O2B	16.3(9)	49.1(12)	46.3(12)	-30.5(10)	0.8(9)	-2.7(9)
C1B	12.0(12)	20.8(12)	34.8(13)	-8(1)	5(1)	-0.8(10)

C2B	15.5(12)	17.0(11)	24.9(12)	1.7(9)	2.2(10)	2(1)
C3B	14.9(11)	17.5(11)	14.8(10)	0.5(9)	0.3(9)	2.2(10)
C4B	17.7(12)	15.3(11)	16.5(11)	-0.3(9)	-0.1(9)	-0.2(9)
C5B	19.4(13)	25.1(12)	16.4(11)	0.9(10)	-1.5(9)	-0.8(10)
C6B	21.3(13)	22.9(12)	15.8(11)	-2.2(9)	2.6(9)	-2.1(10)
C7B	15.3(12)	19.8(11)	17.7(11)	-0.4(9)	0.1(9)	-0.9(10)
C8B	16.4(12)	18.8(12)	24.1(12)	1.6(10)	-0.2(9)	-1.6(10)
C9B	21.4(13)	20.8(12)	26.7(12)	7.2(10)	2.7(10)	1.8(11)
C10B	22.1(13)	29.3(14)	24.8(12)	11.1(10)	0.2(10)	4.0(11)
C11B	17.2(13)	41.5(16)	14.7(11)	3.8(10)	-0.5(9)	7.1(12)
C12B	12.6(12)	36.5(15)	19.1(11)	-0.9(10)	-4.3(9)	5.3(11)
C13B	16.6(14)	45.1(17)	28.1(13)	-10.2(12)	-4.6(10)	-0.6(12)
C14B	15.5(12)	33.1(14)	29.3(13)	-15.2(11)	1(1)	-3.7(12)
C15B	26.3(15)	28.2(14)	44.9(16)	-6.1(13)	2.1(12)	-6.1(13)
C16B	40.5(18)	23.8(14)	48.2(18)	-3.6(12)	-5.6(14)	-5.7(13)
C17B	61(3)	36.9(18)	89(3)	-8.5(19)	44(2)	-22.5(18)
C18B	15.6(12)	20.9(12)	18.3(11)	2.0(9)	-0.5(9)	2.1(10)
C19B	29.0(15)	21.7(13)	35.3(14)	-2.9(11)	2.4(12)	-1.5(12)
C20B	13.6(12)	29.0(14)	26.4(12)	0.6(11)	-0.7(10)	2.1(11)

Table 4 Bond Lengths for compound **1**.

Atom	Atom	Length/Å	Atom	Atom	Length/Å
O1A	C4A	1.464(3)	C7C	C8C	1.534(3)
O1A	C8A	1.476(3)	C8C	C9C	1.551(4)
O2A	C14A	1.429(3)	C8C	C19C	1.528(3)
C1A	C2A	1.338(3)	C10C	C9C	1.548(4)
C1A	C14A	1.527(3)	C10C	C11C	1.512(4)
C1A	C15A	1.531(3)	C12C	C11C	1.322(4)
C2A	C3A	1.503(3)	C12C	C13C	1.522(3)
C3A	C7A	1.551(3)	C12C	C20C	1.506(3)
C4A	C3A	1.542(3)	C14C	C13C	1.540(3)
C4A	C18A	1.508(3)	C15C	C16C	1.523(4)
C5A	C4A	1.537(3)	C15C	C17C	1.531(3)
C5A	C6A	1.555(3)	C18C	C4C	1.501(3)
C6A	C7A	1.545(3)	O1B	C4B	1.467(3)
C8A	C7A	1.537(3)	O1B	C8B	1.468(3)
C8A	C9A	1.557(3)	O2B	C14B	1.428(3)
C8A	C19A	1.519(3)	C1B	C14B	1.525(4)
C10A	C9A	1.552(3)	C1B	C15B	1.530(4)
C11A	C10A	1.504(3)	C2B	C1B	1.331(3)
C11A	C12A	1.328(3)	C2B	C3B	1.504(3)
C13A	C12A	1.518(3)	C4B	C3B	1.537(3)
C14A	C13A	1.543(3)	C4B	C5B	1.539(3)
C15A	C16A	1.533(3)	C4B	C18B	1.503(3)
C15A	C17A	1.532(3)	C6B	C5B	1.558(3)
C20A	C12A	1.506(3)	C7B	C3B	1.550(3)
O1C	C4C	1.464(3)	C7B	C6B	1.543(3)
O1C	C8C	1.472(3)	C7B	C8B	1.537(3)
O2C	C14C	1.429(3)	C8B	C9B	1.553(3)
C1C	C2C	1.340(3)	C8B	C19B	1.529(3)
C1C	C14C	1.518(3)	C9B	C10B	1.549(4)
C1C	C15C	1.528(3)	C11B	C10B	1.498(4)
C3C	C2C	1.505(3)	C11B	C12B	1.332(4)
C3C	C4C	1.540(3)	C13B	C12B	1.515(4)
C3C	C7C	1.550(3)	C13B	C14B	1.540(4)
C4C	C5C	1.526(3)	C15B	C16B	1.524(4)
C6C	C5C	1.551(4)	C15B	C17B	1.533(4)
C7C	C6C	1.545(3)	C20B	C12B	1.505(3)

Table 5 Bond Angles for compound **1**.

Atom	Atom	Atom	Angle/˚	Atom	Atom	Atom	Angle/˚
C4A	O1A	C8A	107.72(16)	O1C	C8C	C7C	101.03(19)
C2A	C1A	C14A	124.4(2)	O1C	C8C	C9C	108.0(2)
C2A	C1A	C15A	121.5(2)	O1C	C8C	C19C	108.5(2)
C14A	C1A	C15A	114.10(19)	C7C	C8C	C9C	114.2(2)
C1A	C2A	C3A	126.6(2)	C19C	C8C	C7C	115.3(2)
C2A	C3A	C4A	119.89(18)	C19C	C8C	C9C	109.1(2)
C2A	C3A	C7A	113.73(18)	C10C	C9C	C8C	116.5(2)
C4A	C3A	C7A	91.71(17)	C11C	C10C	C9C	110.6(2)
O1A	C4A	C3A	100.95(17)	C12C	C11C	C10C	130.7(2)
O1A	C4A	C5A	106.65(17)	C11C	C12C	C13C	118.9(2)
O1A	C4A	C18A	108.15(18)	C11C	C12C	C20C	125.8(2)
C5A	C4A	C3A	104.11(18)	C20C	C12C	C13C	115.2(2)
C18A	C4A	C3A	119.58(19)	C12C	C13C	C14C	113.0(2)
C18A	C4A	C5A	115.79(19)	O2C	C14C	C1C	107.94(18)
C4A	C5A	C6A	102.24(18)	O2C	C14C	C13C	110.04(19)
C7A	C6A	C5A	102.63(18)	C1C	C14C	C13C	112.60(19)
C6A	C7A	C3A	102.12(18)	C1C	C15C	C17C	113.3(2)
C8A	C7A	C3A	100.18(18)	C16C	C15C	C1C	110.4(2)
C8A	C7A	C6A	110.75(19)	C16C	C15C	C17C	110.7(2)
O1A	C8A	C7A	101.03(17)	C4B	O1B	C8B	107.59(17)
O1A	C8A	C9A	109.12(18)	C2B	C1B	C14B	124.4(2)
O1A	C8A	C19A	107.70(19)	C2B	C1B	C15B	120.8(2)
C7A	C8A	C9A	112.99(19)	C14B	C1B	C15B	114.8(2)
C19A	C8A	C7A	116.0(2)	C1B	C2B	C3B	126.8(2)
C19A	C8A	C9A	109.4(2)	C2B	C3B	C4B	118.98(19)
C10A	C9A	C8A	115.71(19)	C2B	C3B	C7B	114.46(19)
C11A	C10A	C9A	111.1(2)	C4B	C3B	C7B	91.96(17)
C12A	C11A	C10A	131.2(2)	O1B	C4B	C3B	100.67(17)
C11A	C12A	C13A	119.4(2)	O1B	C4B	C5B	106.56(18)
C11A	C12A	C20A	125.8(2)	O1B	C4B	C18B	108.73(18)
C20A	C12A	C13A	114.7(2)	C3B	C4B	C5B	104.19(19)
C12A	C13A	C14A	112.27(19)	C18B	C4B	C3B	118.78(19)
O2A	C14A	C1A	110.41(18)	C18B	C4B	C5B	116.2(2)
O2A	C14A	C13A	106.26(18)	C4B	C5B	C6B	102.35(18)
C1A	C14A	C13A	113.17(19)	C7B	C6B	C5B	102.37(18)
C1A	C15A	C16A	111.6(2)	C6B	C7B	C3B	102.15(18)
C1A	C15A	C17A	113.6(2)	C8B	C7B	C3B	99.74(18)
C17A	C15A	C16A	110.6(2)	C8B	C7B	C6B	111.07(19)
C4C	O1C	C8C	108.10(18)	O1B	C8B	C7B	101.47(18)
C2C	C1C	C14C	123.7(2)	O1B	C8B	C9B	109.04(19)
C2C	C1C	C15C	121.8(2)	O1B	C8B	C19B	107.4(2)
C14C	C1C	C15C	114.51(19)	C7B	C8B	C9B	113.8(2)
C1C	C2C	C3C	126.2(2)	C19B	C8B	C7B	115.9(2)
C2C	C3C	C4C	119.27(19)	C19B	C8B	C9B	108.7(2)
C2C	C3C	C7C	114.69(19)	C10B	C9B	C8B	116.1(2)
C4C	C3C	C7C	92.28(17)	C11B	C10B	C9B	111.4(2)
O1C	C4C	C3C	100.72(18)	C12B	C11B	C10B	130.5(2)
O1C	C4C	C5C	105.97(19)	C11B	C12B	C13B	118.7(2)
O1C	C4C	C18C	108.0(2)	C11B	C12B	C20B	125.9(2)
C5C	C4C	C3C	104.1(2)	C20B	C12B	C13B	115.3(2)
C18C	C4C	C3C	118.3(2)	C12B	C13B	C14B	114.2(2)
C18C	C4C	C5C	117.9(2)	O2B	C14B	C1B	112.2(2)
C4C	C5C	C6C	102.4(2)	O2B	C14B	C13B	104.4(2)
C7C	C6C	C5C	103.0(2)	C1B	C14B	C13B	113.4(2)
C6C	C7C	C3C	101.70(19)	C1B	C15B	C17B	113.1(2)
C8C	C7C	C3C	99.26(18)	C16B	C15B	C1B	111.5(2)
C8C	C7C	C6C	111.2(2)	C16B	C15B	C17B	109.6(3)

Table 6 Torsion Angles for compound **1**.

A	B	C	D	Angle/°
O1A	C4A	C3A	C2A	-173.91(18)
O1A	C4A	C3A	C7A	-55.13(18)
O1A	C8A	C7A	C3A	-39.2(2)
O1A	C8A	C7A	C6A	68.1(2)
O1A	C8A	C9A	C10A	149.0(2)
O2A	C14A	C13A	C12A	-172.26(18)
C1A	C2A	C3A	C4A	-146.2(2)
C1A	C2A	C3A	C7A	106.9(3)
C1A	C14A	C13A	C12A	66.4(3)
C2A	C1A	C14A	O2A	143.5(2)
C2A	C1A	C14A	C13A	-97.6(3)
C2A	C1A	C15A	C16A	-83.9(3)
C2A	C1A	C15A	C17A	42.0(3)
C2A	C3A	C7A	C6A	67.2(2)
C2A	C3A	C7A	C8A	-178.80(18)
C4A	O1A	C8A	C7A	3.3(2)
C4A	O1A	C8A	C9A	-115.97(19)
C4A	O1A	C8A	C19A	125.40(19)
C4A	C3A	C7A	C6A	-56.70(19)
C4A	C3A	C7A	C8A	57.30(18)
C4A	C5A	C6A	C7A	-3.2(2)
C5A	C4A	C3A	C2A	-63.4(2)
C5A	C4A	C3A	C7A	55.34(19)
C5A	C6A	C7A	C3A	38.7(2)
C5A	C6A	C7A	C8A	-67.2(2)
C6A	C5A	C4A	O1A	72.5(2)
C6A	C5A	C4A	C3A	-33.8(2)
C6A	C5A	C4A	C18A	-167.16(19)
C7A	C8A	C9A	C10A	37.5(3)
C8A	O1A	C4A	C3A	34.1(2)
C8A	O1A	C4A	C5A	-74.4(2)
C8A	O1A	C4A	C18A	160.39(18)
C9A	C8A	C7A	C3A	77.3(2)
C9A	C8A	C7A	C6A	-175.50(19)
C10A	C11A	C12A	C13A	-169.4(2)
C10A	C11A	C12A	C20A	7.3(4)
C11A	C10A	C9A	C8A	-89.3(2)
C12A	C11A	C10A	C9A	116.9(3)
C14A	C1A	C2A	C3A	7.7(4)
C14A	C1A	C15A	C16A	96.3(2)
C14A	C1A	C15A	C17A	-137.8(2)
C14A	C13A	C12A	C11A	66.6(3)
C14A	C13A	C12A	C20A	-110.5(2)
C15A	C1A	C2A	C3A	-172.1(2)
C15A	C1A	C14A	O2A	-36.7(3)
C15A	C1A	C14A	C13A	82.2(2)
C18A	C4A	C3A	C2A	67.8(3)
C18A	C4A	C3A	C7A	-173.5(2)
C19A	C8A	C7A	C3A	-155.3(2)
C19A	C8A	C7A	C6A	-48.0(3)
C19A	C8A	C9A	C10A	-93.4(2)
O1C	C4C	C5C	C6C	71.9(2)
O1C	C8C	C9C	C10C	149.5(2)
O2C	C14C	C13C	C12C	-170.72(19)
C1C	C14C	C13C	C12C	68.8(3)
C2C	C1C	C14C	O2C	141.6(2)
C2C	C1C	C14C	C13C	-96.7(3)
C2C	C1C	C15C	C16C	-78.1(3)

C2C	C1C	C15C	C17C	46.6(3)
C2C	C3C	C4C	O1C	-174.46(19)
C2C	C3C	C4C	C5C	-64.8(3)
C2C	C3C	C4C	C18C	68.2(3)
C2C	C3C	C7C	C6C	67.9(2)
C2C	C3C	C7C	C8C	-178.08(19)
C3C	C4C	C5C	C6C	-33.8(2)
C3C	C7C	C6C	C5C	38.0(2)
C3C	C7C	C8C	O1C	-41.0(2)
C3C	C7C	C8C	C9C	74.7(2)
C3C	C7C	C8C	C19C	-157.8(2)
C4C	O1C	C8C	C7C	5.9(2)
C4C	O1C	C8C	C9C	-114.3(2)
C4C	O1C	C8C	C19C	127.6(2)
C4C	C3C	C2C	C1C	-140.5(2)
C4C	C3C	C7C	C6C	-56.0(2)
C4C	C3C	C7C	C8C	58.1(2)
C6C	C7C	C8C	O1C	65.5(2)
C6C	C7C	C8C	C9C	-178.8(2)
C6C	C7C	C8C	C19C	-51.3(3)
C7C	C3C	C2C	C1C	111.6(3)
C7C	C3C	C4C	O1C	-54.36(18)
C7C	C3C	C4C	C5C	55.3(2)
C7C	C3C	C4C	C18C	-171.7(2)
C7C	C6C	C5C	C4C	-2.8(2)
C7C	C8C	C9C	C10C	37.9(3)
C8C	O1C	C4C	C3C	31.7(2)
C8C	O1C	C4C	C5C	-76.4(2)
C8C	O1C	C4C	C18C	156.38(19)
C8C	C7C	C6C	C5C	-66.9(2)
C9C	C10C	C11C	C12C	115.9(3)
C11C	C10C	C9C	C8C	-90.8(3)
C11C	C12C	C13C	C14C	65.2(3)
C13C	C12C	C11C	C10C	-167.1(2)
C14C	C1C	C2C	C3C	5.6(4)
C14C	C1C	C15C	C16C	101.1(3)
C14C	C1C	C15C	C17C	-134.1(2)
C15C	C1C	C2C	C3C	-175.2(2)
C15C	C1C	C14C	O2C	-37.6(3)
C15C	C1C	C14C	C13C	84.1(2)
C18C	C4C	C5C	C6C	-167.1(2)
C19C	C8C	C9C	C10C	-92.7(3)
C20C	C12C	C11C	C10C	10.4(4)
C20C	C12C	C13C	C14C	-112.6(2)
O1B	C4B	C3B	C2B	-174.95(18)
O1B	C4B	C3B	C7B	-55.44(18)
O1B	C4B	C5B	C6B	73.0(2)
O1B	C8B	C9B	C10B	146.0(2)
C1B	C2B	C3B	C4B	-142.0(2)
C1B	C2B	C3B	C7B	110.8(3)
C2B	C1B	C14B	O2B	148.5(2)
C2B	C1B	C14B	C13B	-93.4(3)
C2B	C1B	C15B	C16B	-82.4(3)
C2B	C1B	C15B	C17B	41.7(4)
C3B	C2B	C1B	C14B	5.7(4)
C3B	C2B	C1B	C15B	-173.8(2)
C3B	C4B	C5B	C6B	-32.9(2)
C3B	C7B	C6B	C5B	39.2(2)
C3B	C7B	C8B	O1B	-38.8(2)
C3B	C7B	C8B	C9B	78.1(2)
C3B	C7B	C8B	C19B	-154.8(2)
C4B	O1B	C8B	C7B	3.0(2)

C4B	O1B	C8B	C9B	-117.4(2)
C4B	O1B	C8B	C19B	125.0(2)
C5B	C4B	C3B	C2B	-64.7(2)
C5B	C4B	C3B	C7B	54.9(2)
C6B	C7B	C3B	C2B	66.3(2)
C6B	C7B	C3B	C4B	-56.9(2)
C6B	C7B	C8B	O1B	68.3(2)
C6B	C7B	C8B	C9B	-174.7(2)
C6B	C7B	C8B	C19B	-47.7(3)
C7B	C6B	C5B	C4B	-4.0(2)
C7B	C8B	C9B	C10B	33.6(3)
C8B	O1B	C4B	C3B	34.4(2)
C8B	O1B	C4B	C5B	-74.0(2)
C8B	O1B	C4B	C18B	159.94(18)
C8B	C7B	C3B	C2B	-179.50(19)
C8B	C7B	C3B	C4B	57.26(19)
C8B	C7B	C6B	C5B	-66.3(2)
C8B	C9B	C10B	C11B	-87.7(3)
C10B	C11B	C12B	C13B	-169.8(2)
C10B	C11B	C12B	C20B	7.9(4)
C12B	C11B	C10B	C9B	117.8(3)
C12B	C13B	C14B	O2B	-173.8(2)
C12B	C13B	C14B	C1B	63.8(3)
C14B	C1B	C15B	C16B	98.0(3)
C14B	C1B	C15B	C17B	-138.0(3)
C14B	C13B	C12B	C11B	68.3(3)
C14B	C13B	C12B	C20B	-109.6(3)
C15B	C1B	C14B	O2B	-31.9(3)
C15B	C1B	C14B	C13B	86.2(3)
C18B	C4B	C3B	C2B	66.6(3)
C18B	C4B	C3B	C7B	-173.9(2)
C18B	C4B	C5B	C6B	-165.7(2)
C19B	C8B	C9B	C10B	-97.2(3)

Table 7 Hydrogen Atom Coordinates (Å×10^4) and Isotropic Displacement Parameters (Å^2×10^3) for compound **1**.

Atom	*x*	*y*	*z*	U(eq)
H2A	-13146	-2645	-1005	33
H2B	-13440	-4216	-146	19
H3A	-14115	-5632	-622	18
H5A	-15075	-5291	132	21
H5B	-15686	-6596	124	21
H6A	-12956	-6018	106	23
H6B	-13563	-7323	117	23
H7A	-12403	-6780	-348	19
H9A	-14375	-8654	-781	25
H9B	-14510	-7292	-846	25
H10A	-11946	-8100	-666	27
H10B	-12371	-8377	-981	27
H11A	-12568	-6328	-1066	24
H13A	-10387	-4238	-818	23
H13B	-11087	-4618	-1104	23
H14A	-13261	-4385	-886	20
H15A	-11074	-2576	-524	23
H16A	-12089	-1000	-298	43
H16B	-13347	-1792	-213	43
H16C	-13081	-1484	-534	43
H17A	-10473	-2384	-60	43
H17B	-11738	-3137	39	43
H17C	-10545	-3750	-130	43

H18A	-16481	-4369	-275	28
H18B	-17403	-5506	-247	28
H18C	-16780	-5158	-543	28
H19A	-14598	-8641	-69	39
H19B	-15011	-9299	-350	39
H19C	-13462	-9160	-269	39
H20A	-10375	-5638	-413	30
H20B	-10373	-6979	-507	30
H20C	-9315	-6086	-640	30
H2E	-13090	-2229	-4304	35
H2F	-13158	-4512	-3454	21
H3C	-13780	-5468	-3996	20
H5E	-14745	-5895	-3248	31
H5F	-15267	-7187	-3327	31
H6E	-12587	-6475	-3306	32
H6F	-13106	-7782	-3370	32
H7C	-11970	-6739	-3788	25
H9E	-13883	-8194	-4323	31
H9F	-14130	-6820	-4301	31
H10E	-11473	-7607	-4194	34
H10F	-11956	-7604	-4512	34
H11C	-12343	-5541	-4480	26
H13E	-10368	-3592	-4121	25
H13F	-11076	-3722	-4419	25
H14C	-13214	-3956	-4180	20
H15C	-11265	-2216	-3752	26
H16G	-12448	-1139	-3422	57
H16H	-13442	-2211	-3365	57
H16I	-13469	-1554	-3660	57
H17G	-10490	-2424	-3294	54
H17H	-11476	-3507	-3243	54
H17I	-10256	-3623	-3458	54
H18G	-16214	-4682	-3605	35
H18H	-17058	-5858	-3648	35
H18I	-16410	-5214	-3911	35
H19G	-13934	-8968	-3635	52
H19H	-14350	-9328	-3946	52
H19I	-12801	-9167	-3867	52
H20G	-10082	-5434	-3819	39
H20H	-9887	-6567	-4010	39
H20I	-9032	-5412	-4072	39
H2C	-11892	-6995	-2766	56
H2D	-11656	-5800	-1889	23
H3B	-11084	-4073	-2310	19
H5C	-10006	-4879	-1583	24
H5D	-9459	-3562	-1545	24
H6C	-12167	-4231	-1574	24
H6D	-11620	-2928	-1513	24
H7B	-12803	-3180	-1989	21
H9C	-10991	-949	-2343	28
H9D	-10731	-2221	-2468	28
H10C	-13350	-1735	-2258	30
H10D	-12961	-1219	-2558	30
H11B	-12590	-3170	-2720	29
H13C	-14642	-5489	-2564	36
H13D	-13929	-4885	-2826	36
H14B	-11777	-5237	-2618	31
H15B	-13811	-7376	-2325	40
H16D	-12640	-8968	-2156	56
H16E	-11515	-8148	-2021	56
H16F	-11641	-8247	-2353	56
H17D	-14340	-7877	-1865	93

H17E	-13190	-7035	-1748	93
H17F	-14479	-6491	-1899	93
H18D	-8666	-5495	-2031	27
H18E	-7759	-4378	-1963	27
H18F	-8418	-4523	-2265	27
H19D	-10662	-1475	-1647	43
H19E	-10243	-633	-1899	43
H19F	-11794	-829	-1827	43
H20D	-14700	-4325	-2096	35
H20E	-14922	-2971	-2165	35
H20F	-15827	-3940	-2315	35

Experimental

Single crystals of $C_{20}H_{32}O_2$ [exp_1415] were recrystallised from [solvents] mounted in inert oil and transferred to the cold gas stream of the diffractometer.

Crystal structure determination of [exp_1415]

Crystal Data. $C_{20}H_{32}O_2$, M =304.46, orthorhombic, a = 9.9686(2) Å, b = 11.4498(3) Å, c = 47.9528(18) Å, U = 5473.2(3) Å^3, T = 97.3, space group P2$_1$2$_1$2$_1$ (no. 19), Z = 12, μ(Cu Kα) = 0.532, 20784 reflections measured, 10157 unique (R_{int} = 0.0455) which were used in all calculations. The final $wR(F_2)$ was 0.1279 (all data).

This report has been created with Olex2, compiled on 2011.02.15 svn.r1672. Please let us know if there are any errors or if you would like to have additional featrues.

Figure S34. ORTEP depiction for X-ray crystal structure of **2**.

compound 2

Crystal Submitted by:
Crystal Submitted on:
Data Collected on:
Structure Solved by:

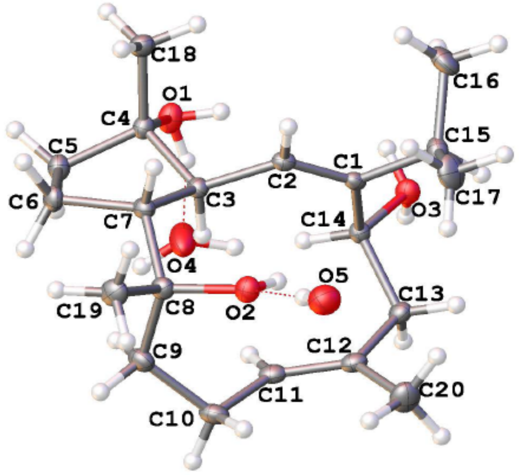

Table 1: Crystal data and structure refinement for compound 2

Identification code	exp_1423
Empirical formula	$C_{20}H_{36}O_4$
Formula weight	340.49
Temperature/K	98.0
Crystal system	monoclinic
Space group	C2
a/Å, b/Å, c/Å	16.6326(11), 9.5624(4), 13.5122(10)
α/°, β/°, γ/°	90.00, 115.405(7), 90.00
Volume/Å^3	1941.3(2)
Z	4
ρ_{calc}/mg mm^{-3}	1.165
μ/mm^{-1}	0.626
F(000)	752
Crystal size/mm^3	0.40 × 0.38 × 0.32
2Θ range for data collection	7.24 to 143.6°
Index ranges	$-20 \leq h \leq 19$, $-11 \leq k \leq 11$, $-16 \leq l \leq 14$
Reflections collected	8710
Independent reflections	3737[R(int) = 0.0169 (inf-0.9Å)]
Data/restraints/parameters	3737/3/231
Goodness-of-fit on F^2	1.046
Final R indexes [I>2σ (I) i.e. F_o>4σ (F_o)]	R_1 = 0.0338, wR_2 = 0.0898
Final R indexes [all data]	R_1 = 0.0341, wR_2 = 0.0901
Largest diff. peak/hole/e Å^{-3}	0.267/-0.172
Flack Parameters	0.13(13)
Completeness	0.991

Table 2 Fractional Atomic Coordinates ($\times 10^4$) and Equivalent Isotropic Displacement Parameters ($Å^2 \times 10^3$) for compound **2**. U_{eq} is defined as 1/3 of of the trace of the orthogonalised U_{IJ} tensor.

Atom	*x*	*y*	*z*	U(eq)
O1	5040.6(6)	-8558.2(11)	3339.3(7)	20.5(2)
O2	1460.5(6)	-7579.9(10)	1250.5(7)	18.5(2)
O3	4336.2(6)	-6227.8(10)	5356.1(7)	17.3(2)
C1	3387.1(8)	-6038.8(13)	3431.7(10)	12.9(2)
C2	3342.0(8)	-6590.3(13)	2500.1(10)	13.6(2)
C3	3435.5(8)	-8100.4(13)	2244.9(10)	13.8(3)
C4	4338.3(8)	-8523.1(13)	2249.4(9)	14.8(3)
C5	4125.1(9)	-9996.8(14)	1755.8(11)	18.4(3)
C6	3189.7(9)	-9858.9(14)	811.8(10)	18.7(3)
C7	2747.4(8)	-8571.1(14)	1093(1)	15.2(2)
C8	1794.2(8)	-8839.7(14)	977.7(10)	16.6(3)
C9	1737.0(9)	-10117.9(15)	1669.4(11)	20.4(3)
C10	1428.2(9)	-9798.4(15)	2571.1(11)	20.3(3)
C11	2113.8(9)	-8980.6(14)	3504.4(10)	17.0(3)
C12	2024.6(8)	-7755.5(14)	3921.3(10)	16.8(3)
C13	2831.7(8)	-7030.0(14)	4789.8(10)	16.7(3)
C14	3606.9(8)	-6895.2(13)	4462.8(10)	13.7(2)
C15	3239.1(9)	-4470.9(13)	3519.8(11)	18.3(3)
C16	3980.4(11)	-3597.9(16)	3437.4(13)	27.9(3)
C17	2330.1(11)	-3980.9(16)	2656.0(14)	29.2(3)
C18	4642.0(9)	-7554.5(15)	1577.5(11)	19.1(3)
C19	1180.7(9)	-9061.2(16)	-235.6(11)	22.3(3)
C20	1152.7(9)	-7011.7(18)	3594.7(13)	28.6(3)
O4	5000	-10165.5(17)	5000	38.9(4)
O5	0	-5745.6(18)	0	30.4(3)

Table 3 Anisotropic Displacement Parameters ($Å^2 \times 10^3$) for compound **2**. The Anisotropic displacement factor exponent takes the form: $-2\pi^2[h^2a^{*2}U_{11}+...+2hka \times b \times U_{12}]$

Atom	U_{11}	U_{22}	U_{33}	U_{23}	U_{13}	U_{12}
O1	17.6(4)	24.2(5)	15.2(4)	-1.9(4)	2.8(4)	4.7(4)
O2	16.9(4)	21.1(5)	14.5(4)	-1.3(4)	4.0(3)	1.3(4)
O3	14.4(4)	19.8(4)	14.4(4)	-1.7(3)	3.0(3)	-0.1(3)
C1	11.2(5)	11.7(6)	15.6(6)	0.4(4)	5.4(4)	-1.2(4)
C2	12.1(5)	14.4(6)	12.9(5)	2.0(5)	4.1(4)	-0.7(4)
C3	15.4(6)	13.8(6)	12.1(5)	-0.2(4)	5.6(5)	-1.0(4)
C4	14.6(6)	15.8(6)	13.1(5)	-0.4(5)	5.2(5)	1.5(5)
C5	20.8(6)	16.3(6)	17.9(6)	-1.8(5)	8.1(5)	1.8(5)
C6	20.2(6)	18.7(6)	17.0(6)	-6.1(5)	7.7(5)	-1.0(5)
C7	16.6(6)	15.7(6)	13.2(5)	-1.6(5)	6.3(5)	-2.5(5)
C8	15.5(6)	18.3(6)	14.7(6)	-2.5(5)	5.3(5)	-2.4(5)
C9	21.8(6)	18.8(6)	19.5(6)	-3.6(5)	7.8(5)	-6.7(5)
C10	19.2(6)	21.9(7)	19.1(6)	0.8(5)	7.5(5)	-7.2(5)
C11	16.0(6)	20.5(6)	15.1(6)	3.5(5)	7.3(5)	-2.2(5)
C12	15.3(6)	21.2(6)	15.2(6)	2.0(5)	7.8(5)	-1.9(5)
C13	17.8(6)	19.1(7)	15.4(6)	-0.7(5)	9.2(5)	-0.7(5)
C14	13.1(5)	13.3(5)	13.1(6)	-0.4(4)	4.1(4)	0.5(4)
C15	25.1(6)	14.2(6)	15.3(6)	-0.6(5)	8.5(5)	1.2(5)
C16	35.6(8)	15.3(6)	30.9(7)	-1.1(6)	12.4(6)	-6.4(6)
C17	30.7(8)	19.2(7)	32.8(8)	2.0(6)	9.1(6)	10.6(6)
C18	16.9(6)	20.6(6)	20.9(6)	0.1(5)	9.3(5)	-1.0(5)
C19	18.8(6)	27.6(7)	16.8(6)	-5.1(5)	4.1(5)	-4.2(6)
C20	18.5(6)	32.6(8)	32.8(8)	-4.8(6)	9.2(6)	1.2(6)
O4	72.7(12)	20.1(7)	40.6(9)	0	40.2(9)	0
O5	29.0(8)	31.2(8)	31.7(8)	0	13.9(6)	0

Table 4 Bond Lengths for compound **2**.

Atom	Atom	Length/Å	Atom	Atom	Length/Å
O1	C4	1.4347(14)	C8	C19	1.5292(17)
O2	C8	1.4391(16)	C9	C10	1.5423(19)
C1	C15	1.5323(17)	C11	C10	1.5062(18)
C2	C1	1.3368(18)	C12	C11	1.335(2)
C2	C3	1.5081(17)	C12	C13	1.5195(17)
C4	C3	1.5526(17)	C12	C20	1.5015(19)
C5	C4	1.5341(17)	C14	O3	1.4419(15)
C5	C6	1.5369(18)	C14	C1	1.5194(17)
C7	C3	1.5513(16)	C14	C13	1.5372(16)
C7	C6	1.5627(17)	C16	C15	1.532(2)
C7	C8	1.5465(16)	C17	C15	1.5342(19)
C8	C9	1.5662(18)	C18	C4	1.5265(17)

Table 5 Bond Angles for compound **2**.

Atom	Atom	Atom	Angle/°	Atom	Atom	Atom	Angle/°
C2	C1	C14	123.03(11)	O2	C8	C9	111.81(10)
C2	C1	C15	120.88(11)	O2	C8	C19	103.72(10)
C14	C1	C15	116.08(11)	C7	C8	C9	113.05(11)
C1	C2	C3	128.56(11)	C19	C8	C7	108.35(10)
C2	C3	C4	116.29(10)	C19	C8	C9	110.38(11)
C2	C3	C7	113.78(10)	C10	C9	C8	116.29(12)
C7	C3	C4	102.97(10)	C11	C10	C9	112.03(11)
O1	C4	C3	111.73(9)	C12	C11	C10	128.86(13)
O1	C4	C5	111.06(10)	C11	C12	C13	120.34(12)
O1	C4	C18	107.05(10)	C11	C12	C20	124.03(12)
C5	C4	C3	101.22(10)	C20	C12	C13	115.63(12)
C18	C4	C3	113.95(10)	C12	C13	C14	112.86(10)
C18	C4	C5	111.88(11)	O3	C14	C1	108.31(10)
C4	C5	C6	104.17(10)	O3	C14	C13	107.74(10)
C5	C6	C7	106.28(10)	C1	C14	C13	114.03(10)
C3	C7	C6	104.53(10)	C1	C15	C17	112.24(11)
C8	C7	C3	115.63(10)	C16	C15	C1	111.49(11)
C8	C7	C6	114.45(10)	C16	C15	C17	109.85(12)
O2	C8	C7	109.06(10)				

Table 6 Torsion Angles for compound **2**.

A	B	C	D	Angle/°
O1	C4	C3	C2	71.96(13)
O1	C4	C3	C7	-162.92(10)
O2	C8	C9	C10	-5.46(15)
O3	C14	C1	C2	126.98(12)
O3	C14	C1	C15	-51.52(13)
O3	C14	C13	C12	-178.56(10)
C1	C2	C3	C4	-103.14(14)
C1	C2	C3	C7	137.44(13)
C1	C14	C13	C12	61.19(14)
C2	C1	C15	C16	-66.41(16)
C2	C1	C15	C17	57.31(16)
C3	C2	C1	C14	3.9(2)
C3	C2	C1	C15	-177.68(12)
C3	C7	C6	C5	-4.30(13)
C3	C7	C8	O2	58.80(14)
C3	C7	C8	C9	-66.25(14)
C3	C7	C8	C19	171.07(11)
C4	C5	C6	C7	-23.56(13)

C5	C4	C3	C2	-169.78(10)
C5	C4	C3	C7	-44.67(11)
C6	C5	C4	O1	160.79(10)
C6	C5	C4	C3	42.05(12)
C6	C5	C4	C18	-79.66(13)
C6	C7	C3	C2	156.85(10)
C6	C7	C3	C4	30.12(12)
C6	C7	C8	O2	-179.62(10)
C6	C7	C8	C9	55.33(14)
C6	C7	C8	C19	-67.34(14)
C7	C8	C9	C10	118.09(12)
C8	C7	C3	C2	-76.39(14)
C8	C7	C3	C4	156.88(10)
C8	C7	C6	C5	-131.79(11)
C8	C9	C10	C11	-69.59(15)
C11	C12	C13	C14	50.81(16)
C12	C11	C10	C9	124.28(15)
C13	C12	C11	C10	-173.35(11)
C13	C14	C1	C2	-113.09(13)
C13	C14	C1	C15	68.41(14)
C14	C1	C15	C16	112.12(12)
C14	C1	C15	C17	-124.15(12)
C18	C4	C3	C2	-49.53(14)
C18	C4	C3	C7	75.58(13)
C19	C8	C9	C10	-120.37(12)
C20	C12	C11	C10	7.3(2)
C20	C12	C13	C14	-129.74(13)

Table 7 Hydrogen Atom Coordinates (Å×10^4) and Isotropic Displacement Parameters (Å^2×10^3) for compound **2**.

Atom	*x*	*y*	*z*	U(eq)
H1A	4846	-8852	3763	31
H1B	5028	-7840	3664	31
H2A	1798	-7333	1872	28
H2B	3239	-5959	1935	16
H3A	4761	-6153	5202	5(6)
H3B	4508	-6733	5899	26
H3C	3348	-8679	2789	17
H5B	4553	-10288	1485	22
H5C	4128	-10669	2295	22
H6A	3227	-9711	123	22
H6B	2845	-10699	752	28
H7	2708	-7824	578	18
H9A	1332	-10799	1172	24
H9B	2320	-10551	2011	24
H10A	878	-9269	2257	24
H10B	1309	-10671	2850	24
H11	2676	-9384	3836	20
H13A	2660	-6104	4921	20
H13B	3032	-7553	5469	20
H14	3793	-7833	4355	16
H15	3255	-4300	4243	22
H16A	3891	-2628	3545	42
H16B	4546	-3896	3990	42
H16C	3968	-3723	2726	42
H17A	2250	-3009	2768	44
H17B	2303	-4109	1938	44
H17C	1868	-4518	2721	44
H18A	4718	-6625	1872	29
H18B	5197	-7884	1608	29

H18C	4202	-7546	830	29
H19A	1201	-8252	-645	33
H19B	1374	-9867	-499	33
H19C	582	-9203	-325	33
H20A	1210	-6061	3406	43
H20B	702	-7474	2974	43
H20C	989	-7025	4195	43
H4A	4950.0(4)	-9610.0(4)	5470.0(3)	58
H4B	5000	-11065(3)	5000	58
H5A	441	-6277	407	46

Experimental

Single crystals of $C_{20}H_{36}O_4$ [exp_1423] were recrystallised from [solvents] mounted in inert oil and transferred to the cold gas stream of the diffractometer.

Crystal structure determination of [exp_1423]

Crystal Data. $C_{20}H_{36}O_4$, M =340.49, monoclinic, a = 16.6326(11) Å, b = 9.5624(4) Å, c = 13.5122(10) Å, β = 115.405(7)°, U = 1941.3(2) Å^3, T = 98.0, space group C2 (no. 5), Z = 4, μ(Cu Kα) = 0.626, 8710 reflections measured, 3737 unique (R_{int} = 0.0169) which were used in all calculations. The final $wR(F_2)$ was 0.0901 (all data).

This report has been created with Olex2, compiled on 2011.02.15 svn.r1672. Please let us know if there are any errors or if you would like to have additional featrues.
